# Influenza A Virus (H1N1) Infection Induces Ferroptosis to Promote Developmental Injury in Fetal Tissues

**DOI:** 10.1111/cpr.70117

**Published:** 2025-08-26

**Authors:** Yuxi Jiang, Yao Shen, Qiongyin Zhang, Zi Liu, Yuzhen Liu, Jiaojiao Peng, Xuesong Yang, Feng Gao, Xiang‐Hong Ou, Qing‐Yuan Sun, Qiao Zhang, Guang Wang

**Affiliations:** ^1^ Guangdong‐Hong Kong Metabolism & Reproduction Joint Laboratory Guangdong Second Provincial General Hospital, School of Medicine, Jinan University Guangzhou China; ^2^ International Joint Laboratory for Embryonic Development & Prenatal Medicine, Division of Histology and Embryology School of Medicine, Jinan University Guangzhou China; ^3^ Key Laboratory for Regenerative Medicine of the Ministry of Education Jinan University Guangzhou China; ^4^ Institute of Molecular and Medical Virology School of Medicine, Jinan University Guangzhou China; ^5^ International School Guangzhou Huali College Zengcheng, Guangzhou China; ^6^ Key Laboratory of Viral Pathogenesis & Infection Prevention and Control (Jinan University), Ministry of Education Guangzhou China

**Keywords:** ferroptosis, fibrosis, H1N1 virus, maternal infection, oxidative stress

## Abstract

H1N1, a globally pervasive subtype of influenza A virus (IAV), poses an ongoing threat to human health and occasionally leads to multi‐organ dysfunction in severe cases. Evidence confirms that the H1N1 virus is enabled to penetrate the placental barrier; however, the underlying mechanisms by which maternal infection contributes to detrimental fetal outcomes remain elusive. In this study, a systematic literature review and meta‐analysis demonstrated a strong association between maternal H1N1 infection during pregnancy and adverse fetal outcomes. Using a chicken embryo model, we found that the H1N1 virus specifically targets the developing liver and lung tissues, activates immune and stromal cells, and induces localised inflammatory responses, thereby triggering excessive oxidative stress. The resulting imbalance in oxidative stress disrupts antioxidant defence systems and promotes ferroptosis in parenchymal cells. Persistent ferroptosis subsequently initiates tissue repair processes, activates fibroblasts, and leads to aberrant extracellular matrix deposition, ultimately contributing to early fibrosis in the liver and lung tissues. Collectively, this study elucidates the molecular mechanisms by which H1N1 selectively infects fetal liver and lung, inducing ferroptosis‐mediated parenchymal cell death and tissue fibrosis, thereby impairing fetal development. These findings provide novel theoretical insights for the clinical management and prevention of H1N1‐associated maternal‐fetal infections and adverse pregnancy outcomes.

## Graphical abstract

H1N1 infection during pregnancy specifically targets the fetal liver and lungs, triggering immune responses, tissue fibrosis and ferroptosis in parenchymal cells, ultimately leading to adverse pregnancy outcomes, offering insights into managing maternal H1N1 infections and their impact on fetal health.

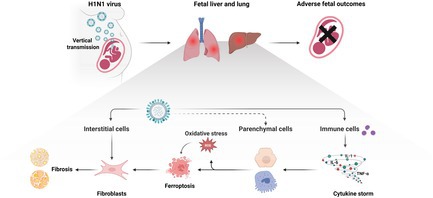

## 1 Introduction

The World Health Organization has identified influenza pandemics as one of the top 10 global health threats [1]. As a major subtype of influenza A virus, H1N1 has caused several worldwide outbreaks since the 1918 Spanish flu [2], including the 1957 Asian flu, the 1968 Hong Kong flu, and the 2009 novel influenza pandemic, cumulatively resulting in the deaths of millions of people [3]. In recent years, H1N1 strains have continued to circulate seasonally, posing particular risks to children, the elderly, and individuals with underlying health conditions. Its characteristics of antigenic drift, interspecies transmission, and respiratory droplet spread confer high transmissibility and pathogenicity [4, 5].

Infection with the H1N1 subtype of influenza A virus induces a complex host response characterised by the hijacking of host cellular machinery to promote viral replication, ultimately leading to cell lysis and death [6]. Beyond direct cytopathic effects, extensive viral proliferation within respiratory tissues under hypoxic and pathological conditions induces severe tissue damage and triggers excessive immune activation [7]. This overactivation manifests as the so‐called “cytokine storm,” marked by the heightened release of proinflammatory cytokines such as interleukin‐6 (IL‐6), interleukin‐1β (IL‐1β), and tumour necrosis factor‐α (TNF‐α) [8]. The NF‐κB signalling pathway, as a central regulator of this inflammatory cascade, plays a pivotal role in coordinating the transcription of these inflammatory mediators [9, 10]. Sustained immune cell infiltration and inflammatory signalling can induce multiple forms of programmed cell death, leading to progressive tissue damage, disrupted repair processes, and ultimately pathological fibrosis [11]. This continuous pathogenic cascade, which progresses from viral infection and inflammatory response to cell death and fibrosis, has been well documented in various viral infection models [12].

In recent years, it has been discovered that, in addition to classical apoptosis and necroptosis, a novel form of programmed cell death known as ferroptosis, which is driven by iron‐dependent lipid peroxidation, can also be triggered during various viral infections [13]. Viruses may suppress glutathione peroxidase 4 (GPX4) activity, deplete intracellular glutathione (GSH) levels, modulate iron uptake‐related proteins such as transferrin receptor 1 (TfR1), and disrupt the system Xc^−^/GPX4 axis, leading to the accumulation of intracellular free iron and lipid peroxides, ultimately inducing ferroptosis. Ferroptosis not only contributes to virus‐associated cytopathic damage but is also closely associated with viral‐induced tissue fibrosis and immunopathological responses [14], making it a potential novel target for antiviral therapy. Existing studies have shown that H1N1 can disrupt intracellular iron homeostasis by interfering with TfR1‐mediated iron uptake pathways, thereby promoting viral invasion and replication [15]. However, the specific mechanisms by which it induces ferroptosis and its role in virus‐associated organ damage, particularly in relation to fetal developmental abnormalities, remain unclear.

During pregnancy, increased metabolic demands and immune tolerance render pregnant women more susceptible to H1N1 infection, with severe cases often leading to multiple organ damage and adverse pregnancy outcomes [16]. Embryonic development is highly sensitive to the maternal microenvironment, and viral infections, pharmacological agents, or other stressors can disrupt normal development, potentially resulting in miscarriage or congenital malformations [17, 18]. Previous studies have confirmed that H1N1 can cross the placental barrier and affect fetal organ development [19, 20], though the specific mechanisms of its effects on the liver and lungs remain to be elucidated.

Due to its operational convenience, the high degree of genetic similarity between chickens and humans, the low‐cost chick embryo model has been widely employed in studies of viral pathogenesis [21, 22]. Based on this, the present study utilised chick embryos as a model to investigate the effects of H1N1 infection on fetal organ development. The results demonstrated that the H1N1 virus exhibits marked cellular tropism for the liver and lungs, accompanied by immune cell infiltration, stromal activation, and ferroptosis, resulting in parenchymal cell dysfunction and early‐stage fibrosis. These findings provide experimental evidence for elucidating the molecular mechanisms underlying H1N1‐induced fetal organ injury and contribute to the development of maternal‐fetal intervention strategies.

## 2 Results

### 2.1 Meta‐Analysis Shows That Maternal H1N1 Virus Infection Leads to Adverse Fetal Outcomes

**FIGURE 1:**
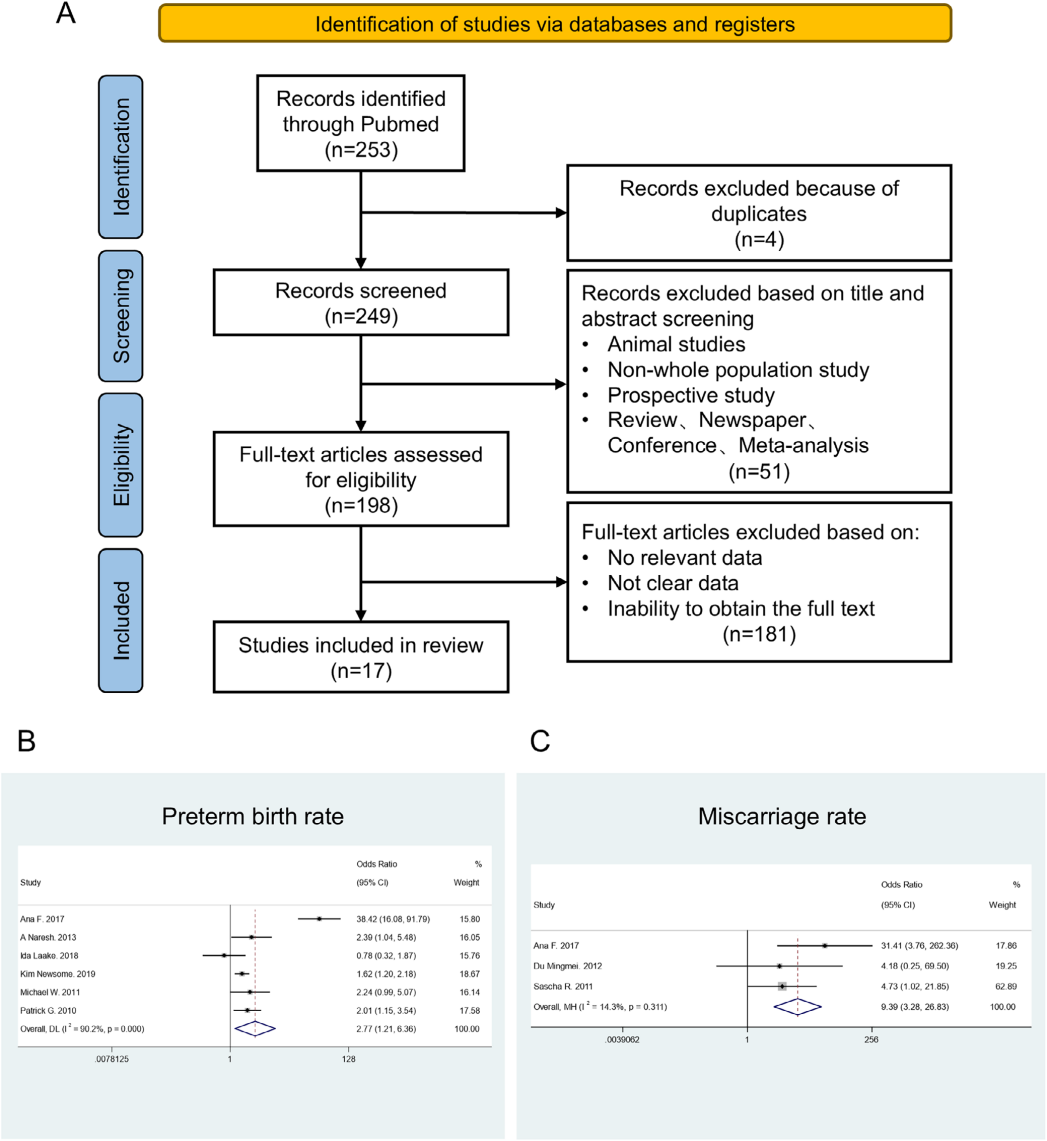
Meta‐analysis for the correlation between H1N1 infection and Preterm birth/Miscarriage. (A) Flow chart of systematic literature search in PubMed with the following search terms: ((“Pregnant Women”[Mesh]) OR (Woman, Pregnant) OR (Pregnant Woman) OR (Women, Pregnant) OR (“Pregnancy”[Mesh]) OR (Gestation) OR (Pregnancies)) AND ((“Influenza A Virus, H1N1 Subtype”[Mesh]) OR (H1N1 subtype) OR (H1N1 subtypes) OR (subtype, H1N1) OR (H1N1 Virus) OR (H1N1 Viruses) OR (Virus, H1N1) OR (H1N1 Influenza Virus) OR (H1N1 Influenza Viruses) OR (Influenza Virus, H1N1) OR (Virus, H1N1 Influenza) OR (Swine‐Origin Influenza A H1N1 Virus) OR (Swine Origin Influenza A H1N1 Virus) OR (Influenza A (H1N1)pdm09 Virus) OR (Influenza A (H1N1)pdm09) OR (Influenza A H1N1, Variant Virus) OR (H1N1v Viruses) OR (H1N1v Virus) OR (Virus, H1N1v) OR (H1N1)) AND ((clinical features) OR (clinical characteristics) OR (clinical outcomes)). The search period ranges from January 2009 to October 2024. B‐C: Forest plots for the effect of H1N1 infection on: Preterm birth (B) and Miscarriage (C).

To systematically assess the correlation between maternal H1N1 infection and fetal outcomes, we first performed a meta‐analysis on the literature available in PubMed (Figure 1A). The effect size and 95% confidence interval for each variant were estimated. In the final selection of 17 studies (Figure 1A and Table S2), by extracting key data and conducting a meta‐analysis, we found that pregnant women infected with H1N1 have higher rates of severe illness and mortality compared to non‐pregnant women of reproductive age (Figure S1). Additionally, severe cases in pregnant women have a higher rate of miscarriage and preterm birth compared to mild cases (Figure 1B,C). This indicates that pregnant women are a high‐risk group for H1N1 infection, and maternal H1N1 infection is a risk factor for adverse fetal outcomes.

### 2.2 H1N1 Virus Primarily Infects the Liver and Lungs During Development

**FIGURE 2:**
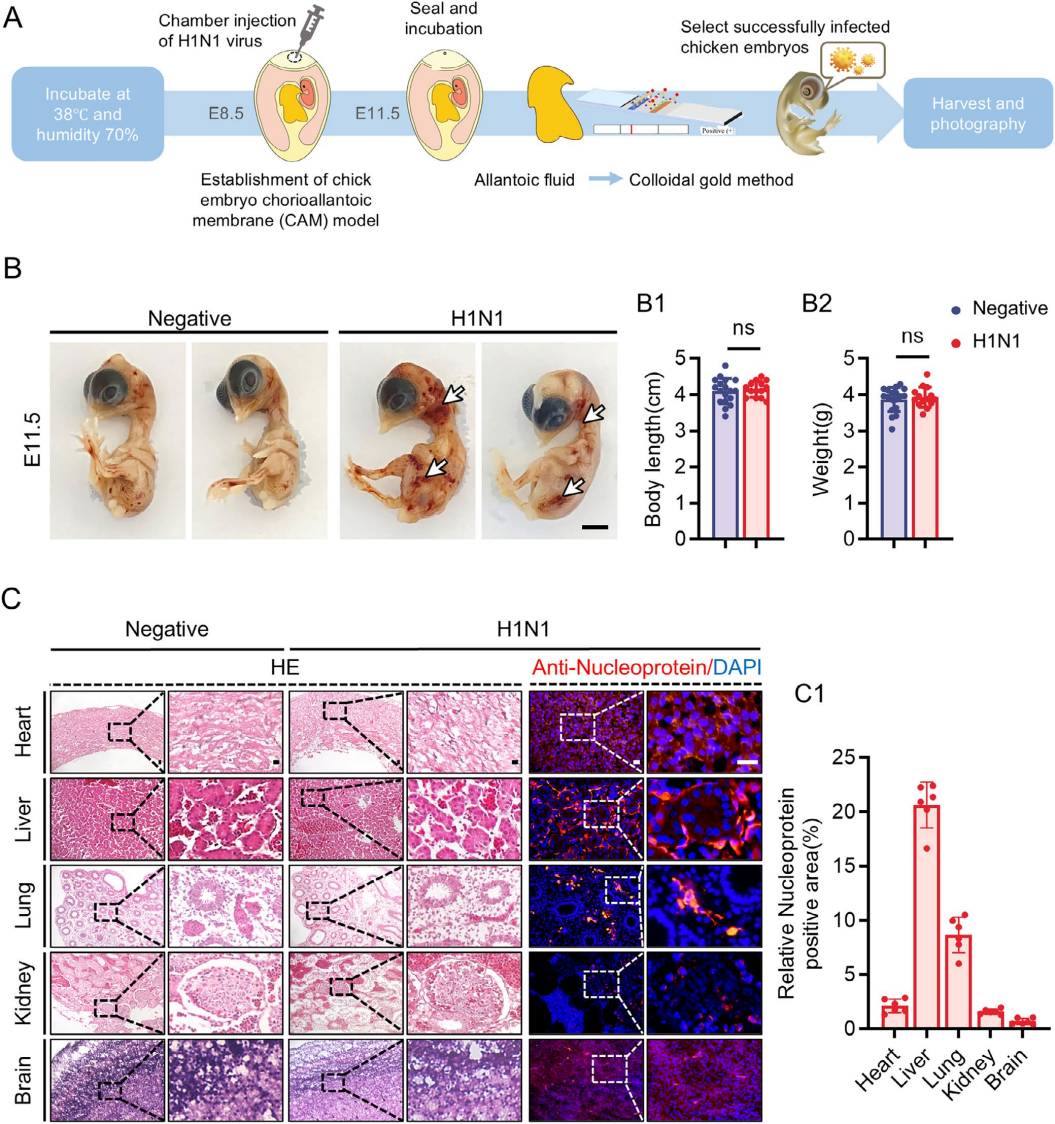
Effects of H1N1 virus infection on the development of chicken embryos. (A) The sketch illustrates the method of establishment of a disease model of H1N1 virus infection in chicken embryos. (B‐B2) Gross images of E11.5 chicken embryos from the negative group and the H1N1 virus infection group (B), with arrows indicating localised congestion. Bar charts indicated the body length (B1) and body weight (B2) of chicken embryos in both the negative and H1N1 virus infection groups. (C‐C1) Representative cross‐sectional images of the heart, liver, lungs, kidneys, and brain from E11.5 chicken embryos of the negative group and the H1N1 virus infection group, stained with HE and anti‐Nucleoprotein immunofluorescence labelled for H1N1 virus (C). The bar chart shows the ratio of the anti‐Nucleoprotein‐positive area to the total DAPI‐labelled area across different organs in the H1N1 virus‐infected group (C1). Scale bars = 1 cm in B; 20 μm in C. *n* ≥ 14 (B), *n* = 3 (C). ns: Not significant.

To investigate the effects of H1N1 infection on the foetus, we established a disease model using H1N1‐infected chick embryos (Figure 2A). Initially, we observed the overall morphology of the embryos and noted localised congestion in the neck and abdominal regions of the H1N1‐infected group (Figure 2B), suggesting the virus could enter the bloodstream and trigger inflammatory responses. However, there were no significant changes in the body length or weight of the embryos (Figure 2B1,B2). We then examined the major organs of the embryos to assess the impact of the virus infection. HE staining and immunofluorescence detection using virus‐specific nucleoprotein antibody (anti‐Nucleoprotein) revealed that the virus predominantly accumulated in the liver and lungs of the chicken embryos (Figures 2C,C1 and S2). By comparing adjacent sections of HE staining and immunofluorescence positive signal, we found that the virus predominantly concentrated in the blood vessels of the lungs and liver (Figure S2B,B1). While the effects of H1N1 on the lungs have been widely studied as a respiratory virus, there is limited evidence regarding its potential harm to the liver. To address this, we reviewed retrospective studies on hospitalised H1N1 patients (Figure S3A and Table S3) and confirmed that severe cases exhibited significantly abnormal liver enzyme levels and a higher risk of liver damage (Figure S3B–D).

### 2.3 H1N1 Virus Induces Inflammatory Infiltration in Chicken Embryos

**FIGURE 3:**
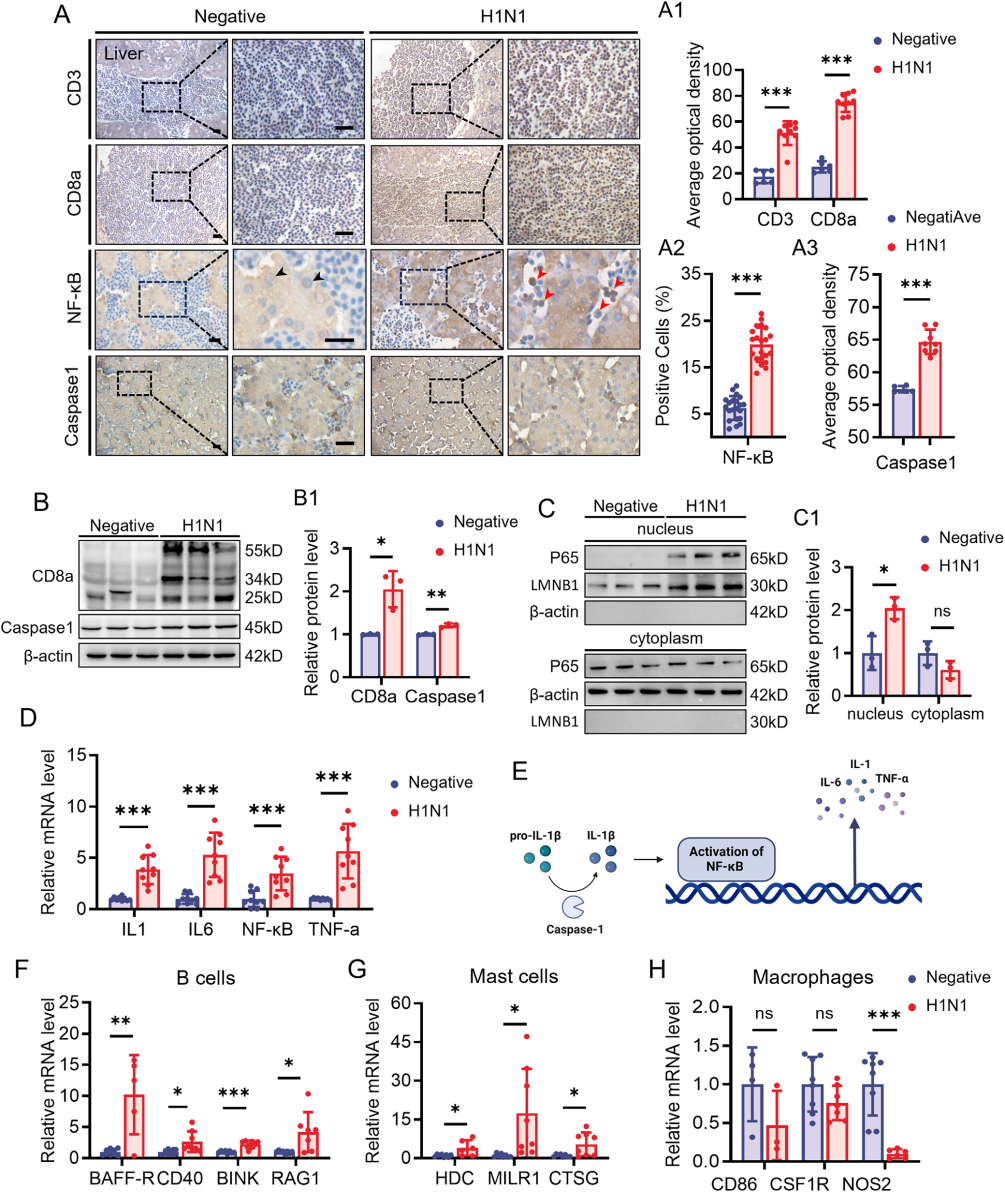
Assessment of immune infiltration after H1N1 virus infection in developmental liver. (A‐A2) Immunochemistry staining of CD3, CD8a, NF‐κB, and Caspase1 in the liver of E11.5 negative and H1N1 virus‐infected chicken embryos (A). The bar charts showing the corresponding optical density–based relevant quantification (A1‐A2). In the NF‐κB immunohistochemical images, black arrows indicate positively stained cells in the control group, while red arrows indicate positively stained cells in the H1N1‐infected group. (B‐B1) Western blot analysis showing the expression levels of CD8a and Caspase1 in the liver of negative and H1N1 virus‐infected chicken embryos (B), along with the respective quantitative analyses (B1). (C‐C1) Western blot analysis showing the expression levels of NF‐κB in both the nuclear and cytoplasmic fractions of liver cells (C), and the corresponding quantitative analyses (C1). (D) Quantitative PCR data illustrating the mRNA expression levels of IL‐1β, IL‐6, NF‐κB, and TNF‐α in the liver of negative and H1N1 virus‐infected chicken embryos. (E) Schematic diagram illustrating cytokine interactions after H1N1 virus infection. (F) Quantitative PCR data showing the mRNA expression levels of BAFF‐R, CD40, BINK, and RAG1 in the liver of the two groups. (G) Quantitative PCR data showing the mRNA expression levels of HDC, MILR1, and CTSG. (H) Quantitative PCR data showing the mRNA expression levels of CD86, CSF1R, and NOS2. Scale bars = 20 μm in A. *n* = 3 (A‐H). *p < 0.05, **p < 0.01, ***p < 0.001, ns: Not significant.

Viral infection often leads to inflammatory infiltration in the body. Given the liver's extensive vascularisation, which serves as a primary site for immune cell accumulation, we detected the immune response in the liver of H1N1‐infected chicken embryos. Immunohistochemical experiments revealed increased expression of T cell markers CD3 and CD8a (Figure 3A,A1), which was validated by Western blot analysis (Figure 3B,B1). Additionally, HE staining identified suspected immune cell aggregation (Figure S4A). Immunofluorescence results for the proliferation marker PCNA showed significantly higher proliferation activity of cells in the bloodstream compared to the control group, indicating an active immune response (Figure S4B,B1).

To further characterise changes in other immune cell populations, we assessed the expression of cell‐type‐specific markers. The results showed a marked upregulation of mast cell‐associated markers HDC, MILR1, and CTSG in the infected group (Figure 3F). Similarly, B cell‐related markers including BAFFR, CD40, BANK, and RAG1 were also elevated following infection (Figure 3G). In contrast, no significant changes were observed in the expression of macrophage markers CD86 and CSF1R, while the expression of the M1 macrophage marker NOS2 was downregulated (Figure 3H).

To verify the changes in proinflammatory mediators, we further analysed the expression of nuclear factor NF‐κB (P65) and its upstream and downstream associated molecules. Immunohistochemistry, western blotting, and RT‐qPCR analyses consistently demonstrated increased levels of NF‐κB in the infected group compared to controls, accompanied by elevated expression of inflammasome‐related components (IL‐1 and Caspase‐1) and NF‐κB downstream effectors (IL‐6 and TNF‐α) (Figure 3A–E). Enzyme‐linked immunosorbent assay (ELISA) results also confirmed significantly higher concentrations of IL‐1, IL‐6, and TNF‐α in liver tissues from the H1N1‐infected embryos relative to controls (Figures S8J and S9I). Furthermore, to determine whether NF‐κB translocated to the nucleus upon infection, we performed nuclear and cytoplasmic fractionation followed by western blot analysis of NF‐κB (P65). The results revealed a significant increase in nuclear NF‐κB expression in the H1N1‐infected group, while its cytoplasmic expression remained unchanged, indicating that viral infection induced nuclear translocation of NF‐κB, thereby promoting the inflammatory response (Figure 3C‐C1).

### 2.4 H1N1 Virus Infection Leads to Decreased Parenchymal Cell Viability and Early Fibrosis of Developing Liver and Lung

**FIGURE 4:**
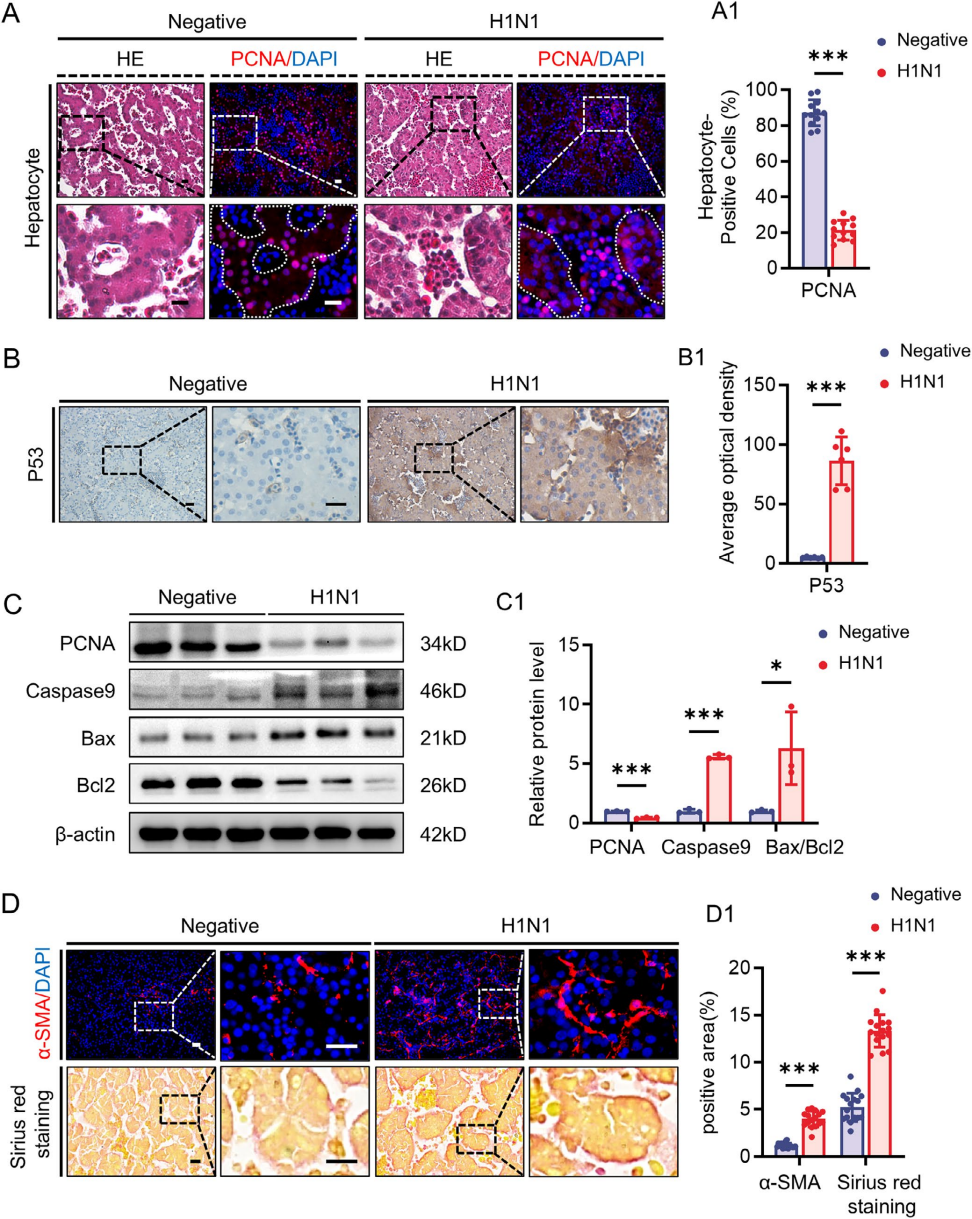
H1N1 virus infection leads to decreased liver viability and liver fibrosis during development. (A‐A1) Representative cross‐sectional images of HE staining and PCNA immunofluorescence staining of the liver in E11.5 negative group and H1N1 virus‐infected group chicken embryos (A). Dashed lines in the images indicate the specific regions of hepatocytes used for quantification. The bar charts showing the comparisons of PCNA‐positive cells in total DAPI‐labelled Hepatocytes (A1). (B‐B1) P53 immunohistochemical staining in the liver of E11.5 negative group and H1N1 virus‐infected group chicken embryos (B). The bar charts showing the corresponding optical density–based relevant quantification (B1). (C‐C1) Western blot showing the expression of PCNA, Caspase 9, Bcl2, and Bax in the liver of negative and H1N1 virus‐infected group chicken embryos (C), along with the corresponding quantitative analysis (C1). D‐D1: Representative cross‐sectional images of α‐SMA immunofluorescence staining and Sirius Red staining in the liver of E11.5 negative group and H1N1 virus‐infected group chicken embryos (D). The bar charts showing the comparisons of α‐SMA or Sirius Red staining positive areas in total areas (D1). Scale bars = 20 μm in A, B, D. *n* = 3 (A–D). *p < 0.05, ***p < 0.001.

Given that the H1N1 primarily targets the developing liver and lungs, we subsequently assessed cell viability in these organs. By comparing adjacent sections of HE staining and immunofluorescence, we found that H1N1 infection resulted in downregulation of the proliferation marker PCNA specifically in liver parenchymal cells, rather than in cells within the blood vessels (Figure 4A,A1), while the expression of the cell death‐related protein P53 significantly increased in these cells (Figure 4B,B1). Western blot analysis also indicated a decrease in total PCNA expression in the liver, along with an increased expression of caspase‐9 and a reduced ratio of Bax to Bcl‐2, suggesting substantial cell death in the liver (Figure 4C,C1). Furthermore, the fibroblast marker α‐SMA and Sirius Red staining showed activation of fibroblast and increased collagen content in the liver of H1N1‐infected embryos (Figure 4D,D1), indicating early fibrosis during liver development. Moreover, as the viral load increased, the expression level of α‐SMA also correspondingly elevated (Figure S5B‐B2). Additionally, we used Desmin immunofluorescence to assess the quantity of hepatic stellate cells, finding that H1N1 infection promotes an increase in these cells, with the Desmin and anti‐Nucleoprotein showing co‐expression (Figure S5A‐A1), suggesting that H1N1 can directly stimulate the activation of hepatic stellate cells.

**FIGURE 5:**
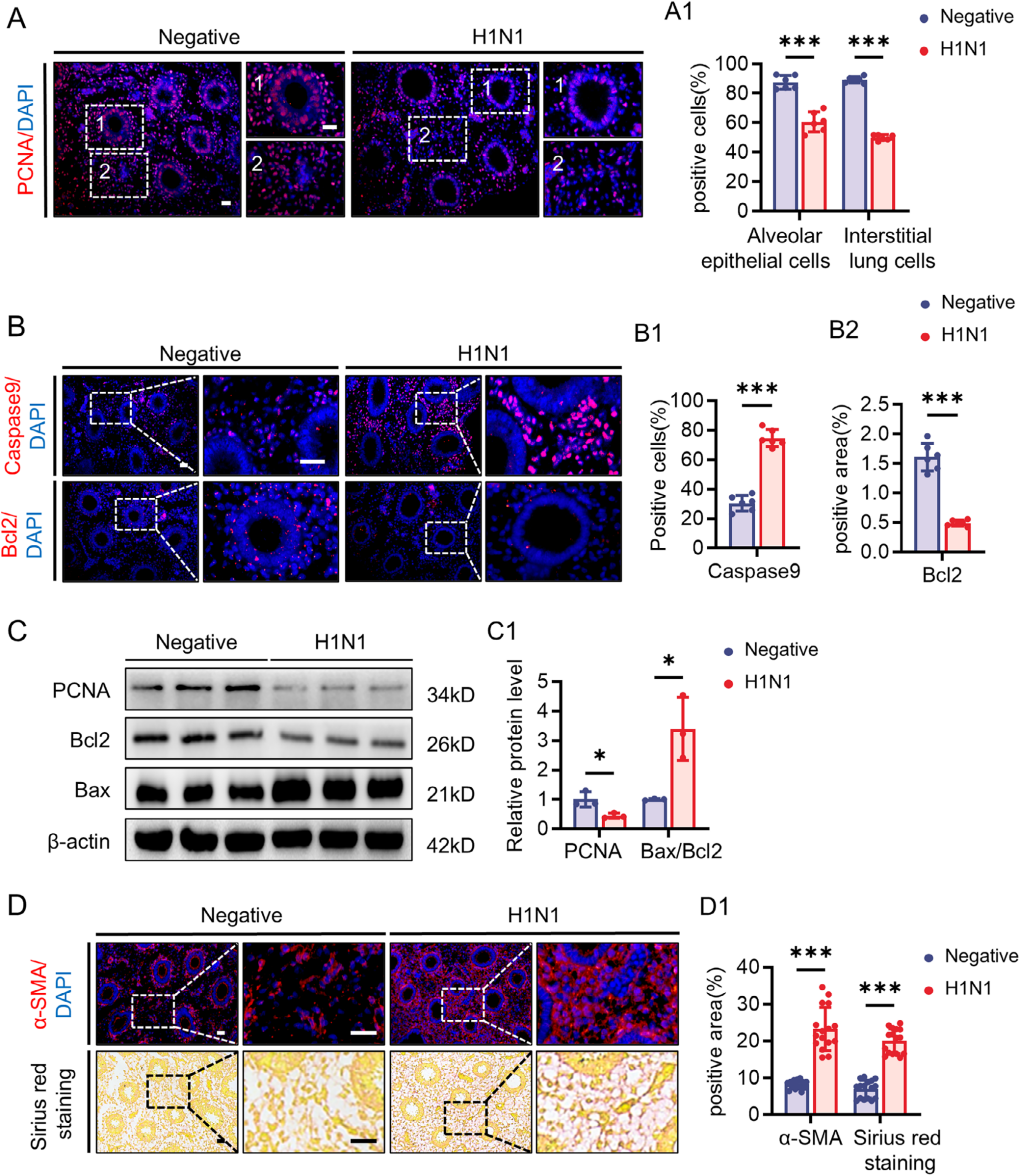
H1N1 virus infection leads to decreased lung viability and pulmonary fibrosis during development. (A‐A1) PCNA immunofluorescence staining in the lungs of E11.5 negative and H1N1 virus‐infected chicken embryos (A), along with corresponding quantitative analyses (A1). (B‐B2) Representative Caspase9 and Bcl2 immunofluorescence staining in the lungs of E11.5 negative and H1N1 virus‐infected chicken embryos (B), along with corresponding quantitative analyses (B1‐B2). (C‐C1) Western blot analysis showing the expression of PCNA, Bcl2, and Bax in the lungs of negative and H1N1 virus‐infected chicken embryos (C), along with corresponding quantitative analyses (C1). (D‐D1) Representative α‐SMA immunofluorescence staining and Sirius red staining in the lungs of E11.5 negative and H1N1 virus‐infected chicken embryos (D), along with corresponding quantitative analyses (D1). Scale bars = 20 μm in A, B, D. *n* = 3 (A–D). *p < 0.05, ***p < 0.001.

Similarly, we observed that H1N1 infection also led to downregulation of the proliferation marker PCNA in lung parenchymal cells, while the expression of the cell death‐related proteins caspase‐9 and Bax to Bcl‐2 ratio increased, accompanied by a decrease in Bcl‐2 expression (Figure 5A‐C1). The fibroblast marker α‐SMA was elevated, and Sirius Red staining indicated increased collagen content in the lungs (Figure 5D,D1), suggesting damage to developing lung parenchymal cells and early fibrosis in the developing lungs.

### 2.5 H1N1 Virus Infection Leads to Ferroptosis in Developing Liver and Lung Parenchymal Cells

**FIGURE 6:**
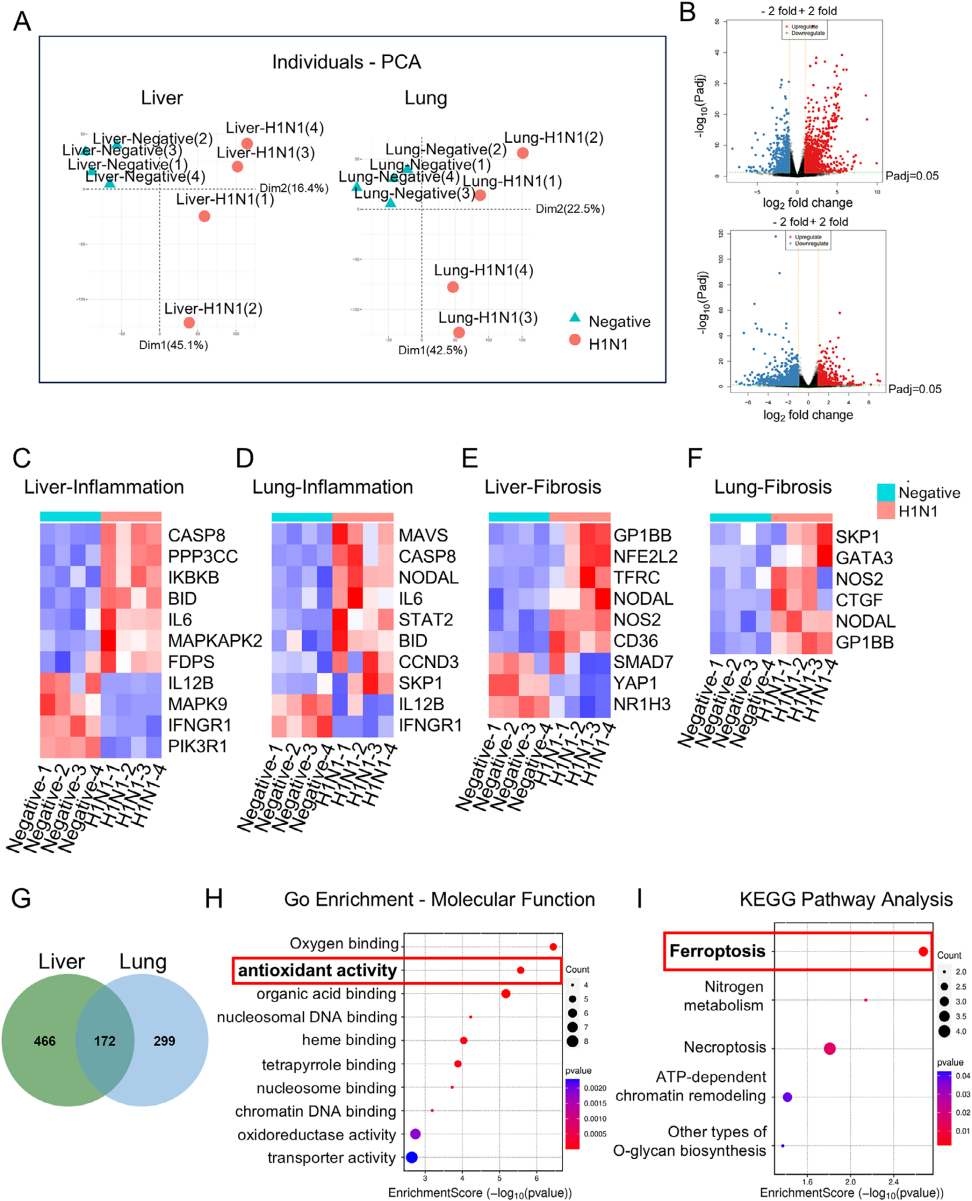
Bioinformatic analysis based on RNA‐seq data of liver and lung from E11.5 chicken embryos. (A) Principal component analysis of liver and lung of E11.5 negative and H1N1 virus‐infected chicken embryos. (B) Volcano plot of differentially expressed genes in liver and lung between the negative group and H1N1 virus‐infected group. (C, D) Heatmap analysis of inflammation‐related genes in the liver and lung. (E, F) Heatmap analysis of fibrosis‐related genes in the liver and lung. (G) Venn diagram analysis based on RNA‐Seq data from liver and lung. (H, I) Gene ontology (GO) enrichment and KEGG pathway analysis based on common differentially expressed genes of Venn analysis.

To investigate the potential mechanisms underlying H1N1‐induced hepatic and pulmonary injury, RNA sequencing (RNA‐Seq) and subsequent bioinformatics analyses were performed on liver and lung tissues, respectively. Principal component analysis (PCA) revealed distinct gene expression profiles between the control and H1N1‐infected groups (Figure 6A). We then identified differentially expressed genes in the liver and lungs (log2 fold change ≥ 2 or ≤ −2, *p* adj ≤ 0.05) (Figure 6B). By reviewing the literature, we selected genes related to early fibrosis (or hepatic stellate cell activation) and inflammation (particularly those associated with H1N1 infection) and performed heatmap analysis (Figure 6C–F), confirming that viral invasion activates fibrosis and inflammatory responses in the liver and lungs. Next, we used a Venn diagram to identify 172 common genes between the two gene sets (Figure 6G), which we further analysed for enrichment. In the GO analysis, the enrichment of molecular function categories revealed abnormalities in antioxidant activity (Figure 6H), while KEGG enrichment analysis indicated significant anomalies in the ferroptosis pathway (Figure 6I).

**FIGURE 7:**
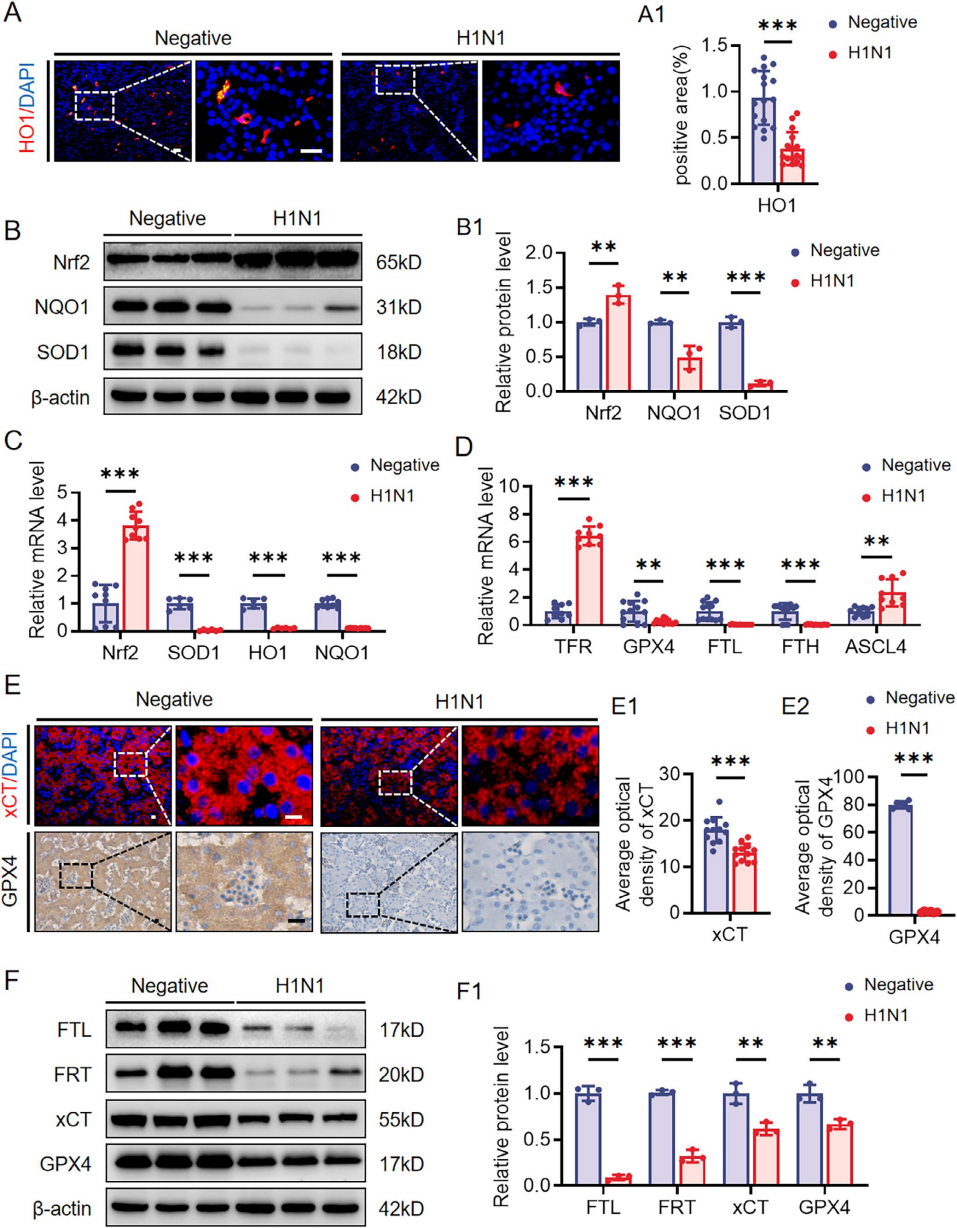
Assessment expression levels of oxidative stress and ferroptosis related proteins in the developmental liver. (A‐A1) HO1 immunofluorescence staining in the liver of E11.5 negative group and H1N1 virus‐infected chicken embryos (A), along with corresponding quantitative analysis (A1). (B‐B1) Western blot analysis showing the expression of Nrf2, NQO1, and SOD1 in the liver of the two groups (B), along with corresponding quantitative analysis (B1). (C, D) Quantitative PCR data showing the mRNA expression levels of Nrf2, SOD1, HO1, NQO1 (C), and TFR, GPX4, FTL, FTH, ASCL4 (D) in the liver. (E‐E2) XCT immunofluorescence staining and GPX4 immunohistochemical staining in the liver (E), along with corresponding quantitative analysis (E1‐E2). (F‐F1) Western blot analysis showing the expression of FTL, FRT, xCT, and GPX4 in the liver (F), along with corresponding quantitative analysis (F1). Scale bars = 20 μm in A, E. *n* = 3 (A–F). **p < 0.01, ***p < 0.001.

The occurrence of ferroptosis is closely related to changes in oxidative stress. Therefore, we selected several representative genes related to oxidative stress and ferroptosis for experimental validation in the liver and lungs. The results showed that, in the H1N1‐infected liver, the oxidative stress‐related proteins SOD1, HO1, and NQO1 were downregulated at both protein and RNA levels, while the oxidative stress‐related transcription factor Nrf2 was activated (Figures 7A–C and S7B‐C2). We then used an iron assay kit to measure the levels of total iron ions and Fe2+ and found that both were elevated in the H1N1‐infected group compared to the control, with an increased Fe2+ to total iron ion ratio, indicating potential Fe2+ overload leading to ferroptosis (Figure S6A,A1). We validated the mRNA levels of ferroptosis‐related genes CGTL, SLC40A1, VDAC3, STEAP3, NCOA4, TFRC, and SAT1 through RT‐qPCR, confirming the RNA‐Seq results (Figure S6B,B1). Finally, we examined the expression changes of other ferroptosis‐related genes at the RNA or protein level, and the results showed upregulation of TFR and ASCL4, along with downregulation of FRT, FTL, FTH, GPX4, and xCT (Figure 7D‐F1), suggesting the activation of the ferroptosis pathway. Notably, immunohistochemical results confirmed that ferroptosis markers GPX4 and xCT were primarily expressed in developing liver parenchymal cells (Figure 7E‐E2).

**FIGURE 8:**
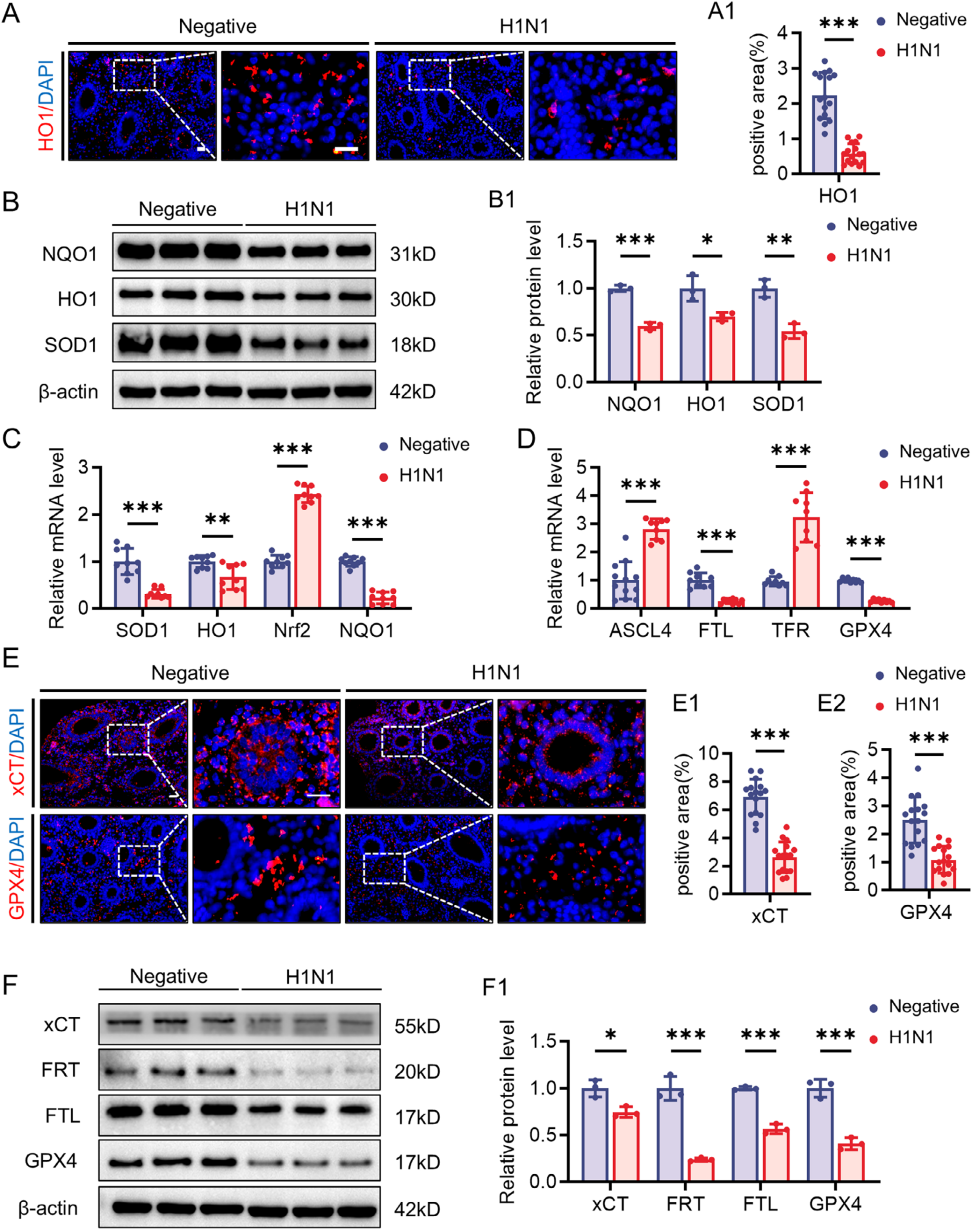
Assessment expression levels of oxidative stress and ferroptosis related proteins in the developmental lungs. (A‐A1) HO1 immunofluorescence staining in the lungs of E11.5 negative group and H1N1 virus‐infected group chicken embryos (A), along with the corresponding quantitative analysis (A1). (B‐B1) Western blot showing the expression of NQO1, HO1, and SOD1 in the lungs (B), along with the corresponding quantitative analysis (B1). (C, D) Quantitative PCR data showing the mRNA expression levels of SOD1, HO1, Nrf2, NQO1 (C) and ASCL4, FTL, TFR, GPX4 (D) in the lungs. E‐E2: XCT and GPX4 immunofluorescence staining in the lungs (E), along with the corresponding quantitative analysis (E1‐E2). (F‐F1) Western blot showing the expression of xCT, FRT, FTL, and GPX4 in the lungs (F), along with the corresponding quantitative analysis (F1). Scale bars = 20 μm in A, E. *n* = 3 (A–F). *p < 0.05, **p < 0.01, ***p < 0.001.

Using a similar approach, we further validated the impact of H1N1 virus on oxidative stress and ferroptosis in lung parenchymal cells. We also observed that oxidative stress‐related proteins SOD1, HO1, and NQO1 were downregulated at both protein and RNA levels in the lungs of H1N1‐infected groups, while the oxidative stress‐related transcription factor Nrf2 was activated (Figures 8A–C and S7A‐C2). Classic ferroptosis‐related proteins ASCL4, TFR, FRT, FTL, GPX4, and xCT exhibited similar changes as seen in the liver (Figure 8D‐F1). Additionally, immunofluorescence results indicated significant changes in GPX4 and xCT proteins in developing lung epithelial cells (Figure 8E‐E2).

To further verify the involvement of ferroptosis in H1N1‐induced organ injury and assess the feasibility of therapeutic intervention, a model comprising a control group, an H1N1 infection group, and an H1N1 plus Ferrostatin‐1 (Fer‐1) treatment group was established (Figure S8A). Results indicated that Fer‐1 did not affect H1N1 viral load (Figures S8B and S9A). However, PCR and Western blot analyses showed that treatment with Fer‐1 upregulated the expression of key ferroptosis‐regulating proteins, including FRT, xCT, and GPX4, while downregulating TFR in both liver and lung tissues (Figures S8C‐E, S9B‐D), suggesting that Fer‐1 effectively suppressed H1N1‐induced ferroptosis.

In addition, the mRNA expression levels of antioxidant‐related proteins HO1 and SOD1 were significantly increased following Fer‐1 treatment (Figures S8F,G and S9E,F). Levels of lipid peroxidation (LPO) and its metabolic product malondialdehyde (MDA) were decreased (Figures S8H and S9G), indicating reduced oxidative stress. Furthermore, ELISA and PCR analyses demonstrated that Fer‐1 partially reduced the expression of inflammatory cytokines IL‐1, IL‐6, and TNF‐α in the infection group (Figures S8I,J and S9H,I) and also suppressed the expression of the fibrosis marker gene ACTA2, which encodes α‐SMA (Figures S8K and S9J). These findings collectively confirm the pivotal role of ferroptosis in H1N1‐associated hepatic and pulmonary injury and highlight the potential protective effects of Ferrostatin‐1.

## 3 Discussion

The H1N1 virus has presented a substantial threat to humans. While individuals with mild infections often experience spontaneous recovery, the persistent emergence of novel recombinant strains heightens the risk of severe disease progression in those infected with H1N1 [23]. For certain immunocompromised populations, including pregnant women, the elderly, young children, and patients with underlying health conditions, H1N1 infection can result in more severe clinical outcomes, leading to multi‐organ failure and even death [24]. Current evidence clearly indicates that influenza virus infection during pregnancy is associated with adverse fetal outcomes. However, the precise mechanisms driving these effects remain a subject of ongoing debate. Proposed mechanisms include pregnancy termination resulting from decidual and placental infection due to viremia [25], fetal hypoxia caused by maternal or placental vascular inflammation impacting fetal blood supply [26], and direct placental and fetal injury due to maternal cytokine storms [27]. Although most studies have focused on the maternal and placental levels, substantial evidence indicates that the H1N1 virus can cross the placental barrier to directly infect the foetus. Autopsy reports of miscarried foetuses have also revealed viral accumulation in multiple organs [28, 29]. This suggests that the direct invasion of the virus into the foetus may be a significant factor contributing to adverse pregnancy outcomes. Due to the extensive proliferation and differentiation of stem cells involved in embryonic development, exposure to H1N1 infection early in life can lead to significant adverse outcomes [30]. Through a literature review combined with a meta‐analysis, we found that H1N1 virus infection during pregnancy is associated with a higher rate of severe illness and mortality. Additionally, the risk of adverse fetal outcomes, such as miscarriage and preterm birth, is also elevated (Figures 1 and S1).

To further investigate the underlying mechanisms, we established an in vitro infection model using chicken embryos. The chicken embryo model offers notable advantages, including a high degree of genomic homology with humans, ease of model establishment, and suitability for large‐scale experimental studies [22]. In previous studies, numerous researchers have extensively investigated the pathogenic mechanisms of various viruses using the chicken embryo model [21]. However, physiological differences between chicken embryos and mammals still exist. Future research may incorporate mammalian models, such as mice, for comparative studies to more comprehensively elucidate the mechanisms by which H1N1 virus infection affects fetal development.

Firstly, we selected the WSN strain of H1N1 virus to establish a disease model of H1N1 infection in chicken embryos. By examining the distribution of H1N1 virus across various organs, we found that the virus predominantly accumulates in the liver and lungs of the chicken embryos (Figures 2 and S2). Clinically, a substantial number of case reports and experimental studies have analysed the damage H1N1 virus inflicts on the lungs [7, 31]. Additionally, through a meta‐analysis, we discovered that H1N1 virus can cause liver enzyme abnormalities, indicating a risk of liver injury (Figure S3). The organ‐specific tropism of H1N1 virus for the liver and lungs may be related to the virus's affinity for endothelial cells. The H1N1 virus enters host cells through its surface hemagglutinin (HA) protein by recognising sialic acid (SA) receptors on the cell membrane (including sialic acid linked to galactose via α2,3 and α2,6 glycosidic bonds) [32]. Sialic acid is present at the outermost end of the glycan chains on most cells, particularly abundant in the thick glycocalyx of endothelial cells [33]. The liver is a highly vascularised organ, and its unique liver sinusoidal endothelial cells (LSECs) are abundant during embryonic development. This extensive vascular network plays a crucial role in supporting haematopoietic function and liver morphogenesis [34, 35, 36]. Maternal infection with influenza during pregnancy affects the haematopoietic function of offspring, which may be related to liver damage during the embryonic stage [37]. Similarly, pulmonary endothelial cells are critical for the establishment of the pulmonary vascular system, especially during the later stages of development. The abundance of endothelial cells provides the foundation for the formation of the highly vascularised alveolar structure, ensuring effective gas exchange in the lungs after birth [38, 39]. Studies have confirmed that influenza virus‐induced injury to the pulmonary endothelium is one of the leading causes of acute respiratory distress syndrome (ARDS) [40]. Additionally, we observed localised congestion on the surface of the chicken embryos, further indicating endothelial damage caused by H1N1 virus (Figure 2). In summary, the abundance of endothelial cells in the liver and lungs during embryonic development may be a potential cause of the organ‐specific effects exhibited by H1N1 virus.

After H1N1 virus enters the body, it is initially engulfed by resident immune cells in the tissues. These immune cells detect the virus through pattern recognition receptors (PRRs) that recognise viral RNA. This recognition triggers inflammatory responses and antiviral signalling pathways, leading to the secretion of a large number of pro‐inflammatory cytokines. As the inflammatory process progresses, natural killer (NK) cells and the adaptive immune response are also activated to combat the virus [41, 42]. In this study, H1N1 infection triggered a localised inflammatory response in the liver, accompanied by activation of the NF‐κB signalling pathway and upregulation of pro‐inflammatory cytokines including IL‐6, TNF‐α, and IL‐1β. Furthermore, the expression of T cell‐associated markers CD3 and CD8 was markedly elevated, suggesting a potential role for T cells in infection‐associated immune‐mediated injury (Figure 3). Previous studies have demonstrated that mast cells, as key effector cells in early immune responses, play a critical role in cytokine storm formation and tissue damage induced by H1N1 infection. Caiyun Huo et al. reported that melatonin could alleviate H1N1‐induced lung injury in mice by suppressing mast cell activation and reducing pro‐inflammatory cytokine release [43]. The findings of our study are consistent with this report. In addition, B cells are essential components of the adaptive immune response. Jens Wrammert et al. employed cloning techniques to isolate highly effective human monoclonal antibodies, confirming the pivotal role of B cells in clearing influenza virus infections [43]. Consistent with these observations, we detected upregulation of B cell marker expression, indicating activation of B cell‐related immune responses following H1N1 infection. Notably, although numerous studies have suggested that macrophage activation contributes significantly to organ injury associated with influenza virus infection [44], no apparent upregulation of macrophage‐associated markers was observed in this study. Moreover, the expression of the M1 macrophage marker NOS2 was downregulated, implying that classical M1 polarisation of macrophages may not have occurred in this chicken embryo model, or that macrophages may have shifted towards an anti‐inflammatory M2 phenotype. Due to the limitations of the chicken embryo model, for which many antibodies do not work, more animal models should be used in the future to elucidate the corresponding mechanisms at the protein level.

The sustained infiltration of immune cells and inflammatory signalling can induce various forms of programmed cell death [45]. Experimental results show reduced proliferation of hepatocytes and lung parenchymal cells, alongside the upregulation of molecules related to programmed cell death, suggesting the presence of an active cell death process (Figures 4 and 5). To investigate the specific mechanisms underlying H1N1 virus‐induced parenchymal cell death in the liver and lungs, we performed RNA sequencing analysis on tissue samples from these organs and conducted enrichment analysis of the common differentially expressed genes. The results demonstrated that both organs exhibited abnormalities in antioxidant activity, and the ferroptosis pathway was significantly involved (Figure 6). Further experimental findings indicated a downregulation of antioxidant molecules HO1 and SOD1 in the liver and lungs, alongside an upregulation of the oxidative stress‐related molecule Nrf2, suggesting that H1N1 virus induces oxidative stress in these organs. Notably, this paradoxical pattern may reflect a compensatory upregulation of Nrf2 in response to excessive oxidative stress, while persistent redox imbalance and inflammatory conditions impair the downstream antioxidant defence, leading to a paradoxical decrease in HO‐1, NQO1, and SOD1 levels. Similar phenomena have been reported in other viral infections [46]. The changes in cellular oxidative stress not only form a vicious cycle with inflammation, driving immune cell activation and cytokine release [47], but also promote cell death through the excessive accumulation of peroxides [48]. Unlike other forms of cell death, ferroptosis is a form of iron‐dependent cell death induced by the accumulation of lipid peroxides, and it is closely associated with the imbalance of the cellular antioxidant system [49]. The experimental results of this study further confirm that H1N1 infection induces typical ferroptosis characteristics in the liver and lungs, including abnormal iron transport, peroxide accumulation, and reduced antioxidant capacity (Figures 7, 8, and S6). Ferroptosis has been observed in a variety of viral infections, including influenza virus, HIV, SARS‐CoV‐2, and enteroviruses, indicating that ferroptosis may be an important mechanism of virus‐induced cell death [50]. Specifically, for H1N1 virus, studies have shown that it can trigger ferroptosis in A549 cells and nasal epithelial cells, and this process is closely related to the immune response [51, 52]. In our study, we did not observe direct viral distribution in hepatocytes and pulmonary parenchymal cells, suggesting that the virus indirectly promotes ferroptosis in these cells by altering oxidative stress and iron metabolism in the local environment through the induction of immune responses. This finding provides new insights into the pathogenic mechanisms of H1N1 virus and offers potential targets for future therapeutic strategies aimed at regulating ferroptosis.

Persistent immune cell infiltration and pro‐inflammatory signalling can lead to an imbalance in oxidative stress and programmed cell death, which in turn drives an imbalance in the organ tissue damage and repair processes, ultimately inducing pathological fibrosis [11]. This study found that H1N1 virus infection not only induces significant inflammatory responses and ferroptosis in the liver and lungs but also is accompanied by the early onset of a fibrotic phenotype. Specifically, the upregulation of α‐SMA expression and Sirius Red staining both indicate a marked increase in the number of fibroblasts and collagen deposition in these two organs. Gene expression analysis further confirmed that H1N1 infection activates multiple signalling pathways related to fibroblast activation and fibrosis in both the liver and lungs (Figures 4, 5, 6). Notably, hepatic stellate cells (HSCs) are the main source of fibroblasts in the liver and play a decisive role in the occurrence and progression of liver fibrosis [53]. Through immunofluorescence co‐localisation experiments, we found that the H1N1 virus can directly invade HSCs, and as the viral load increases, the number of activated HSCs also increases (Figure S5). This result suggests that H1N1 virus may regulate liver fibrosis by directly infecting HSCs. Previous studies have confirmed that hepatitis B virus, human immunodeficiency virus, and cytomegalovirus can infect HSCs, promoting their proliferation and the expression of COL1A1 [54, 55, 56]. Our findings further suggest that H1N1 virus possesses similar capabilities. However, the increase in viral load is usually accompanied by enhanced inflammatory responses, and the release of pro‐inflammatory factors can also promote HSC activation [57]. Therefore, the direct effects of H1N1 on HSCs and the underlying mechanisms require further in‐depth research.

To further validate the role of ferroptosis in the organ damage, inflammation, and fibrosis progression associated with H1N1 viral infection, we established a Ferrostatin‐1 (Fer‐1) intervention model. The results indicated that although Fer‐1 did not affect the viral replication level, it effectively inhibited the abnormal expression of ferroptosis‐related molecules in the liver and lungs, reduced levels of LPO and MDA, and restored antioxidant capacity. Additionally, Fer‐1 partially decreased the levels of pro‐inflammatory cytokines such as IL‐1, IL‐6, and TNF‐α, and suppressed the expression of the fibrosis marker gene ACTA2, suggesting that ferroptosis plays a crucial role in the fibrotic pathological process associated with H1N1 infection (Figures S8 and S9). These findings indicate that ferroptosis not only mediates oxidative stress and cell death induced by H1N1 infection, but also contributes to inflammation amplification and fibrosis formation, ultimately exacerbating organ damage.

**FIGURE 9:**
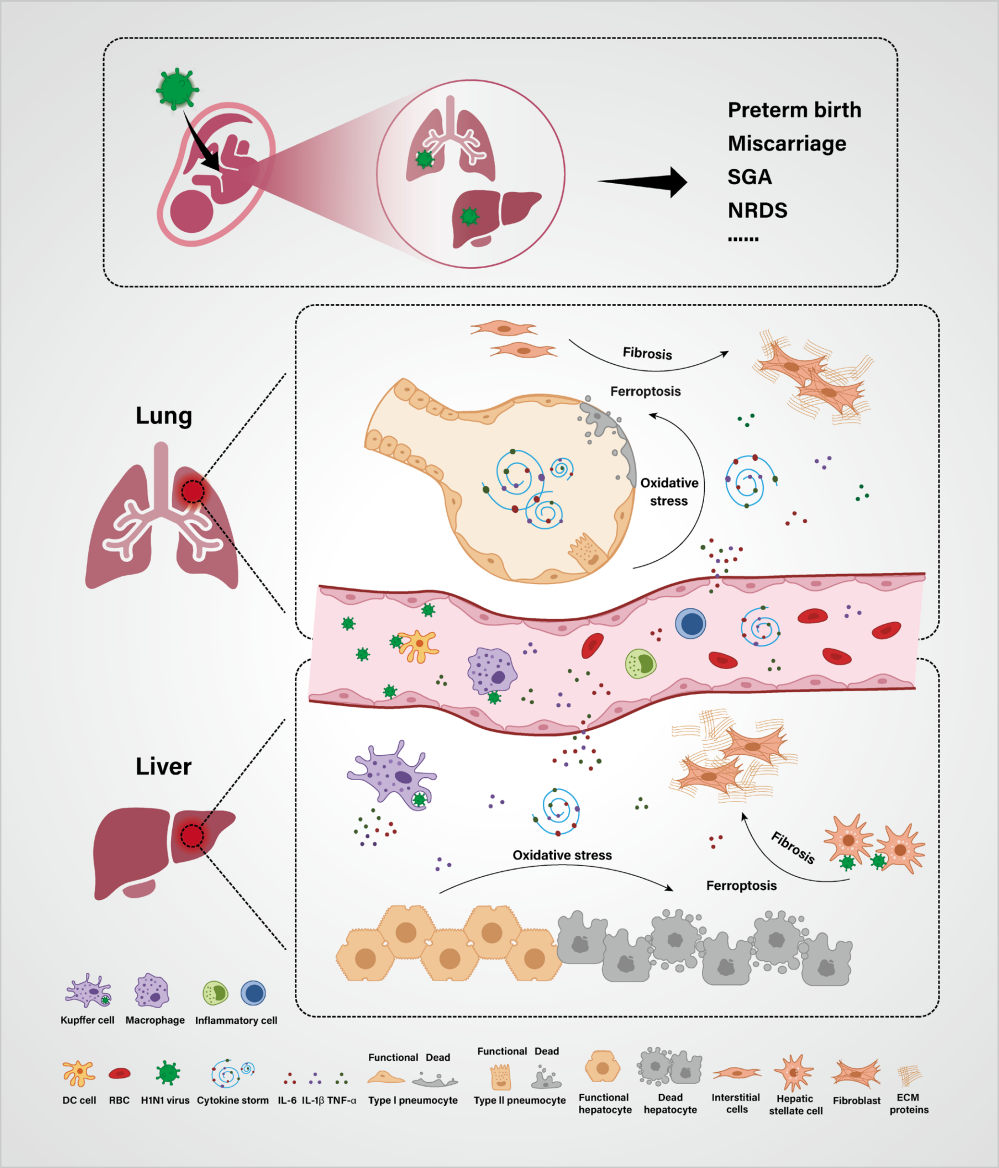
A proposed model to depict H1N1 influenza A virus induces fetal liver and lung injury through ferroptosis.

Overall, this study found that the H1N1 virus exhibits a distinct organotropism in the developing embryo, with particular susceptibility to highly vascularised organs such as the liver and lungs. Viral infection activates immune cells in the blood and stromal cells within the organs, inducing the release of a large number of pro‐inflammatory cytokines, which in turn trigger localised oxidative stress responses. The sustained oxidative stress disrupts intracellular iron homeostasis, leading to the accumulation of lipid peroxides and inducing ferroptosis in the parenchymal cells of the liver and lungs. This cell death subsequently drives tissue repair processes, activating fibroblasts and promoting extracellular matrix production, ultimately leading to early fibrotic changes. These findings reveal the critical mechanisms underlying embryonic developmental abnormalities induced by H1N1 virus infection and provide new theoretical insights for the prevention and treatment of influenza virus‐related multi‐organ damage and adverse pregnancy outcomes (Figure 9).

## 4 Materials and Methods

### 4.1 Meta‐Analysis

The systematic search was conducted to identify studies published on or before October 26, 2024, using PubMed and the China National Knowledge Infrastructure (CNKI) databases with specific search terms. Eligible studies were included in the meta‐analysis based on the following criteria: (1) Case–control studies categorised by disease severity (e.g., severe vs. non‐severe, ICU vs. non‐ICU, or deceased vs. surviving patients). (2) Studies reporting symptoms, medical history, laboratory findings, and outcomes in H1N1 patients. The exclusion criteria were: (1) Animal studies. (2) Review articles, letters, case reports, abstracts, and other non‐primary research. (3) Studies with incomplete data or where full text was not accessible. The search terms used for retrieval are provided in Table S1, and the basic information of the included studies is listed in Tables S2 and S3. Forest plots and corresponding sensitivity analyses are shown in Figures 1,S1, and S3, and publication bias assessment results are presented in Table S4.

### 4.2 Avian Embryo and Experimental Treatment

The stock of H1N1 influenza virus strain A/WSN/33 (WSN) was gifted by Prof. Shuwen Liu (Southern Medical University) and was stored at −80°C. Fertilised chicken eggs were obtained from the poultry farm of South China Agricultural University. These eggs were incubated at 38°C with 70% humidity in a humidity‐controlled incubator (Yiheng Instrument, Shanghai, China). On day 8.5 of incubation, the eggs were randomly divided into three groups: control group, H1N1 virus group, and Fer‐1 intervention group. The H1N1 virus group was inoculated by injecting 200 μL of the viral suspension (10,000 TCID₅₀) into the air chamber, while the control group received an equal volume of sterile PBS. The Fer‐1 intervention group was simultaneously injected with 200 μL of H1N1 viral suspension (10,000 TCID₅₀) and 1 μM Ferrostatin‐1 (Fer‐1) mixed solution. The working concentration of Fer‐1 was determined based on previous literature (Reference) and adjusted according to the volume of the chicken embryo and the viral inoculation dose. For the dose‐dependent experiment (Figure S5), three different viral titers (1000, 5000, and 10,000 TCID₅₀) were used. All other experiments in this study were performed using a consistent viral titre of 10,000 TCID₅₀. The surviving embryos were photographed using a stereomicroscope (Olympus MVX10, Japan) and subsequently fixed in 4% paraformaldehyde for morphological and gene expression analysis. Only viable embryos (*N* ≥ 30) were included for further study to ensure data reliability.

### 4.3 Immunofluorescence and Immunohistochemical Staining

The tissue sections were deparaffinised in xylene, rehydrated, and then subjected to antigen retrieval by heating in citrate buffer (pH 6.0). To block endogenous peroxidase activity, the sections were treated with 3% hydrogen peroxide for 10 min. Non‐specific immune reactions were minimised by incubating the sections with 5% inactivated goat serum in PBS at room temperature for 30 min. After washing with PBS, the sections were incubated overnight at 4°C with the primary antibody on a shaker. The next day, after extensive washing, the sections were incubated at room temperature with HRP‐conjugated goat anti‐rabbit IgG secondary antibody (1:400, EarthOx, USA) for 3 h in the dark. Detection was performed using DAB (Maixin, China) as the chromogen, followed by counterstaining with haematoxylin. For immunofluorescence staining, the sections were incubated overnight with the primary antibody at 4°C, followed by a 2‐h incubation at room temperature with Alexa Fluor 555 or 488 conjugated secondary antibody (1:1000, Invitrogen, USA) on a shaker. All immunofluorescence sections were counterstained with DAPI (1:1000, Invitrogen, USA) at room temperature for 1 h. Immunohistochemistry was performed according to the manufacturer's protocol (Elabscience Biotechnology Co. Ltd., Wuhan, China). Detailed information about the antibodies used is provided in Table S6.

### 4.4 Histomorphology

Continuous 5 μm sections were used for haematoxylin and eosin (HE) staining and Sirius Red staining for morphological analysis. Images of the stained sections were captured using fluorescence microscopes (Olympus IX51, Leica DM 4000B), and Image Pro‐Plus 7.0 software was used to quantify fibrotic lesions. For each embryo, at least three randomly selected images were analysed to ensure accurate quantification.

### 4.5 Western Blot and qPCR Analysis

Proteins were extracted using a Radio‐Immunoprecipitation Assay (RIPA) buffer (Sigma, USA), supplemented with protease and phosphatase inhibitors. The total protein concentration was determined using the Bicinchoninic Acid (BCA) Assay Kit (BCA01, DingGuo BioTECH, China). Equal amounts of protein from each sample were separated by SDS‐PAGE and subsequently transferred onto polyvinylidene fluoride (PVDF) membranes (Bio‐Rad). The membranes were blocked with 5% Difco skim milk (BD) and incubated sequentially with primary and secondary antibodies. Signal detection was performed using the SuperSignal West Femto Chemiluminescent Substrate (ThermoFisher, Rockford, USA) and visualised with the Gel Doc XR+ system (Bio‐Rad, California, USA). The experiment was repeated independently at least three times, ensuring reproducibility. Detailed information on the antibodies used is provided in Table S5. The qPCR analysis was performed as previously described [58]. The qPCR primers used in this study are listed in Table S7. At least three independent experiments were performed for qPCR analysis.

### 4.6 Iron Assay Kits

The ferrous ion content was assessed using the #E‐BC‐K773‐M kit, while total iron levels were determined using the #E‐BC‐K772‐M kit, both provided by Elabscience (Wuhan, China).

### 4.7 ELISA Kits

The levels of interleukin‐1β (IL‐1β), tumour necrosis factor‐α (TNF‐α), and interleukin‐6 (IL‐6) in the liver and lung were analysed using ELISA kits (IL‐1β: JL21623; TNF‐α: JL21706; IL‐6: JL21628; Jianglai Biotechnology Co. Ltd., Shanghai, China) following the manufacturer's instructions.

### 4.8 Biochemical Assay Kit

The levels of malondialdehyde (MDA) and lipid peroxidation (LPO) were evaluated using commercial assay kits (MDA: bc0020; LPO: bc5240; Solarbio, China) according to the manufacturer's protocols.

### 4.9 RNA Sequencing (RNA‐Seq) Transcript Profiling

RNA extraction, library construction, and sequencing were conducted by Beijing Geneplus Technology Co. Ltd. Gene Ontology (GO) enrichment and Kyoto Encyclopedia of Genes and Genomes (KEGG) pathway analyses were performed using the www.bioinformatics.com.cn platform, with bubble plots generated to visualise the results. All RNA‐seq data have been deposited in the NCBI GenBank database under the Sequence Read Archive (SRA) with accession number PRJNA1189873.

### 4.10 Data Analysis

Morphological or immunofluorescence analyses were conducted using Image Pro‐Plus 7.0 software. Statistical analysis of all experimental data was performed with the SPSS 26.0 statistical package. Data are presented as mean ± SD. Group means were compared using a t‐test to evaluate differences, with statistical significance set at *p* < 0.05. For quantitative analysis, at least three sections or samples were selected from each group for staining. All experiments and analyses were repeated at least three times, and representative images are shown. Statistical values and sample sizes for independent experiments are provided in Tables S8–S20.

## Author Contributions

Y.J. and Y.S. performed most of the experiments and collected samples; Z.L., Y.L., and J.P. analysed the experimental data, F.G., X.Y., Q.Z., and G.W. designed the study, Q.‐Y.S. and Z.L. and X.‐H.O. read the full text and provided guidance on ideas, Y.J., Q.Z., and G.W. wrote the manuscript. All authors read and approved the final manuscript.

## Ethics Statement

All procedures involving the propagation of influenza virus in embryonated chicken eggs were conducted in accordance with institutional and national guidelines for the care and use of laboratory animals. Specific‐pathogen‐free (SPF) chicken embryos at embryonic day 11.5 (E11.5) were used in this study. According to the guidelines of the Institutional Animal Care and Use Committee (IACUC) and prevailing national regulations, chicken embryos at less than 13 days of development are not classified as live animals requiring ethical review. All experimental procedures were performed by trained personnel using aseptic techniques within Biosafety Level 2 (BSL‐2) laboratories, in compliance with approved biosafety protocols and ethical disposal procedures.

## Conflicts of Interest

The authors declare no conflicts of interest.

## Supporting information


**Data S1:** Supporting Information.

**Data S2:** Supporting Information.

## Data Availability

The data that support the findings of this study are available from the corresponding author upon reasonable request.
