# Supplementary material for: Influenza A Virus (H1N1) Infection Induces Ferroptosis to Promote Developmental Injury in Fetal Tissues
Source: Cell Prolif. 2025 Aug 26;59(3):e70117. doi: 10.1111/cpr.70117 (PMC12961522; doi:10.1111/cpr.70117)
Supplement: Supplementary file 1 — Data S1: Supporting Information. [file CPR-59-e70117-s002.docx]

**Supplementary Figure**


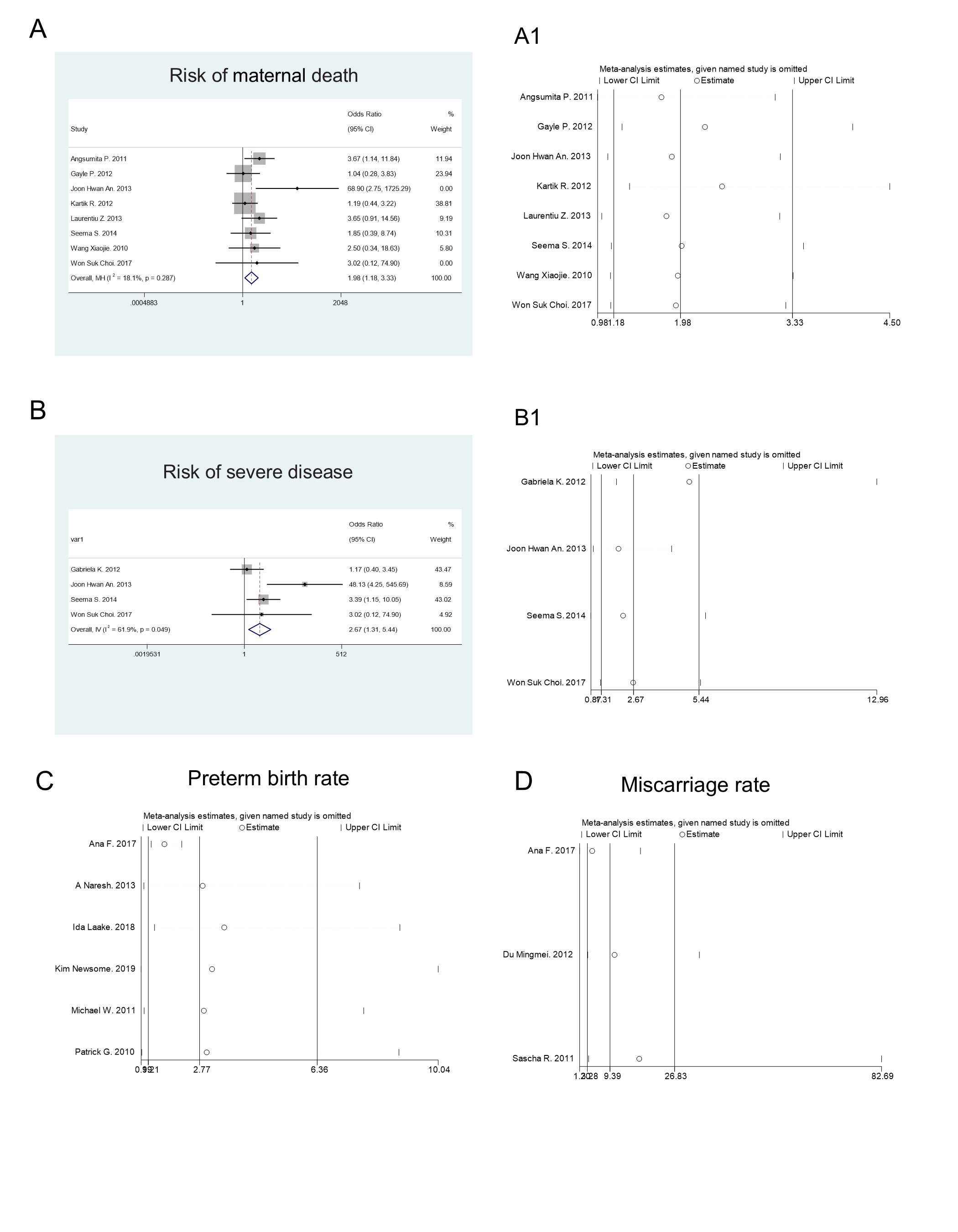
***Supplementary Figure 1. Meta-analysis for the correlation between H1N1 virus infection and maternal death, severe disease, preterm birth rate, and miscarriage rate.***

**A-B:** Forest plots illustrating the effect of H1N1 virus infection on maternal death (A) and any severe outcome in pregnant women (B), along with their respective sensitivity analyses. **C-D:** Sensitivity analyses evaluating the association between H1N1 virus infection and the rates of preterm birth (C) and miscarriage (D).

**
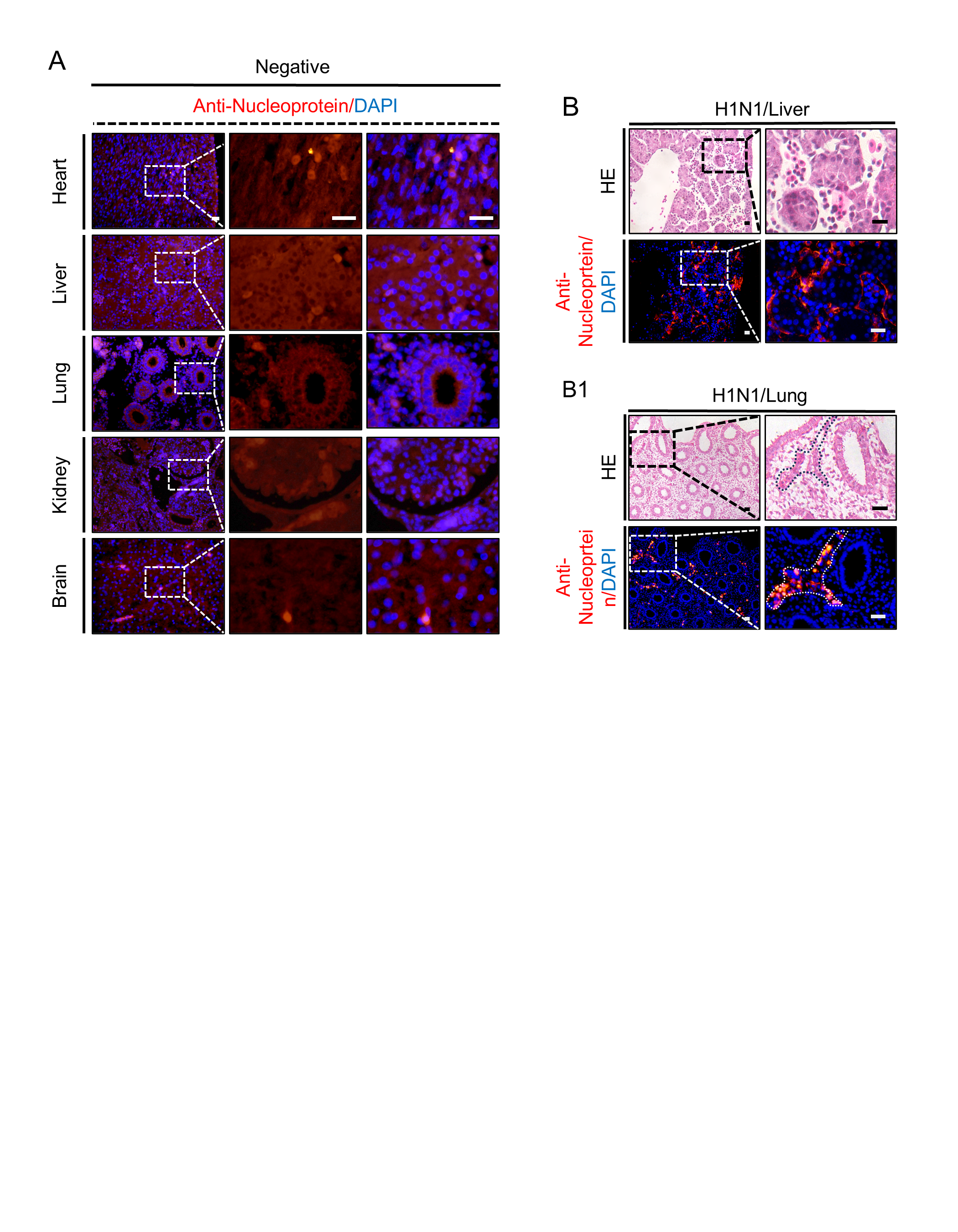
**

***Supplementary Figure 2. Anti-Nucleoprotein immunofluorescence staining in the negative chicken embryo.***

**A:** Representative cross-sectional immunofluorescence images showing anti-Nucleoprotein staining in the heart, liver, lungs, kidneys, and brain of E11.5 negative chicken embryos. **B-B1:** Representative cross-sectional images of HE staining and corresponding anti-Nucleoprotein immunofluorescence staining in the liver (B) and lungs (B1) of E11.5 H1N1 virus-infected chicken embryos. Scale bars = 20μm in A-B.


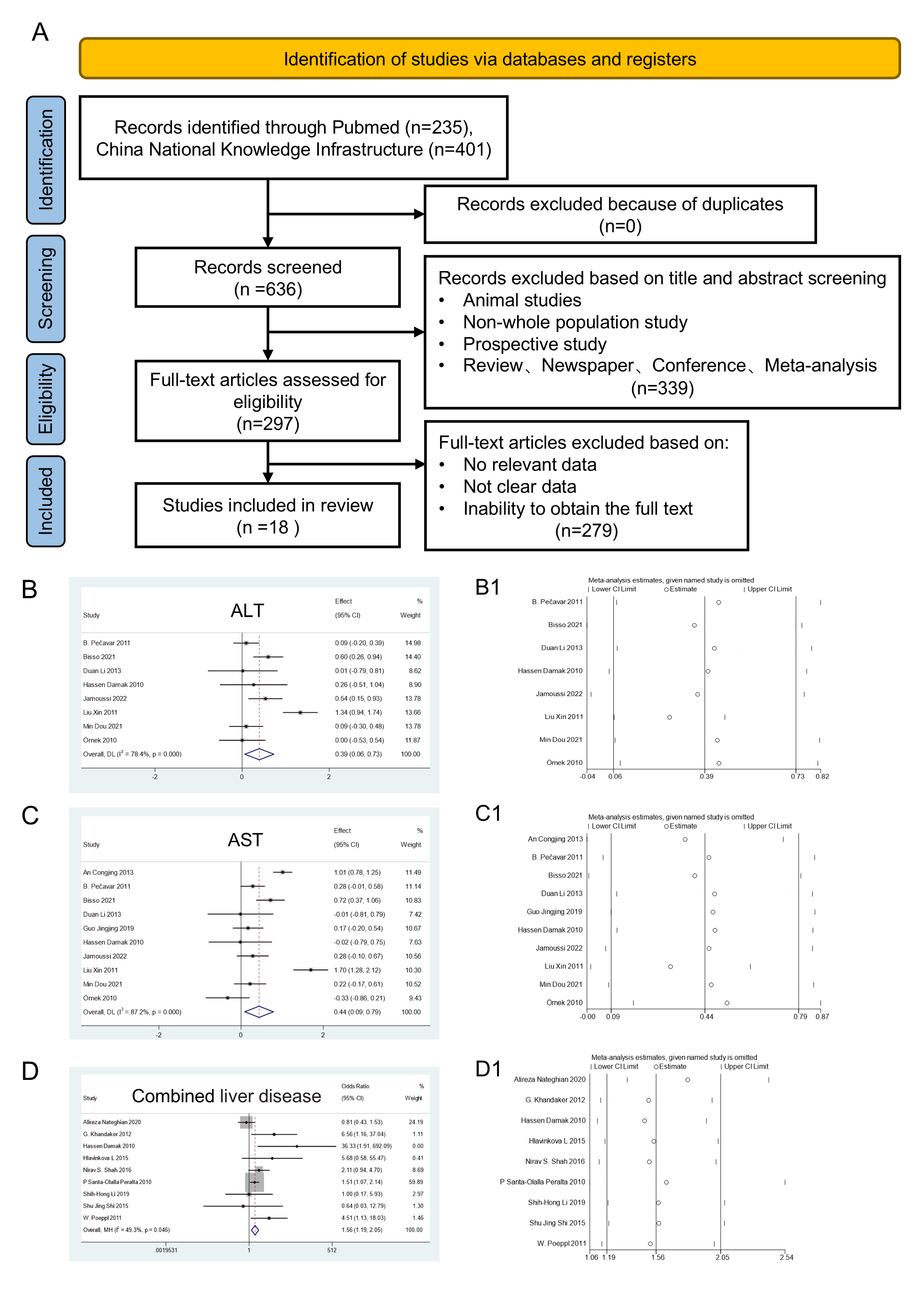


***Supplementary Figure 3. Meta-analysis for the correlation between H1N1 infection and Liver damage.***

**A:** Flow chart of systematic literature search in PubMed and CNKI with the following search terms: English search term: ("Risk Factors"[Mesh]) AND ((clinical features) OR (clinical characteristics) OR (clinical outcomes)) AND ("Influenza A Virus, H1N1 Subtype"[Mesh]). The search period ranges from January 2007 to October 2024. **B-D:** Forest plots illustrating the relationship between liver function levels (AST, ALT, and Combined liver disease) and the severity of H1N1 virus infection, along with their respective sensitivity analyses. ALT: alanine aminotransferase; AST: aspartate aminotransferase.


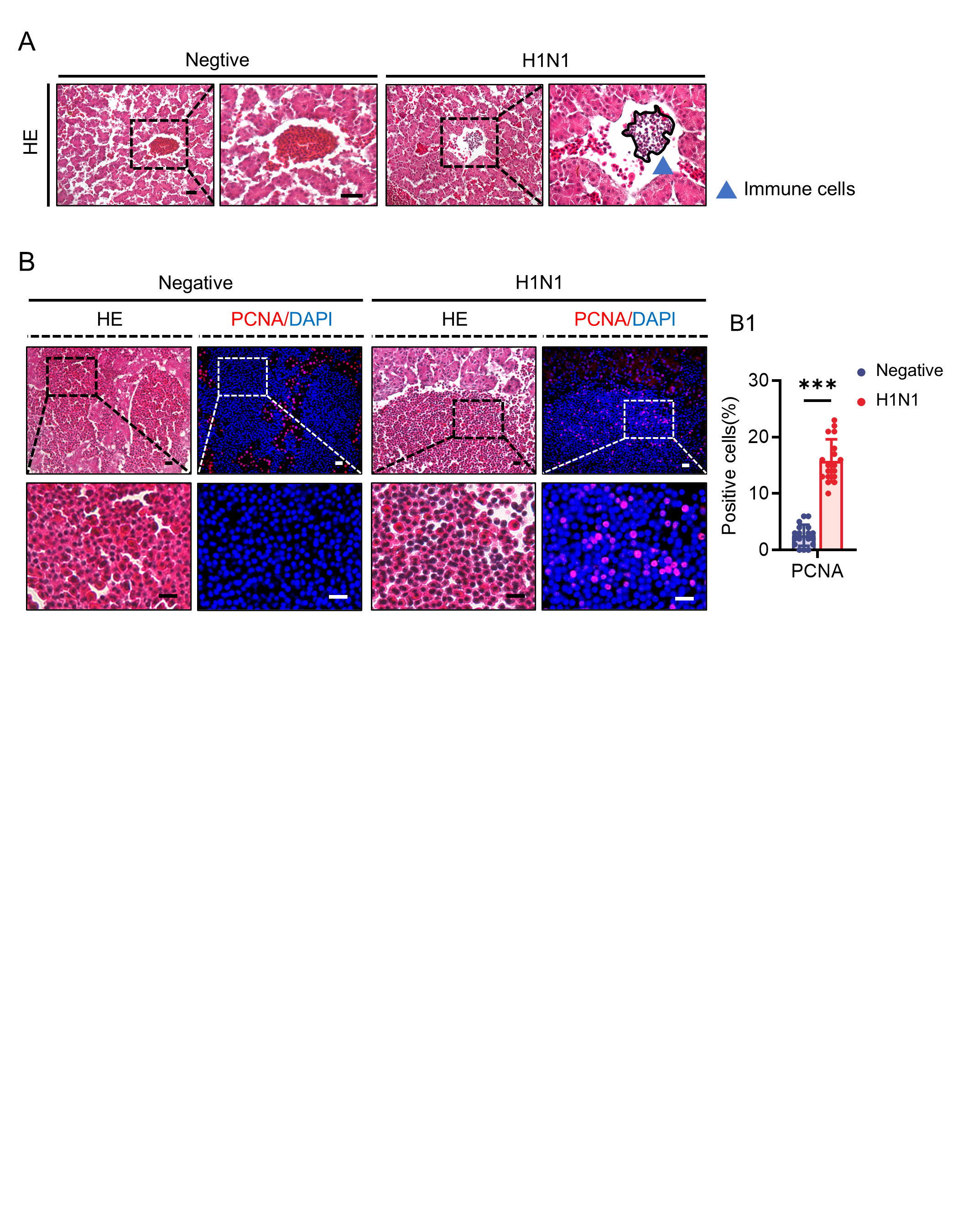


***Supplementary Figure 4. Assessment of cell changes in the blood vessel of developmental liver after H1N1 virus infection.***

**A:** HE staining of the central vein of liver in chicken embryos from the E11.5 negative group and the H1N1 virus-infected group, with immune cell aggregation indicated by blue triangles. **B-B1:** PCNA immunofluorescence staining of blood cells in the liver of chicken embryos from the E11.5 negative group and the H1N1 virus-infected group (B), along with corresponding HE staining in the same regions. The bar charts showing the comparisons of PCNA-positive cells in total DAPI-labeled cells (B1). Scale bars = 20μm in A, B. n = 3 (B). *** P < 0.001.


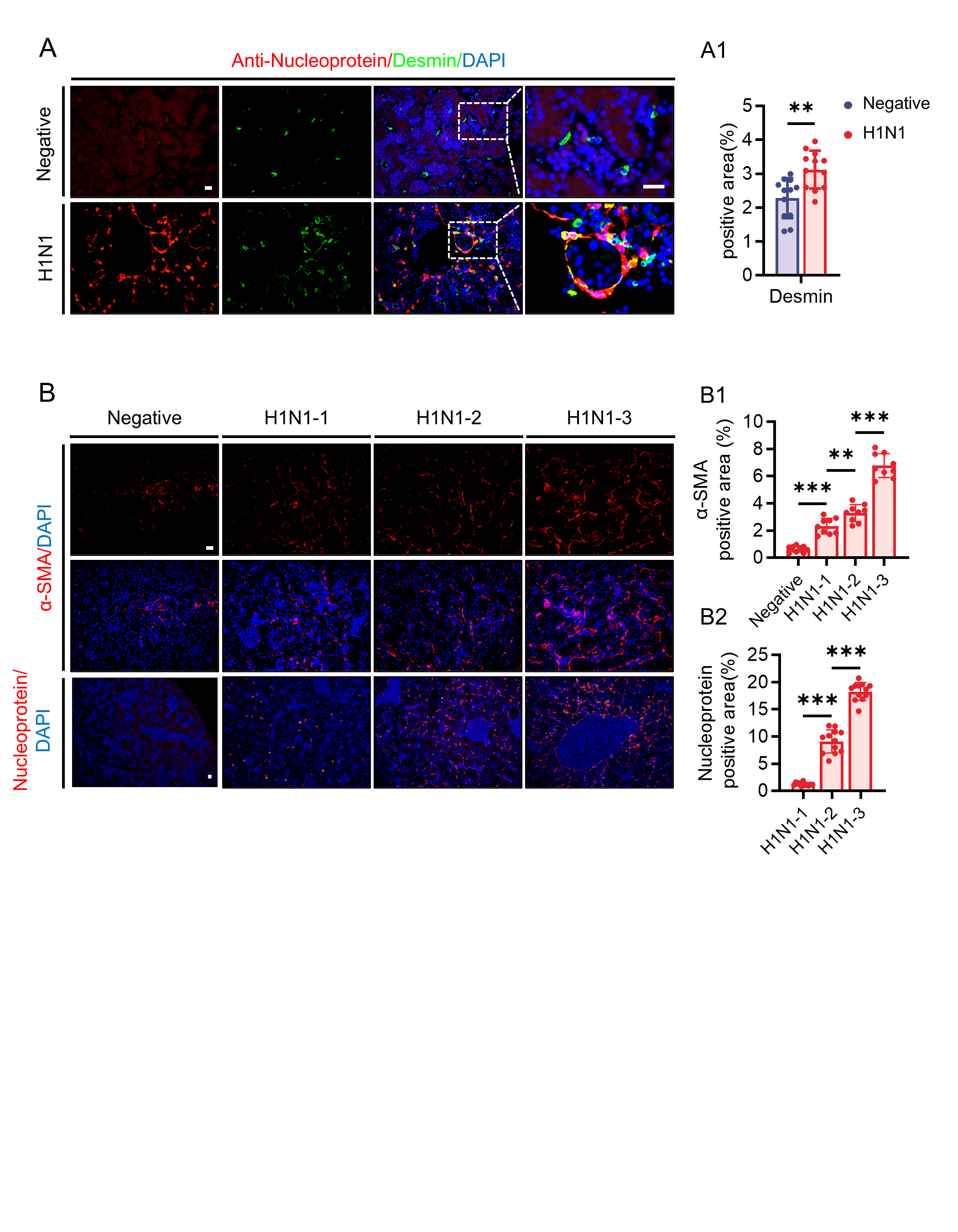


***Supplementary Figure 5. Detection of hepatic stellate cell infection by H1N1 virus.***

**A-A1:** Immunofluorescence double staining with Desmin and anti-Nucleoprotein in the liver of chicken embryos from the E11.5 H1N1 virus-infected group. The bar charts showing the comparisons of Desmin positive areas in total areas (A1). **B-B2:** Immunofluorescence staining of α-SMA and anti-Nucleoprotein in the liver of E11.5 chicken embryos in the negative group and H1N1 virus-infected group (B). H1N1-1, H1N1-2, and H1N1-3 were processed by injecting 200 µL of diluted PBS containing 1,000, 5,000, and 10,000 TCID₅₀ of seed virus into the air chamber, respectively. Quantitative analysis is shown in B1-B2. Scale bars = 20μm in A, B. n = 3 (A, B). ** P < 0.01, *** P < 0.001.

**
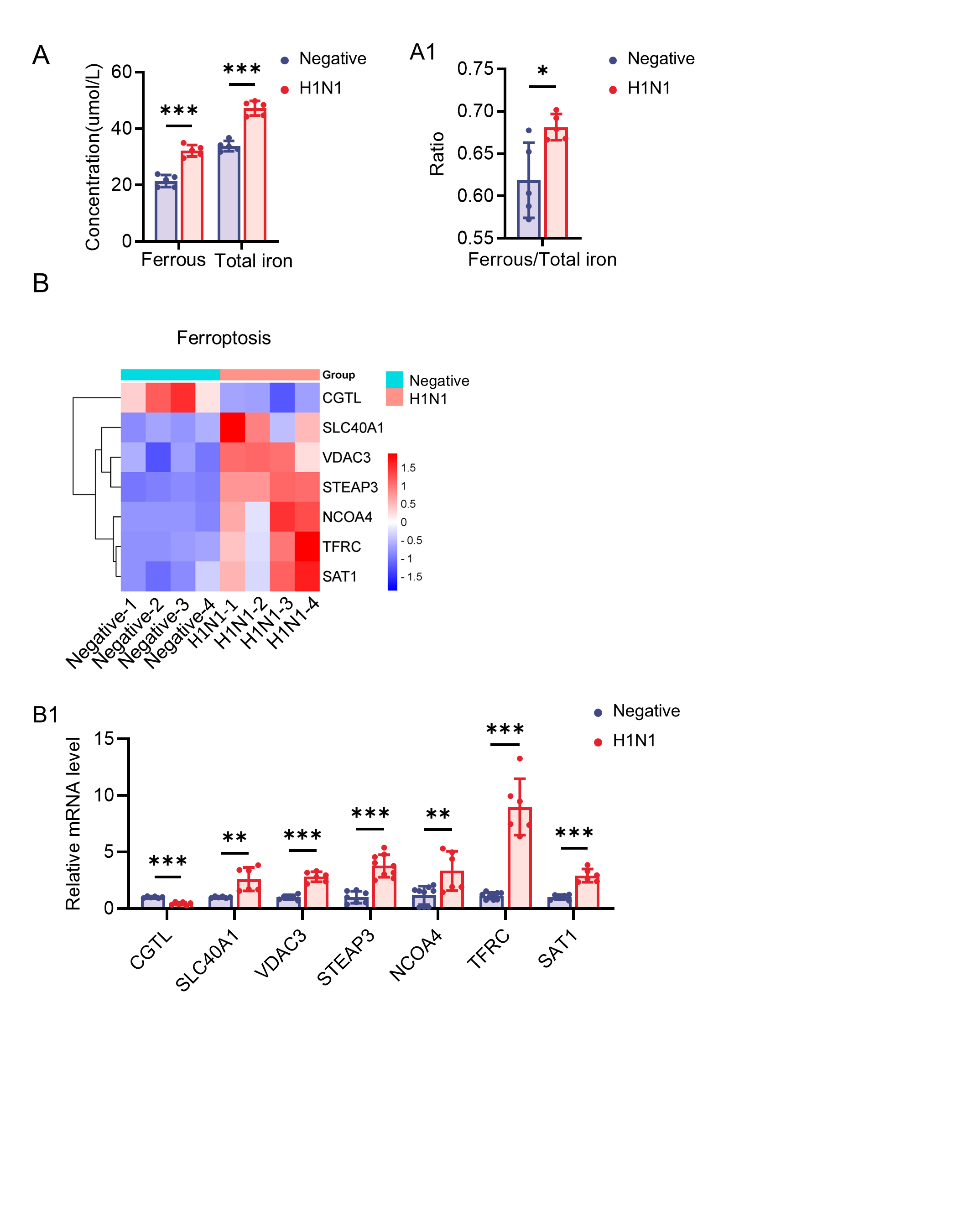
**

***Supplementary Figure 6. Assessment of Fe^2+^, total iron ion and ferroptosis related differentially expressed genes (DEGs)*** ***in the developmental liver.***

**A-A1:** Detection of differences in Fe^2+^ and total iron ion content between the negative group and the H1N1 virus-infected group, and the ratio of Fe^2+^ to total iron content. **B-B1**: Heatmap of ferroptosis-related DEGs identified through mRNA-seq in the liver of chicken embryos infected with H1N1 virus (B), along with quantitative PCR validation (B1). n = 3 (A, B1). * P < 0.05, ** P < 0.01, *** P < 0.001.


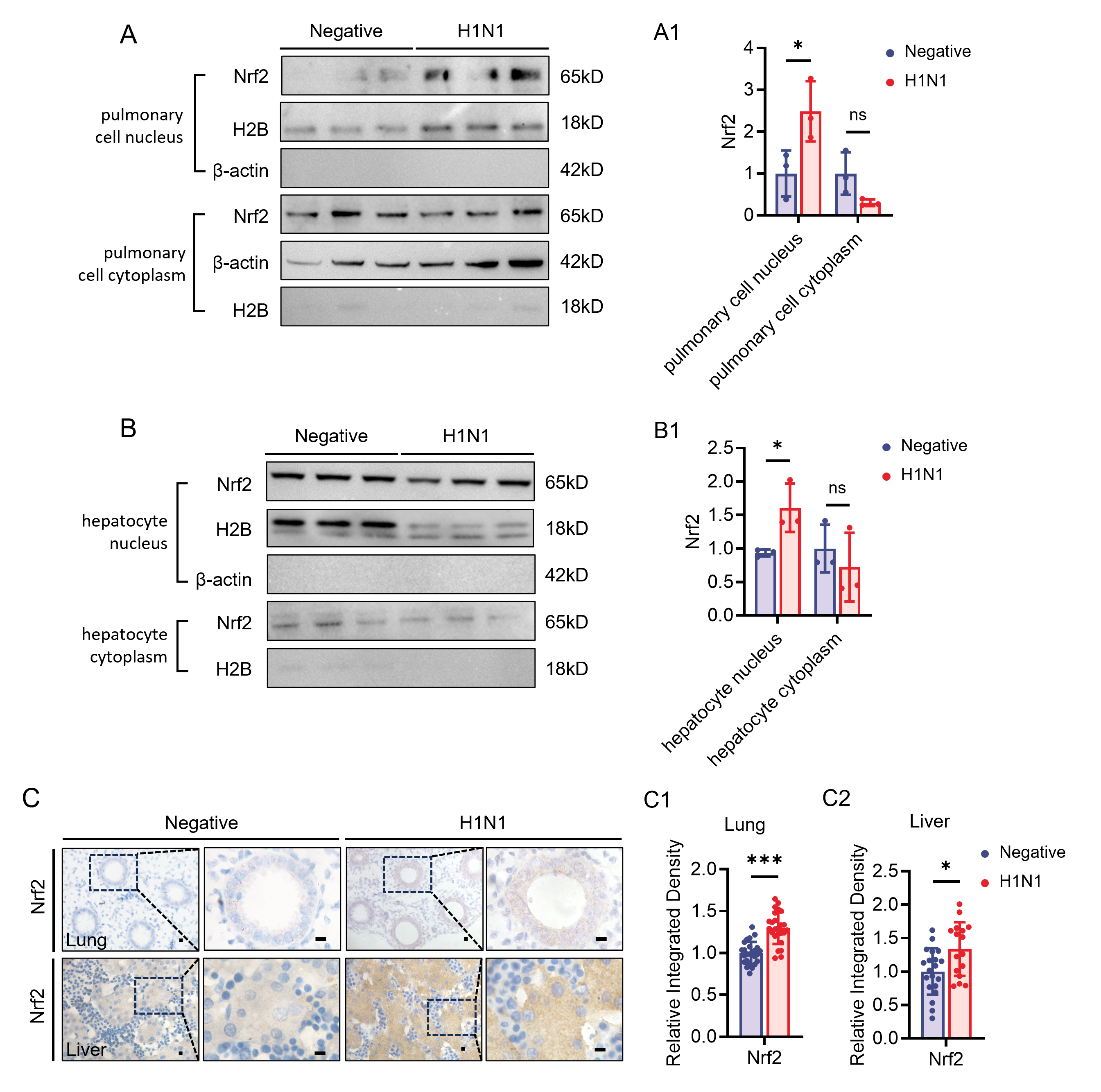


***Supplementary Figure 7. Analysis of Nrf2 Expression Levels and Nuclear Translocation in the Liver and Lungs After H1N1 Virus Infection.***

**A-A1:** Western blot analysis showing the expression levels of Nrf2 in both the nuclear and cytoplasmic fractions of pulmonary cells (A), and the corresponding quantitative analyses (A1). **B-B1:** Western blot analysis showing the expression levels of Nrf2 in both the nuclear and cytoplasmic fractions of liver cells (B), and the corresponding quantitative analyses (B1). The β-actin loading control for the hepatocyte cytoplasmic fraction is provided in Figure 3C. **C-C2:** Immunohistochemical staining showing Nrf2 expression in the liver and lung of chicken embryos (C), and the relative integrated density of nuclear Nrf2 (C1-C2). Scale bars = 20μm in C. n = 3 (A-C). * P < 0.05, *** P < 0.001, ns: not significant.**
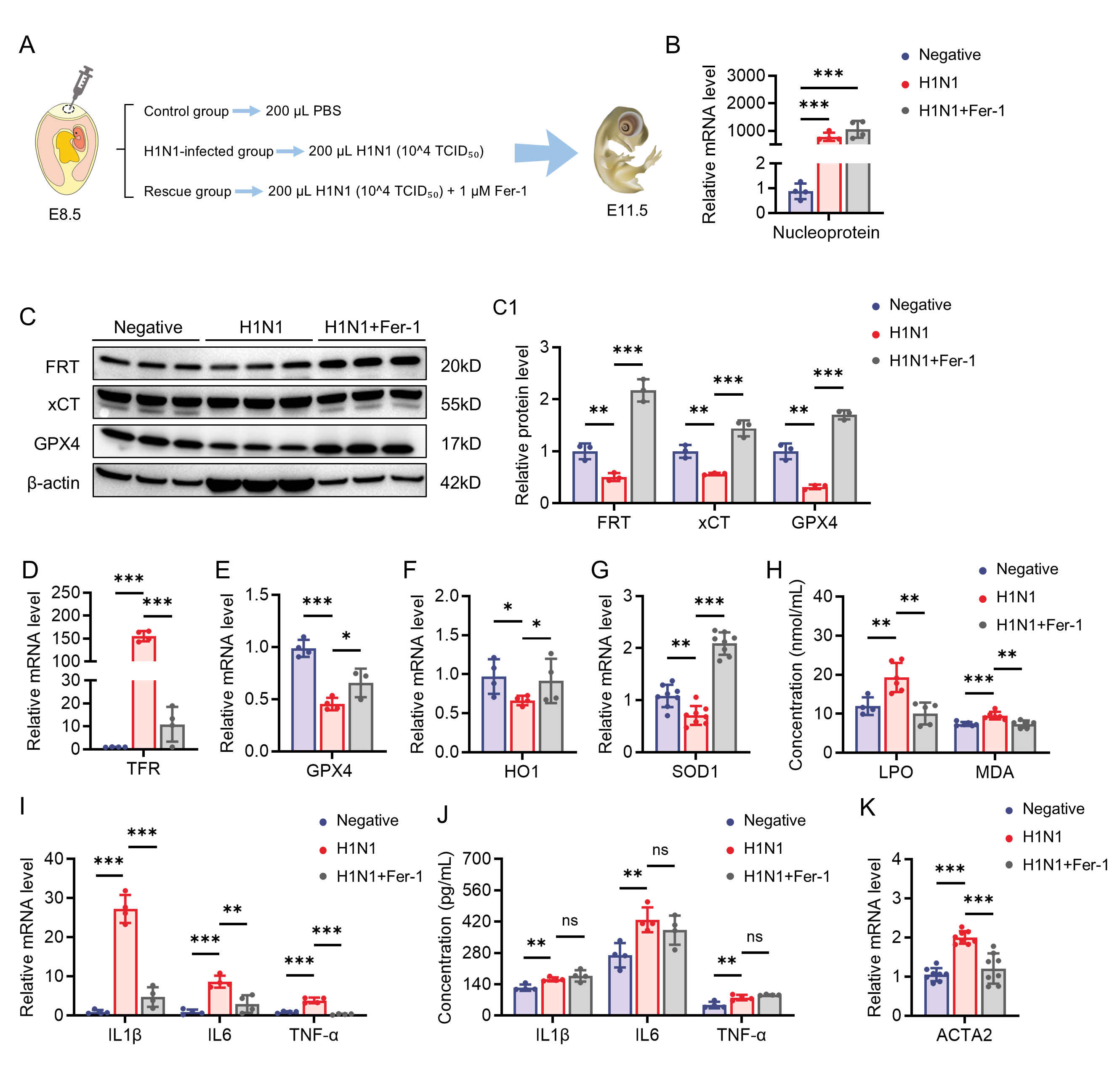
**

***Supplementary Figure 8. Evaluation of the effects of Fer-1 treatment on liver injury in chicken embryos following H1N1 virus infection.***

**A:** Schematic diagram illustrating the establishment of control, H1N1-infected, and Fer-1 treatment groups in chicken embryos. **B:** Quantitative PCR analysis showing the expression levels of viral nucleoprotein in the livers of the three groups. **C-C1:** Western blot analysis demonstrating the expression levels of FRT, xCT, and GPX4 in the livers of the three groups (C), along with the corresponding quantitative analyses (C1). **D-G:** Quantitative PCR data showing the mRNA expression levels of TFR, GPX4, HO1, and SOD1 in the livers of the three groups. **H:** Quantification of lipid peroxidation (LPO) and malondialdehyde (MDA) contents in the liver tissues from each group. **I:** Quantitative PCR analysis of IL-1β, IL-6, and TNF-α mRNA expression levels in the livers of the three groups. **J:** ELISA-based quantification of IL-1β, IL-6, and TNF-α protein levels in the liver tissues from each group. **K:** Quantitative PCR analysis showing the mRNA expression levels of ACTA2 in the livers of the three groups. n = 3 (B–K). * P < 0.05, ** P < 0.01, *** P < 0.001, ns: not significant.

*
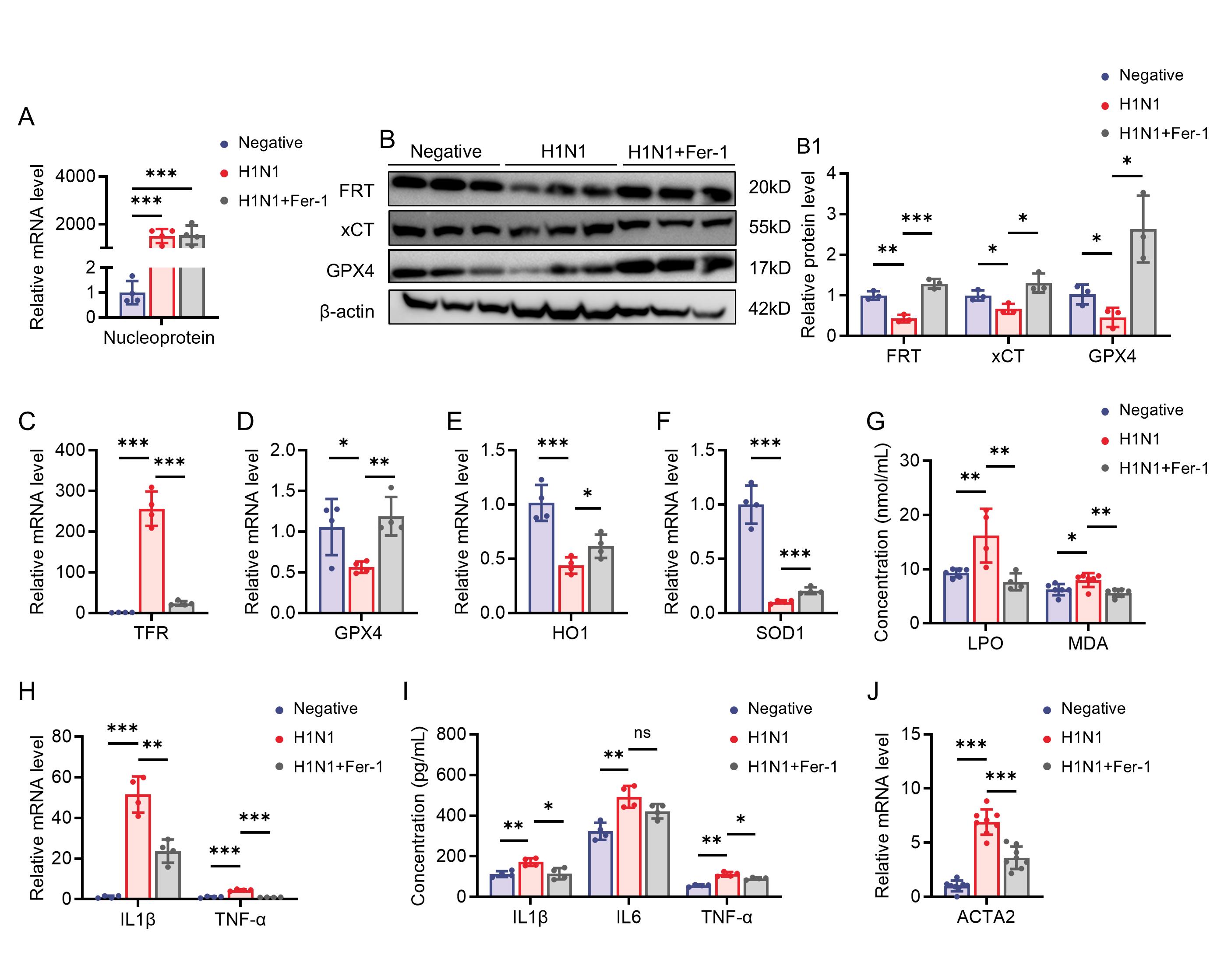
*

***Supplementary Figure 9. Evaluation of the effects of Fer-1 treatment on lung injury in chicken embryos following H1N1 virus infection.***

**A:** Quantitative PCR analysis showing the expression levels of viral nucleoprotein in the lungs of the control, H1N1-infected, and Fer-1 treatment groups. **B-B1:** Western blot analysis demonstrating the expression levels of FRT, xCT, GPX4 in the lungs of the three groups (B), along with corresponding quantitative analyses (B1). **C-F:** Quantitative PCR data showing the mRNA expression levels of TFR, GPX4, HO1, and SOD1 in the lungs of the three groups. **G:** Quantification of lipid peroxidation (LPO) and malondialdehyde (MDA) contents in the lung tissues from each group. **H:** Quantitative PCR analysis of IL-1β and TNF-α mRNA expression levels in the lungs of the three groups. **I:** ELISA-based quantification of IL-1β, IL-6, and TNF-α protein levels in the lung tissues from each group. **J:** Quantitative PCR analysis showing the mRNA expression levels of ACTA2 in the lungs of the three groups. n = 3 (A–J). * P < 0.05, ** P < 0.01, *** P < 0.001*,* ns: not significant.

***Supplementary Table***

***Supplementary Table 1. Search Term***

| Meta-analysis | Search Term | Time Range | Database |
| --- | --- | --- | --- |
| The correlation between H1N1 infection and Preterm birth/ Miscarriage/ maternal death/ severe disease | English search term: (("Pregnant Women"[Mesh]) OR (Woman, Pregnant) OR (Pregnant Woman) OR (Women, Pregnant) OR ("Pregnancy"[Mesh]) OR (Gestation) OR (Pregnancies)) AND (("Influenza A Virus, H1N1 Subtype"[Mesh]) OR (H1N1 subtype) OR (H1N1 subtypes) OR (subtype, H1N1) OR (H1N1 Virus) OR (H1N1 Viruses) OR (Virus, H1N1) OR (H1N1 Influenza Virus) OR (H1N1 Influenza Viruses) OR (Influenza Virus, H1N1) OR (Virus, H1N1 Influenza) OR (Swine-Origin Influenza A H1N1 Virus) OR (Swine Origin Influenza A H1N1 Virus) OR (Influenza A (H1N1)pdm09 Virus) OR (Influenza A (H1N1)pdm09) OR (Influenza A H1N1, Variant Virus) OR (H1N1v Viruses) OR (H1N1v Virus) OR (Virus, H1N1v) OR (H1N1)) AND ((clinical features) OR (clinical characteristics) OR (clinical outcomes)). | 2009.1-2024.10 | PubMed |
| The correlation between H1N1 infection and Liver damage. | English search term: ("Risk Factors"[Mesh]) AND ((clinical features) OR (clinical characteristics) OR (clinical outcomes)) AND ("Influenza A Virus, H1N1 Subtype"[Mesh]). Chinese search term: 'H1N1' * '临床特点'. | 2007.1-2024.10 | PubMed, CNKI |

***Supplementary Table 2. The correlation between H1N1 infection and Preterm birth/ Miscarriage/ maternal death/ severe disease***

| First author，year | Date | Country | Patients (N) | Research Type | Quality |
| --- | --- | --- | --- | --- | --- |
| Naresh,2013 | 2009.7-2010.5.3 | United States | 742 | Retrospective study | 7 |
| Ribeiro,2018 | 2009.6.9-2009.12.1 | Brazil | 233 | Retrospective study | 5 |
| Pramanick,2011 | 2009.8.5-2010.1.31 | India | 164 | Retrospective study | 5 |
| Du,2012 | 2009.6.2-2011.1.22 | China | 116 | Retrospective study | 6 |
| Kusznierz,2012 | 2009.5.1-2009.7.31 | Argentina | 242 | Retrospective study | 5 |
| Dolan,2012 | 2009.5.11-2010.1.31 | U.K. | 395 | Retrospective study | 8 |
| Laake,2018 | 2010.2-2010.9 | Norway | 1258 | Retrospective study | 7 |
| An,2013 | 2009.9-2010.1 | Korea | 755 | Retrospective study | 7 |
| Ramakrishna,2012 | 2009.9-2009.11 | India | 106 | Retrospective study | 6 |
| Newsome,2019 | 2009.4-2009.12 | United States | 1941 | Retrospective study | 8 |
| Zolotusca,2013 | 2009.10-2011.10 | Romania | 148 | Retrospective study | 5 |
| Varner,2011 | 2009.10.1-2010.5.31 | United States | 207 | Retrospective study | 6 |
| Ge´ rardin,2010 | 2009.7.5-2009.10.3 | France | 584 | Retrospective study | 6 |
| R,2011 | 2009.4.23-2010.3.18 | United States | 71 | Retrospective study | 7 |
| Singhal,2014 | 2013.1-2013.3 | India | 24 | Retrospective study | 5 |
| Wang,2010 | 2009.10.3-2009.12.15 | China | 24 | Retrospective study | 6 |
| Choi,2019 | 2009.5-2010.2 | Korea | 290 | Retrospective study | 9 |

References

An, J. H., H.-N. Kim, O.-J. Choi, G.-S. Kim, U. J. Kim, M. O. Jang, S. J. Kang, K.-H. Park, S.-I. Jung, Y. S. Kwon, and H.-C. Jang. 2013. Was 2009 Pandemic Influenza A (H1N1) Mild Among Pregnant Korean Women? *Chonnam Medical Journal* 49 (2).

Choi, W. S., M. J. Choi, J. Y. Noh, J. Y. Song, W. J. Kim, D. W. Park, J. Lee, Y. B. Seo, J. H. Baek, S. Choi, and H. J. Cheong. 2019. Clinical and economic analysis of the 2009 H1N1 influenza pandemic among pregnant Korean women. *The Korean Journal of Internal Medicine* 34 (5):1136-1144.

Cowling, B. J., G. P. Dolan, P. R. Myles, S. J. Brett, J. E. Enstone, R. C. Read, P. J. M. Openshaw, M. G. Semple, W. S. Lim, B. L. Taylor, J. McMenamin, K. G. Nicholson, B. Bannister, and J. S. Nguyen-Van-Tam. 2012. The Comparative Clinical Course of Pregnant and Non-Pregnant Women Hospitalised with Influenza A(H1N1)pdm09 Infection. *PLoS One* 7 (8).

Du, M.-M., N. Jia, J.-J. Suo, Y.-B. Xing, P.-H. Zhang, G. Liu, H.-J. Xiao, J.-S. Zhang, N. Jia, Y. Gao, L.-J. Xie, C.-Y. Deng, S.-W. Ren, and Y.-X. Liu. 2012. Perinatal outcomes and congenital abnormalities in the newborns of women affected by the 2009 pandemic influenza A (H1N1) in Beijing, China. *International Journal of Gynecology & Obstetrics* 116 (2):148-152.

Ellington, S. R., L. K. Hartman, M. Acosta, M. Martinez-Romo, L. Rubinson, D. J. Jamieson, and J. Louie. 2011. Pandemic 2009 influenza A (H1N1) in 71 critically ill pregnant women in California. *American Journal of Obstetrics and Gynecology* 204 (6):S21-S30.

Kusznierz, G., A. Uboldi, G. Sosa, S. Torales, J. Colombo, C. Moyano, H. Escobar, S. Lejona, E. Anchart, A. Gómez, and S. Imaz. 2013. Clinical features of the hospitalized patients with 2009 pandemic influenza A (H1N1) in Santa Fe, Argentina. *Influenza and Other Respiratory Viruses* 7 (3):410-417.

Laake, I., G. Tunheim, A. H. Robertson, O. Hungnes, K. Waalen, S. E. Håberg, S. Mjaaland, and L. Trogstad. 2018. Risk of pregnancy complications and adverse birth outcomes after maternal A(H1N1)pdm09 influenza: a Norwegian population-based cohort study. *BMC Infectious Diseases* 18 (1).

Lin, B., A. F. Ribeiro, A. C. G. Pellini, B. Y. Kitagawa, D. Marques, G. Madalosso, J. Fred, R. K. M. Albernaz, T. R. M. P. Carvalhanas, and D. M. T. Zanetta. 2018. Severe influenza A(H1N1)pdm09 in pregnant women and neonatal outcomes, State of Sao Paulo, Brazil, 2009. *PLoS One* 13 (3).

Naresh, A., B. M. Fisher, K. K. Hoppe, J. Catov, J. Xu, J. Hart, A. M. Lynch, R. Gibbs, D. Eschenbach, M. Gravett, and R. H. Beigi. 2013. A multicenter cohort study of pregnancy outcomes among women with laboratory-confirmed H1N1 influenza. *Journal of Perinatology* 33 (12):939-943.

Newsome, K., C. J. Alverson, J. Williams, A. F. McIntyre, A. D. Fine, C. Wasserman, K. H. Lofy, M. Acosta, J. K. Louie, K. Jones‐Vessey, V. Stanfield, A. Yeung, and S. A. Rasmussen. 2019. Outcomes of infants born to women with influenza A(H1N1)pdm09. *Birth Defects Research* 111 (2):88-95.

Nguyen-Van-Tam, J. S., P. J. M. Openshaw, A. Hashim, E. M. Gadd, W. S. Lim, M. G. Semple, R. C. Read, B. L. Taylor, S. J. Brett, J. McMenamin, J. E. Enstone, C. Armstrong, and K. G. Nicholson. 2010. Risk factors for hospitalisation and poor outcome with pandemic A/H1N1 influenza: United Kingdom first wave (May-September 2009). *Thorax* 65 (7):645-651.

Pramanick, A., S. Rathore, J. V. Peter, M. Moorthy, and J. Lionel. 2011. Pandemic (H1N1) 2009 virus infection during pregnancy in South India. *International Journal of Gynecology & Obstetrics* 113 (1):32-35.

Ramakrishna, K., S. Sampath, J. Chacko, B. Chacko, D. Narahari, H. Veerendra, M. Moorthy, B. Krishna, V. S. Chekuri, R. Raju, D. Shanmugasundaram, K. Pichamuthu, A. Abraham, O. C. Abraham, K. Thomas, P. Mathews, G. Varghese, P. Rupali, and J. Peter. 2012. Clinical profile and predictors of mortality of severe pandemic (H1N1) 2009 virus infection needing intensive care: A multi-centre prospective study from South India. *Journal of Global Infectious Diseases* 4 (3).

Ratner, A. J., P. Gérardin, R. El Amrani, B. Cyrille, M. Gabrièle, P. Guillermin, M. Boukerrou, B. Boumahni, H. Randrianaivo, A. Winer, J.-F. Rouanet, M. Bohrer, M.-C. Jaffar-Bandjee, P.-Y. Robillard, G. Barau, and A. Michault. 2010. Low Clinical Burden of 2009 Pandemic Influenza A (H1N1) Infection during Pregnancy on the Island of La Réunion. *PLoS One* 5 (5).

Singhal, S., N. Sarda, R. Arora, N. Punia, and A. Jain. 2014. Clinical profile & outcome of H1N1 infected pregnant women in a tertiary care teaching hospital of northern India. *Indian J Med Res* 139 (3):454-458.

Varner, M. W., M. M. Rice, B. Anderson, J. E. Tolosa, J. Sheffield, C. Y. Spong, G. Saade, A. M. Peaceman, J. M. Louis, R. J. Wapner, A. T. N. Tita, Y. Sorokin, S. C. Blackwell, M. Prasad, J. M. Thorp, A. Naresh, and J. P. Van Dorsten. 2011. Influenza-Like Illness in Hospitalized Pregnant and Postpartum Women During the 2009–2010 H1N1 Pandemic. *Obstetrics & Gynecology* 118 (3):593-600.

Zolotusca, L., P. Jorgensen, O. Popovici, A. Pistol, F. Popovici, M. A. Widdowson, V. Alexandrescu, A. Ivanciuc, P. Y. Cheng, D. Gross, C. S. Brown, and J. A. Mott. 2013. Risk factors associated with fatal influenza, Romania, October 2009 – May 2011. *Influenza and Other Respiratory Viruses* 8 (1):8-12.

Wang XJ, Jiang RM, Xu YL, Zhang W, Huangfu JK, Wang YB, Wang JJ, Lu LH, Li BS, Jiao YQ, Chen ZH, Guo LM, Li XW. [Clinical characteristics of surviving and fatal cases of severe H1N1 influenza]. Zhonghua Jie He He Hu Xi Za Zhi. 2010 Jun;33(6):406-410. Chinese.

***Supplementary Table 3. The correlation between H1N1 infection and Liver damage.***

| First author，year | Date | Country | Patients (Female) | Average Age | Research Type | Quality |
| --- | --- | --- | --- | --- | --- | --- |
| Nateghian,2020 | 2015.9-2018.3 | Iran | 11080 (6092) | 40.9 | Retrospective study | 7 |
| Peralta,2010 | 2009.4-2009.12 | Spain | 3025 (1372) | Non-severe: 37; severe: 41; | Retrospective study | 6 |
| Li,2019 | 2009.7-2014.5 | China | 66(21) | 52.4 | Retrospective study | 7 |
| Shi,2015 | 2009-2014 | China | 170(66) | 55.4 | Retrospective study | 6 |
| Örnek,2011 | 2009.10-2009.12 | Turkey | 56 | Non-severe: 34; severe: 30; | Retrospective study | 5 |
| Poeppl,2011 | 2009.9-2010.2 | Austrian | 540(203) | 19.3 | Retrospective study | 5 |
| An,2013 | 2009.9-2009.12 | China | 480(288) | 35 | Retrospective study | 7 |
| Guo,2019 | 2018.10-2019.3 | China | 113(58) | Non-severe: 37.1; severe: 45.8; | Retrospective study | 6 |
| Liu,2011 | 2009.12-2010.2 | China | 59(41) | /^a^ | Retrospective study | 5 |
| Duan,2013 | 2009.10-2010.3 | China | 27(10) | Non-severe: 39.2; severe: 37.1; | Retrospective study | 5 |
| Jamoussi,2022 | 2009.10-2009.11 | United States | 120(56) | 48 | Retrospective study | 6 |
| Pečavar,2011 | 2009.10-2010.2 | Austria | 196(96) | Non-severe: 65; severe: 43; | Retrospective study | 5 |
| BISSO,2021 | 2019.1-2009.12 | Argentina | 143(64) | 65 | Retrospective study | 7 |
| Khandaker,2012 | 2009.6-2009.9 | Australia | 190(64) | Non-severe: 46.8; severe: 45.6; | Retrospective study | 6 |
| Damak,2011 | 2009.11-2010.1 | Africa | 32(14) | 36 | Retrospective study | 7 |
| L,2015 | 2009.5-2009.12 | Mexico | 1014（515） | Non-severe: 24; Severe: 35; | Retrospective study | 5 |
| Dou,2021 | 2019.1 - 2019.3 | China | 120（59） | /^a^ | Retrospective study | 6 |
| Shah,2015 | 2013.9-2014.4 | United States | 507（251） | /^a^ | Retrospective study | 7 |

Note: ^a^ Relevant data were not provided in the original article.

References

Carboni Bisso, I., E. Prado, J. Cantos, A. Massó, I. Staneloni, E. San Román, I. Huespe, and M. Las Heras. 2021. Influenza season 2019. Analysis of 143 hospitalized patients. *Medicina (B Aires)* 81 (3):389-395.

Chen, R. J., A. Jamoussi, S. Ayed, T. Merhabene, H. Doghri, J. Ben Khelil, and M. Besbes. 2022. Severe influenza A in a Tunisian ICU sentinel SARI centre: Epidemiological and clinical features. *PLoS One* 17 (7).

Damak, H., K. Chtara, M. Bahloul, H. Kallel, A. Chaari, H. Ksibi, A. Chaari, H. Chelly, N. Rekik, C. Ben Hamida, and M. Bouaziz. 2011. Clinical features, complications and mortality in critically ill patients with 2009 influenza A(H1N1) in Sfax,Tunisia. *Influenza and Other Respiratory Viruses* 5 (4):230-240.

Hlavinkova, L., Z. Kristufkova, and J. Mikas. 2015. Risk factors for severe outcome of cases with pandemic influenza A(H1N1)pdm09. *Bratislava Medical Journal* 116 (06):389-393.

Jamoussi, A., S. Ayed, T. Merhabene, H. Doghri, J. Ben Khelil, and M. Besbes. 2022. Severe influenza A in a Tunisian ICU sentinel SARI centre: Epidemiological and clinical features. *PLoS One* 17 (7):e0270814.

Khandaker, G., H. Rashid, Y. Zurynski, P. C. Richmond, J. Buttery, H. Marshall, M. Gold, T. Walls, B. Whitehead, E. J. Elliott, and R. Booy. 2012. Nosocomial vs community-acquired pandemic influenza A (H1N1) 2009: a nested case–control study. *Journal of Hospital Infection* 82 (2):94-100.

Li, S.-H., M.-J. Hsieh, S.-W. Lin, L.-P. Chuang, C.-S. Lee, L.-C. Chiu, C.-H. Chang, H.-C. Hu, C.-C. Huang, and K.-C. Kao. 2020. Outcomes of severe H1N1 pneumoniae: A retrospective study at intensive care units. *Journal of the Formosan Medical Association* 119 (1):26-33.

Nateghian, A., M. M. Gouya, M. Nabavi, H. Soltani, S. V. Mousavi, E. Agah, H. Erfani, P. Parchami, M. Dadras, and J. L. Robinson. 2020. Demographic, clinical, and virological characteristics of patients with a laboratory-confirmed diagnosis of influenza during three consecutive seasons, 2015/2016–2017/18, in the Islamic Republic of Iran. *Journal of Clinical Virology* 124:104281.

Örnek, T., F. D. Yalçın, S. Ekin, Ş. Yalçın, and M. Yemişen. 2011. Pneumonia in patients with novel influenza A (H1N1) virus in Southeastern Turkey. *Wiener klinische Wochenschrift* 123 (3-4):106-111.

Pečavar, B., K. Nadrah, L. Papst, V. Čeč, T. Kotar, M. Matičič, J. Meglič-Volkar, L. Vidmar, and B. Beović. 2011. Clinical characteristics of adult patients with influenza-like illness hospitalized in general ward during Influenza A H1N1 pandemic 2009/2010. *Wiener klinische Wochenschrift* 123 (21-22):662-667.

Poeppl, W., M. Hell, H. Herkner, B. Stoiser, G. Fritsche, N. Schurz-Bamieh, G. Poeppl, R. Gattringer, N. Jones, M. Maass, A. Egle, and H. Burgmann. 2011. Clinical aspects of 2009 pandemic influenza A (H1N1) virus infection in Austria. *Infection* 39 (4):341-352.

Shah, N. S., J. A. Greenberg, M. C. McNulty, K. S. Gregg, J. Riddell, J. E. Mangino, D. M. Weber, C. L. Hebert, N. S. Marzec, M. A. Barron, F. Chaparro-Rojas, A. Restrepo, V. Hemmige, K. Prasidthrathsint, S. Cobb, L. Herwaldt, V. Raabe, C. R. Cannavino, A. G. Hines, S. H. Bares, P. B. Antiporta, T. Scardina, U. Patel, G. Reid, P. Mohazabnia, S. Kachhdiya, B.-M. Le, C. J. Park, B. Ostrowsky, A. Robicsek, B. A. Smith, J. Schied, M. M. Bhatti, S. Mayer, M. Sikka, I. Murphy-Aguilu, P. Patwari, S. R. Abeles, F. J. Torriani, Z. Abbas, S. Toya, K. Doktor, A. Chakrabarti, S. Doblecki-Lewis, D. J. Looney, and M. Z. David. 2015. Severe Influenza in 33 US Hospitals, 2013–2014: Complications and Risk Factors for Death in 507 Patients. *Infection Control & Hospital Epidemiology* 36 (11):1251-1260.

Shi, S. J., H. Li, M. Liu, Y. M. Liu, F. Zhou, B. Liu, J. X. Qu, and B. Cao. 2015. Mortality prediction to hospitalized patients with influenza pneumonia: PO2/FiO2 combined lymphocyte count is the answer. *The Clinical Respiratory Journal* 11 (3):352-360.

An CJ, Yan XX. [Imaging and clinical characteristics of 480 cases of H1N1 influenza]. Zhonghua Shi Yan He Lin Chuang Gan Ran Bing Za Zhi (Electron Ed). 2013;7(02):197-203. Chinese.

Duan L. [Analysis of clinical data of 27 cases of H1N1 influenza]. Master's Thesis. 2013. Chinese.

Guo JJ, Shi JR, Guo J, Wang YJ. [Comparison of laboratory results between severe and mild H1N1 influenza patients]. Biao Ji Mian Yi Fen Xi Yu Lin Chuang. 2019;26(11):1805-1809. Chinese.

Liu X. [Analysis of H1N1 influenza viral pneumonia cases and the significance of LDH in the mechanism of multi-organ damage caused by the influenza virus]. Master's Thesis. 2011. Chinese.

***Supplementary Table 4. Publication bias assessment for each meta-analysis outcome.***

| Outcome | Number of studies | Egger’s test P-value |
| --- | --- | --- |
| Preterm birth | 6 | 0.415 |
| Miscarriage | 3 | /^a^ |
| maternal death | 8 | 0.112 |
| severe disease | 4 | /^a^ |
| ALT(alanine aminotransferase) | 8 | 0.819 |
| AST(aspartate aminotransferase) | 10 | 0.202 |
| Combined liver disease | 9 | 0.161 |

Note: ^a^ Egger’s test was not performed due to an insufficient number of included studies (less than 5).

***Supplementary Table 5. Antibodies for western blotting.***

| Antibody | Concentration | Article Number | Company |
| --- | --- | --- | --- |
| CD8a | 1：1500 | 8220-01 | SouthernBiotech |
| NF-κB | 1：1000 | TP56372 | abmart |
| Caspase1 | 1：1000 | M025280 | abmart |
| PCNA | 1：3000 | #13110 | cell signaling |
| Bcl2 | 1：1000 | T40056 | abmart |
| Caspase9 | 1：1000 | T40046 | abmart |
| Bax | 1：1000 | T40051 | abmart |
| NQO1 | 1：1000 | 11451-1-AP | proteintech |
| Nrf2 | 1：1000 | T55136 | abmart |
| SOD1 | 1：1000 | #37385 | Cell signaling |
| FTL | 1：1000 | T56955 | abmart |
| FRT | 1：1000 | T55648 | abmart |
| xCT | 1：1000 | T57046 | abmart |
| GPX4 | 1：1000 | ab216876 | abcam |
| α-SMA | 1：1000 | ab5694 | abcam |
| TFR | 1：1000 | T40111 | abmart |
| Bax | 1：1000 | T40051 | abmart |
| HO1 | 1：1000 | 10701-1-AP | proteintech |
| β-actin | 1：5000 | A5441 | Sigma-Aldrich |
| LMNB1 | 1：5000 | ABIN1309510 | Antibodies Online |

***Supplementary Table 6.*** ***Antibodies for Immunofluorescence (IF) and Immunohistochemistry (IHC).***

| Antibody | Concentration | Article Number | Company |
| --- | --- | --- | --- |
| CD3 | 1:100 | 8200-01 | SouthernBiotech |
| CD8a | 1:100 | 8220-01 | SouthernBiotech |
| NF-κB | 1:100 | #3033 | Cell Signaling |
| Caspase1 | 1:100 | M025280 | abmart |
| PCNA | 1:200 | #13110 | Cell signaling |
| P53 | 1:100 | T40060 | abmart |
| α-SMA | 1:100 | ab5694 | abcam |
| Desmin | 1:200 | D76-S | DSHB |
| Bcl2 | 1:100 | #3498 | Cell signaling |
| Caspase9 | 1:100 | T40046 | abmart |
| HO1 | 1:100 | 10701-1-AP | proteintech |
| xCT | 1:100 | T57046 | abmart |
| GPX4 | 1:100 | ab216876 | abcam |

***Supplementary Table 7.*** ***qPCR Primer sequence.***

| Gene | Sapiens |  | Primer |  |  |
| --- | --- | --- | --- | --- | --- |
| IL-1β | chicken | F | CACTGGGCATCAAGGGCTACAAG | | |
|  |  | R | GTCCAGGCGGTAGAAGATGAAGC | | |
| NF-κB | chicken | F | TCAACGCAGGACCTAAAGACAT | | |
|  |  | R | GCAGATAGCCAAGTTCAGGATG | | |
| TNF-a | chicken | F | GCCCTTCCTGTAACCAGATG | | |
|  |  | R | ACACGACAGCCAAGTCAACG | | |
| AMPK | chicken | F | ACCATCTGTCTCGCCCTCATCC | | |
|  |  | R | AATGCCACTTCGCTCTTCTTACACC | | |
| Nrf2 | chicken | F | GGGACGGTGACACAGGAACAAC | | |
|  |  | R | TCCACAGCGGGAAATCAGAAAGATC | | |
| SOD1 | chicken | F | GGTCATCCACTTCCAGCAGCAG | | |
|  |  | R | AAGCCATGATCTCCATCAGACAAGC | | |
| HO1 | chicken | F | GCTGGGAAGGAGAGTGAGAGGAC | | |
|  |  | R | GCGACTGTGGTGGCGATGAAG | | |
| NQO1 | chicken | F | CGAGTGCTTTGTCTACGAGATGGAG | | |
|  |  | R | AGGTCAGCCGCTTCAATCTTCTTC | | |
| GPX4 | chicken | F | CCGCTGTGGAAGTGGCTGAAG | | |
|  |  | R | ATCCTCCATTGGGCTGTACCTTTTC | | |
| FTL | chicken | F | GCCGTGTGAGACAGAGTTTC | | |
|  |  | R | TCAAAATATTCACCCAGCGCC | | |
| FTH | chicken | F | TACCACCAGGACTGCGAAGCC | | |
|  |  | R | TAGGACATGCTGAGGTACACGTAGG | | |
| ASCL4 | chicken | F | ATCACCAGTGCAGAGCTTCT | | |
|  |  | R | AGCTCTTCCACTGTCTGCAT | | |
| CYBB | chicken | F | CTCAGAGGATCAAGTGCGTG | | |
|  |  | R | AGCTTTCATTTGGGCTATCA | | |
| CGTL | chicken | F | GCTGTCGTGACGGTGCCTAATG | | |
|  |  | R | CTCTTGTGGCTGCCTGCTGTC | | |
| SLC11A2 | chicken | F | TCTGTGGGGTGGCGTTCT | | |
|  |  | R | CTGGCTCTGGCTGGGTTT | | |
| SLC40A1 | chicken | F | AGACTGGGTGGACAAGAACTCG | | |
|  |  | R | TGCCAGTTTGCTTCTGTCTTCC | | |
| VDAC3 | chicken | F | CTTGCTCTTCGACCTGGTGT | | |
|  |  | R | AGCCCAACTTTGTGACCTCC | | |
| STEAP3 | chicken | F | TGCCATCACCTCACTTCCAT | | |
|  |  | R | GGGAGGCAGGTAGAACTTGT | | |
| NCOA4 | chicken | F | GTGAGGTGTGGCTGTTTGAG | | |
|  |  | R | CTTTCCAGTCTCTCCAGGC | | |
| TFR | chicken | F | AGGACCGCCTGTCATCTTCTGG | | |
|  |  | R | ACCTACCCTCCACCTCAAGTTGTC | | |
| SAT1 | chicken | F | TGAAGAAACAGCTGCTCCTCCTGT | | |
|  |  | R | TGGCTAGTTCCTTGATCAGTCGCA | | |
| CD86 | chicken | F | CTCTGTCCAAGTCTTTCAGCAC | | |
|  |  | R | CAACCAAAGGGAGGGTATCTCTAAT | | |
| CSF1R | chicken | F | TTGTAAAAGGCAATGCCCGC | | |
|  |  | R | ATGCCGTAGGACCACACATC | | |
| NOS2 | chicken | F | TTGGGATTTCCCCAGGCAAC | | |
|  |  | R | CTTGCCCAATAGCCACCTTC | | |
| CTSG | chicken | F | CATCATGCTGCTCAAGCTGAC | | |
|  |  | R | TCATCAATCAGGCCCCATCC | | |
| HDC | chicken | F | CTGGCTGGCTAAAATGCTGG | | |
|  |  | R | CACTCACAGTGCTCTGCAATA | | |
| MILR1 | chicken | F | ACATACGCAGAGATTGAGCCA | | |
|  |  | R | TCCGACCAGGACCTCTGAAT | | |
| BAFFR | chicken | F | CCTGGCCCCACCATAAGG | | |
|  |  | R | CATTACAGTCTCTCCTCACCCATACA | | |
| CD40 | chicken | F | TGCACACCCTGTGAGAATGGT | | |
|  |  | R | CGTTGCGTTTCCATGTCTCTT | | |
| BINK | chicken | F | CAAGCCGTGCTCTGGGTACT | | |
|  |  | R | CTGTTTTCCGATCGCAGGTT | | |
| RAG1 | chicken | F | CTGGTAACCCCAGTGAAATCCT | | |
|  |  | R | GTGGTTAGAGAAGTGTTGGCCATA | | |
| ACTA2 | chicken | F | CACCCAACTCTGCTGACTGA | | |
|  |  | R | ACACCATCCCCAGAGTCAAG | | |

***Supplementary Table 8.*** ***The statistical description and statistical results in Figure 2.***

| Group | Negative | H1N1 | p-value |
| --- | --- | --- | --- |
|  | Mean ±SD | Mean ±SD |  |
| Figure 2B1 (n≥14) |  |  |  |
| Body length (cm) | 4.09±0.35 | 4.15±0.22 | 0.609 |
| Figure 2B2 (n≥14) |  |  |  |
| Weight (g) | 3.87±0.33 | 3.93±0.28 | 0.576 |
| Figure 2C1 (n=6 photographs from 3 experiments) | |  |  |
| Heart |  | 2.11±0.62 |  |
| Liver |  | 20.63±2.12 |  |
| Lung |  | 8.67±1.64 |  |
| Kidney |  | 1.58±0.24 |  |
| Brain |  | 0.69±0.28 |  |

Note: The means and standard deviations were used for statistical description, statistic test was done with Students t-test and P < 0.05 was considered to indicate a statistically significant difference.

***Supplementary Table 9.*** ***The statistical description and statistical results in Figure 3.***

| Group | Negative | H1N1 | p-value |
| --- | --- | --- | --- |
|  | Mean ±SD | Mean ±SD |  |
| Figure 3A1 (n≥6 photographs from 3 experiments) | |  |  |
| CD3 | 16.50±4.32 | 51.23±9.21 | ＜0.001 |
| CD8 | 24.30±3.81 | 74.84±7.33 | ＜0.001 |
| Figure 3A2 (n=6 photographs from 3 experiments) | |  |  |
| Caspase1 | 57.04±0.98 | 64.96±2.10 | ＜0.001 |
| Figure 3A3 (n=6 photographs from 3 experiments) | |  |  |
| NF-κB | 6.29±2.53 | 19.85±3.67 | ＜0.001 |
| Figure 3B1 (n=3) |  |  |  |
| CD3 | 1.00±0.01 | 2.05±0.43 | 0.013 |
| Caspase1 | 1.00±0.02 | 1.21±0.05 | 0.002 |
| Figure 3C1 (n=3) |  |  |  |
| NF-κB(nucleus) | 1.00±0.40 | 2.05±0.26 | 0.018 |
| NF-κB(cytoplasm) | 1.00±0.28 | 0.61±0.21 | 0.123 |
| Figure 3D (n=9) |  |  |  |
| IL1 | 1.00±0.19 | 3.87±1.42 | ＜0.001 |
| IL6 | 1.00±0.48 | 5.30±2.16 | ＜0.001 |
| NF-κB | 1.00±0.80 | 3.49±1.63 | 0.002 |
| TNF-a | 1.00±0.09 | 5.66±2.65 | ＜0.001 |
| Figure 3F (n≥4) |  |  |  |
| BAFF-R | 1.00±0.53 | 10.20±6.35 | 0.003 |
| CD40 | 1.00±0.40 | 5.30±1.66 | 0.016 |
| BINK | 1.00±0.19 | 2.23±0.58 | ＜0.001 |
| RAG1 | 1.00±0.24 | 4.21±3.19 | 0.014 |
| Figure 3G (n≥4) |  |  |  |
| HDC | 1.00±0.51 | 3.93±3.02 | 0.019 |
| MILR1 | 1.00±0.65 | 17.41±17.20 | 0.017 |
| CTSG | 1.00±0.51 | 5.40±4.56 | 0.017 |
| Figure 3H (n≥4) |  |  |  |
| CD86 | 1.00±0.48 | 0.47±0.45 | 0.195 |
| CSF1R | 1.00±0.35 | 0.76±0.22 | 0.144 |
| NOS2 | 1.00±0.40 | 0.10±0.06 | ＜0.001 |

Note: The means and standard deviations were used for statistical description, statistic test was done with Students t-test and P < 0.05 was considered to indicate a statistically significant difference.

***Supplementary Table 10.*** ***The statistical description and statistical results in Figure 4.***

| Group | Negative | H1N1 | p-value |
| --- | --- | --- | --- |
|  | Mean ±SD | Mean ±SD |  |
| Figure 4A1 (n=12 photographs from 3 experiments) | |  |  |
| PCNA | 87.17±7.30 | 21.42±5.53 | ＜0.001 |
| Figure 4B1 (n=6 photographs from 3 experiments) | |  |  |
| P53 | 5.00±0.52 | 86.11±20.18 | ＜0.001 |
| Figure 4C1 (n=3) |  |  |  |
| PCNA | 1.00±0.02 | 0.43±0.05 | ＜0.001 |
| Caspase9 | 1.00±0.18 | 5.59±0.18 | ＜0.001 |
| Bax/Bcl2 | 1.00±0.11 | 6.32±3.06 | 0.039 |
| Figure 4D1 (n=16 photographs from 4 experiments) | |  |  |
| α-SMA | 1.17±0.26 | 3.99±0.83 | ＜0.001 |
| Sirius Staining | 5.25±1.50 | 13.34±1.72 | ＜0.001 |

Note: The means and standard deviations were used for statistical description, statistic test was done with Students t-test and P < 0.05 was considered to indicate a statistically significant difference.

***Supplementary Table 11.*** ***The statistical description and statistical results in Figure 5.***

| Group | Negative | H1N1 | p-value |
| --- | --- | --- | --- |
|  | Mean ±SD | Mean ±SD |  |
| Figure 5A1 (n=6 photographs from 3 experiments) | |  |  |
| PCNA（Alveolar epithelial cells） | 0.88±0.05 | 0.61±0.07 | ＜0.001 |
| PCNA（Interstitial lung cells） | 0.89±0.02 | 0.50±0.02 | ＜0.001 |
| Figure 5B1 (n=6 photographs from 3 experiments) | |  |  |
| Caspase9 | 30.57±5.31 | 74.72±5.83 | ＜0.001 |
| Figure 5B2 (n=6 photographs from 3 experiments) | |  |  |
| Bcl2 | 1.61±0.24 | 0.49±0.05 | ＜0.001 |
| Figure 5C1 (n=3) |  |  |  |
| PCNA | 1.00±0.26 | 0.45±0.09 | 0.026 |
| Bax/Bcl2 | 1.00±0.03 | 3.40±1.07 | 0.018 |
| Figure 5D1 (n=16 photographs from 4 experiments) | |  |  |
| α-SMA | 8.29±1.16 | 23.29±5.85 | ＜0.001 |
| Sirius Staining | 6.96±2.41 | 20.00±3.37 | ＜0.001 |

Note: The means and standard deviations were used for statistical description, statistic test was done with Students t-test and P < 0.05 was considered to indicate a statistically significant difference.

***Supplementary Table 12.*** ***The statistical description and statistical results in Figure 7.***

| Group | Negative | H1N1 | p-value |
| --- | --- | --- | --- |
|  | Mean ±SD | Mean ±SD |  |
| Figure 7A1 (n=16 photographs from 4 experiments) | |  |  |
| HO1 | 0.93±0.29 | 0.38±0.18 | ＜0.001 |
| Figure 7B1 (n=3) |  |  |  |
| NQO1 | 1.00±0.03 | 0.49±0.17 | 0.006 |
| Nrf2 | 1.00±0.05 | 1.40±0.13 | 0.008 |
| SOD1 | 1.00±0.07 | 0.11±0.04 | ＜0.001 |
| Figure 7C (n≥6) |  |  |  |
| Nrf2 | 1.00±0.68 | 3.81±0.50 | ＜0.001 |
| SOD1 | 1.00±0.20 | 0.03±0.03 | ＜0.001 |
| HO1 | 1.00±0.18 | 0.11±0.02 | ＜0.001 |
| NQO1 | 1.00±0.15 | 0.11±0.01 | ＜0.001 |
| Figure 7D (n≥9) |  |  |  |
| TFR | 1.00±0.50 | 6.46±0.67 | ＜0.001 |
| GPX4 | 1.00±0.76 | 0.24±0.17 | 0.003 |
| FTL | 1.00±0.65 | 0.05±0.02 | ＜0.001 |
| FTH | 1.00±0.60 | 0.05±0.03 | ＜0.001 |
| ASCL4 | 1.00±0.32 | 2.34±0.97 | 0.001 |
| Figure 7E1 (n=12 photographs from 3 experiments) | |  |  |
| xCT | 17.95±2.77 | 13.07±1.94 | ＜0.001 |
| Figure 7E2 (n=6 photographs from 3 experiments) | |  |  |
| GPX4 | 80.05±2.51 | 2.71±0.62 | ＜0.001 |
| Figure 7F1 (n=3) |  |  |  |
| FTL | 1.00±0.08 | 0.09±0.03 | ＜0.001 |
| FRT | 1.00±0.03 | 0.32±0.07 | ＜0.001 |
| xCT | 1.00±0.11 | 0.62±0.07 | 0.007 |
| GPX4 | 1.00±0.10 | 0.67±0.05 | 0.006 |

Note: The means and standard deviations were used for statistical description, statistic test was done with Students t-test and P < 0.05 was considered to indicate a statistically significant difference.

***Supplementary Table 13.*** ***The statistical description and statistical results in Figure 8.***

| Group | Negative | H1N1 | p-value |
| --- | --- | --- | --- |
|  | Mean ±SD | Mean ±SD |  |
| Figure 8A1 (n=16 photographs from 4 experiments) | |  |  |
| HO1 | 2.23±0.66 | 0.58±0.28 | ＜0.001 |
| Figure 8B1 (n=3) |  |  |  |
| NQO1 | 1.00±0.03 | 0.60±0.04 | ＜0.001 |
| HO1 | 1.00±0.14 | 0.70±0.05 | 0.022 |
| SOD1 | 1.00±0.09 | 0.54±0.08 | 0.003 |
| Figure 8C (n≥9) |  |  |  |
| SOD1 | 1.00±0.28 | 0.31±0.10 | ＜0.001 |
| HO1 | 1.00±0.13 | 0.67±0.26 | 0.004 |
| Nrf2 | 1.00±0.13 | 2.43±0.17 | ＜0.001 |
| NQO1 | 1.00±0.10 | 0.22±0.13 | ＜0.001 |
| Figure 8D (n=9) |  |  |  |
| ASCL4 | 1.00±0.66 | 2.82±0.37 | ＜0.001 |
| FTL | 1.00±0.26 | 0.26±0.08 | ＜0.001 |
| TFR | 1.00±0.17 | 3.24±0.88 | ＜0.001 |
| GPX4 | 1.00±0.07 | 0.27±0.06 | ＜0.001 |
| Figure 8E1 (n=16 photographs from 4 experiments) | |  |  |
| xCT | 6.92±1.26 | 2.63±1.09 | ＜0.001 |
| Figure 8E2 (n=16 photographs from 4 experiments) | |  |  |
| GPX4 | 2.51±0.83 | 1.09±0.48 | ＜0.001 |
| Figure 8F1 (n=3) |  |  |  |
| xCT | 1.00±0.09 | 0.75±0.06 | 0.015 |
| FRT | 1.00±0.13 | 0.24±0.02 | ＜0.001 |
| FTL | 1.00±0.02 | 0.57±0.05 | ＜0.001 |
| GPX4 | 1.00±0.10 | 0.41±0.06 | ＜0.001 |

Note: The means and standard deviations were used for statistical description, statistic test was done with Students t-test and P < 0.05 was considered to indicate a statistically significant difference.

***Supplementary Table 14.*** ***The statistical description and statistical results in Supplementary Figure 4.***

| Group | Negative | H1N1 | p-value |
| --- | --- | --- | --- |
|  | Mean ±SD | Mean ±SD |  |
| Supplementary Figure 4B1 (n=18 photographs from 4 experiments) | | |  |
| PCNA | 2.61±1.88 | 15.83±3.79 | ＜0.001 |

Note: The means and standard deviations were used for statistical description, statistic test was done with Students t-test and P < 0.05 was considered to indicate a statistically significant difference.

***Supplementary Table 15.*** ***The statistical description and statistical results in Supplementary Figure 5.***

| Group | Negative | H1N1 | p-value |
| --- | --- | --- | --- |
|  | Mean ±SD | Mean ±SD |  |
| Supplementary Figure 5A1 (n=12 photographs from 3 experiments) | | |  |
| Desmin | 2.28±0.59 | 3.12±0.56 | 0.002 |

Note: The means and standard deviations were used for statistical description, statistic test was done with Students t-test and P < 0.05 was considered to indicate a statistically significant difference.

***Supplementary Table 16. The statistical description and statistical results in Supplementary Figure 5.***

| Group | Mean Difference | Std. Error | p-value |
| --- | --- | --- | --- |
| Supplementary Figure 5B1 (n=9 photographs from 3 experiments) α-SMA | | |  |
| Negative vs H1N1-1 | -1.68 | 0.68 | ＜0.001 |
| H1N1-1 vs H1N1-2 | -0.96 | 0.71 | 0.004 |
| H1N1-2 vs H1N1-3 | -3.50 | 1.33 | ＜0.001 |
| Supplementary Figure 5B2 (n=12 photographs from 3 experiments) Nucleoprotein | | |  |
| H1N1-1 vs H1N1-2 | -7.84 | 2.30 | ＜0.001 |
| H1N1-2 vs H1N1-3 | -9.19 | 2.93 | ＜0.001 |

Note: The means and standard deviations were used for statistical description, statistic test was done with Students t-test and P < 0.05 was considered to indicate a statistically significant difference.

***Supplementary Table 17.*** ***The statistical description and statistical results in Supplementary Figure 6.***

| Group | Negative | H1N1 | p-value |
| --- | --- | --- | --- |
|  | Mean ±SD | Mean ±SD |  |
| Supplementary Figure 6A (n=5) |  |  |  |
| ferrous | 21.42±2.13 | 32.18±2.07 | ＜0.001 |
| Total iron | 33.84±1.84 | 47.21±2.56 | ＜0.001 |
| Supplementary Figure 6A1 (n=5) |  |  |  |
| Ferrous/Total iron | 0.62±0.04 | 0.68±0.02 | 0.017 |
| Supplementary Figure 6B1 (n≥6) |  |  |  |
| CGTL | 1.00±0.35 | 0.44±0.13 | ＜0.001 |
| SLC40A1 | 1.00±0.07 | 2.60±1.04 | 0.004 |
| VDAC3 | 1.00±0.22 | 2.81±0.44 | ＜0.001 |
| STEAP3 | 1.00±0.64 | 3.77±1.01 | ＜0.001 |
| NCOA4 | 1.00±0.81 | 2.22±2.17 | 0.006 |
| TFRC | 1.00±0.30 | 5.99±4.91 | ＜0.001 |
| SAT1 | 1.00±0.23 | 1.94±1.53 | ＜0.001 |

Note: The means and standard deviations were used for statistical description, statistic test was done with Students t-test and P < 0.05 was considered to indicate a statistically significant difference.

***Supplementary Table 18. The statistical description and statistical results in Supplementary Figure 7.***

| Group | Negative | H1N1 | p-value |
| --- | --- | --- | --- |
|  | Mean ±SD | Mean ±SD |  |
| Figure 7A1 (n=3) |  |  |  |
| Nrf2(nucleus) | 1.00±0.55 | 2.49±0.72 | 0.047 |
| Nrf2(cytoplasm) | 1.00±0.51 | 0.30±0.08 | 0.079 |
| Figure 7B1 (n=3) |  |  |  |
| Nrf2(nucleus) | 1.00±0.05 | 1.61±0.36 | 0.033 |
| Nrf2(cytoplasm) | 1.00±0.36 | 0.72±0.51 | 0.485 |
| Figure 7C1 (n=25 photographs from 3 experiments) | |  |  |
| Nrf2 | 1.00±0.13 | 1.30±0.19 | ＜0.001 |
| Figure 7C2 (n≥16 photographs from 3 experiments) | |  |  |
| Nrf2 | 1.00±0.35 | 1.34±0.40 | 0.011 |

Note: The means and standard deviations were used for statistical description, statistic test was done with Students t-test and P < 0.05 was considered to indicate a statistically significant difference.

***Supplementary Table 19. The statistical description and statistical results in Supplementary Figure 8.***

| Group | Mean Difference | Std. Error | p-value |
| --- | --- | --- | --- |
| Supplementary Figure 8B (n=4) Nucleoprotein | |  |  |
| Negative vs H1N1 | 781.70 | 75.17 | 0.002 |
| Negative vs H1N1+Fer-1 | 272.60 | 192.40 | 0.251 |
| Supplementary Figure 8C1 (n=3) FRT | |  |  |
| Negative vs H1N1 | 0.49 | 0.10 | 0.007 |
| H1N1 vs H1N1+Fer-1 | -1.67 | 0.13 | ＜0.001 |
| Supplementary Figure 8C1 (n=3) Xct | |  |  |
| Negative vs H1N1 | 0.43 | 0.07 | 0.004 |
| H1N1 vs H1N1+Fer-1 | -0.87 | 2.93 | ＜0.001 |
| Supplementary Figure 8C1 (n=3) GPX4 | |  |  |
| Negative vs H1N1 | 0.68 | 0.09 | 0.002 |
| H1N1 vs H1N1+Fer-1 | -1.39 | 0.06 | ＜0.001 |
| Supplementary Figure 8D (n=4) TFR | |  |  |
| Negative vs H1N1 | -154.70 | 5.63 | ＜0.001 |
| H1N1 vs H1N1+Fer-1 | 144.80 | 6.78 | ＜0.001 |
| Supplementary Figure 8E (n=4) GPX4 | |  |  |
| Negative vs H1N1 | 0.53 | 0.05 | ＜0.001 |
| H1N1 vs H1N1+Fer-1 | -0.20 | 0.08 | 0.043 |
| Supplementary Figure 8F (n=4) HO1 | |  |  |
| Negative vs H1N1 | 0.32 | 0.11 | 0.031 |
| H1N1 vs H1N1+Fer-1 | -0.30 | 0.11 | 0.034 |
| Supplementary Figure 8G (n=8) SOD1 | |  |  |
| Negative vs H1N1 | 0.38 | 0.10 | 0.002 |
| H1N1 vs H1N1+Fer-1 | -1.38 | 0.10 | ＜0.001 |
| Supplementary Figure 8H (n≥4) LPO | |  |  |
| Negative vs H1N1 | -7.34 | 2.11 | 0.008 |
| H1N1 vs H1N1+Fer-1 | 9.23 | 2.03 | 0.001 |
| Supplementary Figure 8H (n≥5) MDA | |  |  |
| Negative vs H1N1 | -2.15 | 0.45 | ＜0.001 |
| H1N1 vs H1N1+Fer-1 | 2.19 | 0.56 | 0.003 |
| Supplementary Figure 8I (n=4) IL1β | |  |  |
| Negative vs H1N1 | -26.36 | 1.81 | ＜0.001 |
| H1N1 vs H1N1+Fer-1 | 22.50 | 2.18 | ＜0.001 |
| Supplementary Figure 8I (n=4) IL6 | |  |  |
| Negative vs H1N1 | -7.73 | 0.83 | ＜0.001 |
| H1N1 vs H1N1+Fer-1 | 5.66 | 1.35 | 0.006 |
| Supplementary Figure 8I (n=4) TNF-α | |  |  |
| Negative vs H1N1 | -2.97 | 0.38 | ＜0.001 |
| H1N1 vs H1N1+Fer-1 | 3.53 | 0.37 | ＜0.001 |
| Supplementary Figure 8J (n=4) IL1β | |  |  |
| Negative vs H1N1 | -37.03 | 8.48 | 0.005 |
| H1N1 vs H1N1+Fer-1 | -15.42 | 13.53 | 0.298 |
| Supplementary Figure 8J (n=4) IL6 | |  |  |
| Negative vs H1N1 | -158.70 | 38.94 | 0.007 |
| H1N1 vs H1N1+Fer-1 | 46.14 | 42.95 | 0.324 |
| Supplementary Figure 8J (n=4) TNF-α | |  |  |
| Negative vs H1N1 | -33.38 | 9.43 | 0.012 |
| H1N1 vs H1N1+Fer-1 | -11.72 | 6.13 | 0.104 |
| Supplementary Figure 8K (n=4) ACTA2 | |  |  |
| Negative vs H1N1 | -0.95 | 0.08 | ＜0.001 |
| H1N1 vs H1N1+Fer-1 | 0.80 | 0.15 | ＜0.001 |

Note: The means and standard deviations were used for statistical description, statistic test was done with Students t-test and P < 0.05 was considered to indicate a statistically significant difference.

***Supplementary Table 20. The statistical description and statistical results in Supplementary Figure 9.***

| Group | Mean Difference | Std. Error | p-value |
| --- | --- | --- | --- |
| Supplementary Figure 9A (n=4) Nucleoprotein | |  |  |
| Negative vs H1N1 | -1509.00 | 148.80 | ＜0.001 |
| Negative vs H1N1+Fer-1 | -1547.00 | 200.00 | ＜0.001 |
| Supplementary Figure 9B1 (n=3) FRT |  |  |  |
| Negative vs H1N1 | 0.57 | 0.08 | 0.002 |
| H1N1 vs H1N1+Fer-1 | -0.86 | 0.09 | ＜0.001 |
| Supplementary Figure 9B1 (n=3) Xct |  |  |  |
| Negative vs H1N1 | 0.33 | 0.10 | 0.032 |
| H1N1 vs H1N1+Fer-1 | -0.64 | 0.15 | 0.014 |
| Supplementary Figure 9B1 (n=3) GPX4 |  |  |  |
| Negative vs H1N1 | 0.57 | 0.20 | 0.045 |
| H1N1 vs H1N1+Fer-1 | -2.17 | 0.49 | 0.012 |
| Supplementary Figure 9C (n=4) TFR |  |  |  |
| Negative vs H1N1 | -255.40 | 21.05 | ＜0.001 |
| H1N1 vs H1N1+Fer-1 | 232.80 | 21.23 | ＜0.001 |
| Supplementary Figure 9D (n=4) GPX4 |  |  |  |
| Negative vs H1N1 | 0.49 | 0.18 | 0.031 |
| H1N1 vs H1N1+Fer-1 | -0.63 | 0.12 | 0.002 |
| Supplementary Figure 9E (n=4) HO1 |  |  |  |
| Negative vs H1N1 | 0.58 | 0.09 | ＜0.001 |
| H1N1 vs H1N1+Fer-1 | -0.18 | 0.07 | 0.035 |
| Supplementary Figure 9F (n=4) SOD1 |  |  |  |
| Negative vs H1N1 | 0.90 | 0.09 | ＜0.001 |
| H1N1 vs H1N1+Fer-1 | -0.10 | 0.02 | ＜0.001 |
| Supplementary Figure 9G (n≥4) LPO |  |  |  |
| Negative vs H1N1 | -6.87 | 2.00 | 0.009 |
| H1N1 vs H1N1+Fer-1 | 8.49 | 2.60 | 0.017 |
| Supplementary Figure 9G (n≥5) MDA |  |  |  |
| Negative vs H1N1 | -1.76 | 0.68 | 0.026 |
| H1N1 vs H1N1+Fer-1 | 2.37 | 0.60 | 0.003 |
| Supplementary Figure 9H (n=4) IL1β |  |  |  |
| Negative vs H1N1 | -50.45 | 4.47 | ＜0.001 |
| H1N1 vs H1N1+Fer-1 | 27.86 | 5.30 | 0.002 |
| Supplementary Figure 9H (n=4) TNF-α |  |  |  |
| Negative vs H1N1 | -3.42 | 0.29 | ＜0.001 |
| H1N1 vs H1N1+Fer-1 | 3.50 | 0.22 | 0.000 |
| Supplementary Figure 9I (n=4) IL1β |  |  |  |
| Negative vs H1N1 | -59.21 | 11.91 | 0.003 |
| H1N1 vs H1N1+Fer-1 | 57.79 | 16.89 | 0.014 |
| Supplementary Figure 9I (n=4) IL6 |  |  |  |
| Negative vs H1N1 | -169.90 | 34.17 | 0.003 |
| H1N1 vs H1N1+Fer-1 | 71.15 | 32.15 | 0.069 |
| Supplementary Figure 9I (n=4) TNF-α |  |  |  |
| Negative vs H1N1 | -55.60 | 6.20 | ＜0.001 |
| H1N1 vs H1N1+Fer-1 | 20.87 | 6.20 | 0.015 |
| Supplementary Figure 9J (n=4) ACTA2 |  |  |  |
| Negative vs H1N1 | -5.90 | 0.45 | ＜0.001 |
| H1N1 vs H1N1+Fer-1 | 3.29 | 0.55 | ＜0.001 |

Note: The means and standard deviations were used for statistical description, statistic test was done with Students t-test and P < 0.05 was considered to indicate a statistically significant difference.
