# Supplementary material for: Influenza A Virus (H1N1) Infection Induces Ferroptosis to Promote Developmental Injury in Fetal Tissues
Source: Cell Prolif. 2025 Aug 26;59(3):e70117. doi: 10.1111/cpr.70117 (PMC12961522; doi:10.1111/cpr.70117)

**Supplementary metadata of Western blot**

**Figure 3B.**

CD8a 25kD, 34kD, 55kD


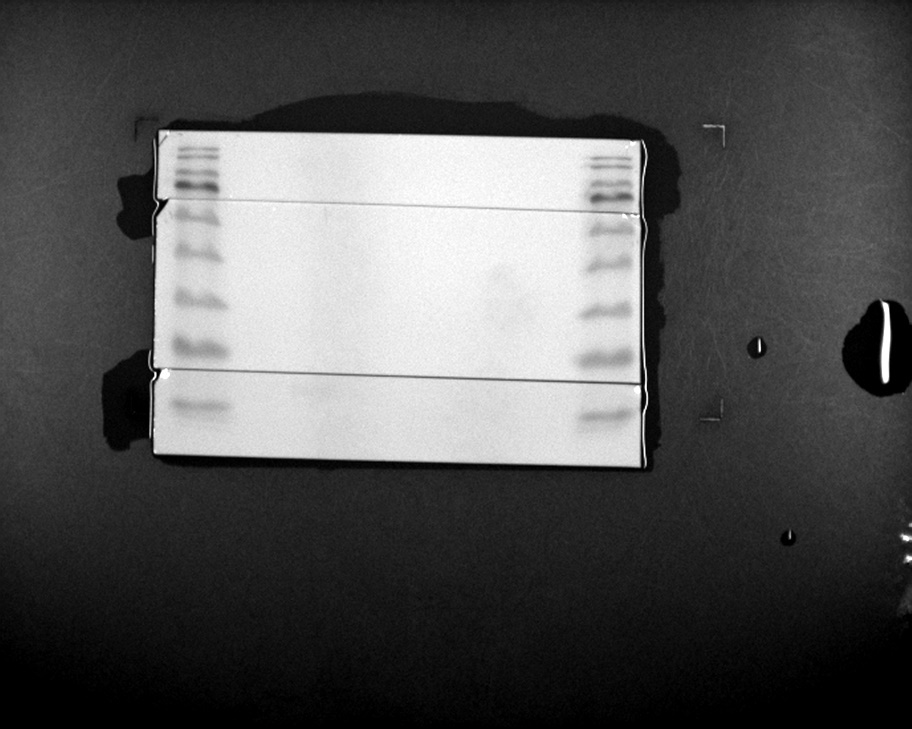

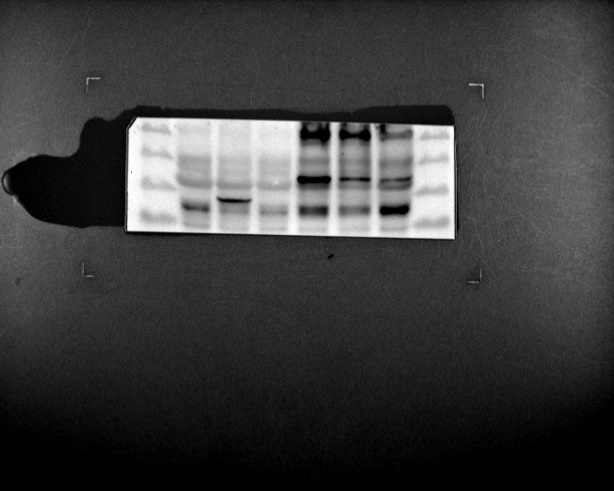


β-actin 42kD


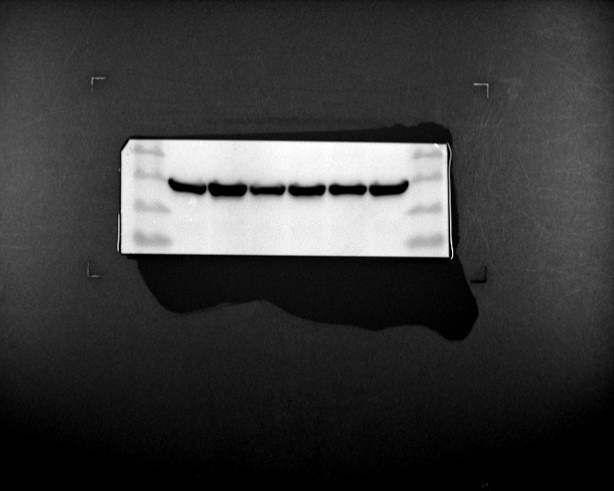


Caspase1 45kD


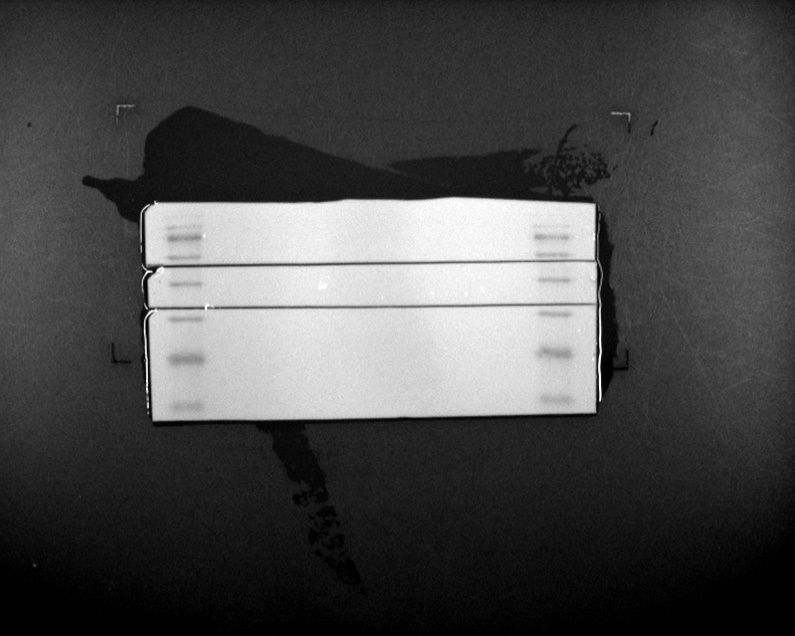




β-actin 42kD


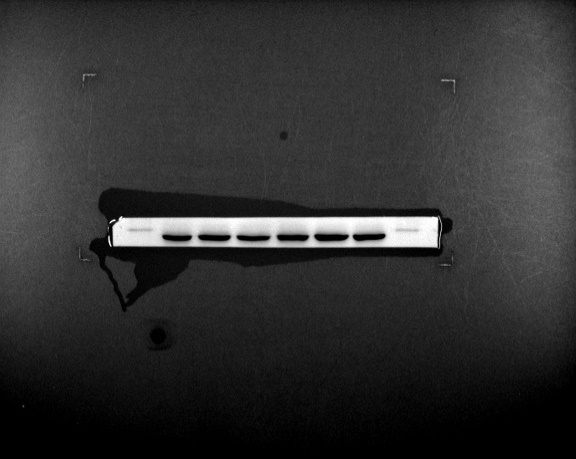


**Figure 3C**

NF-κB (P65) 65kD


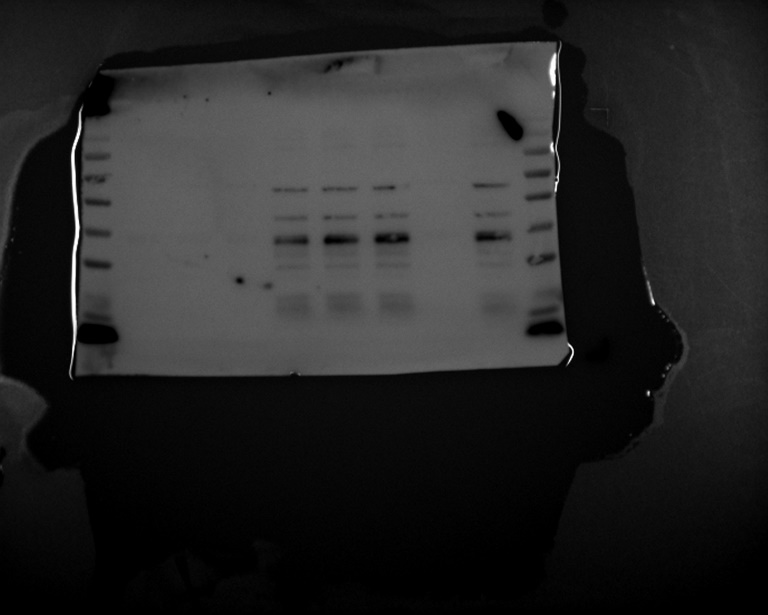


LMNB1 30kD β-actin 42kD


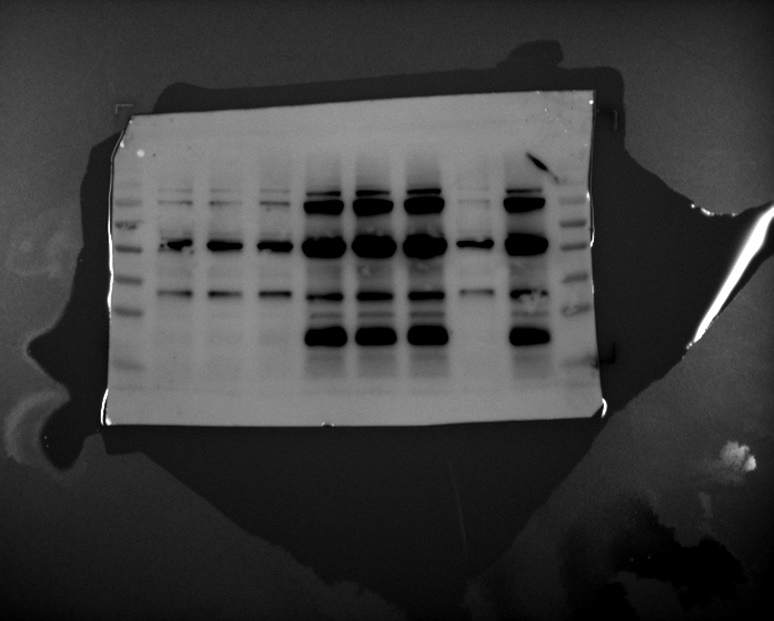

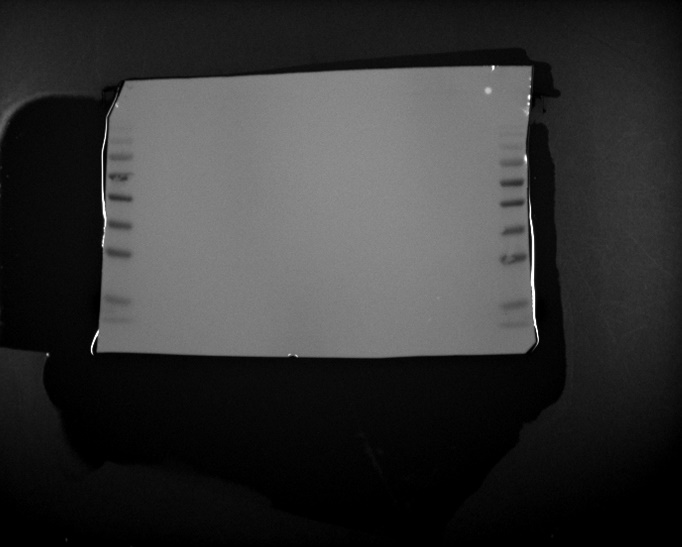


NF-κB (P65) 65kD


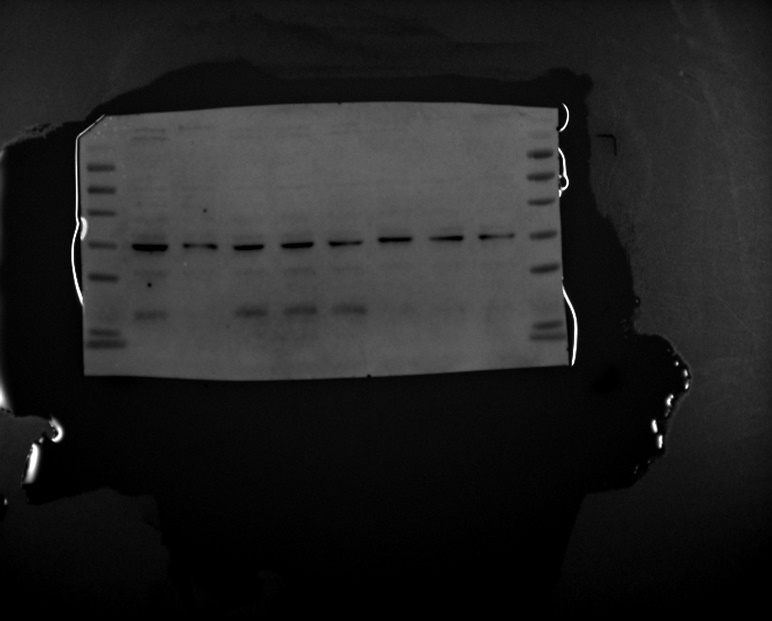


β-actin 42kD LMNB1 30kD


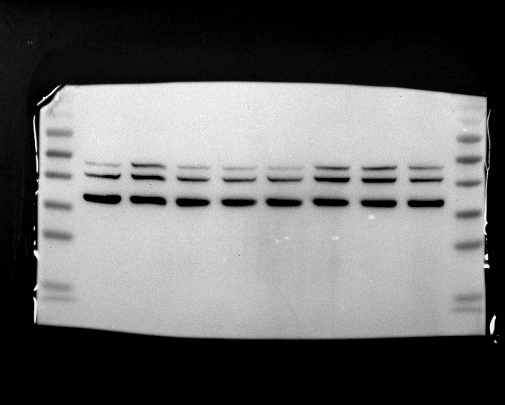

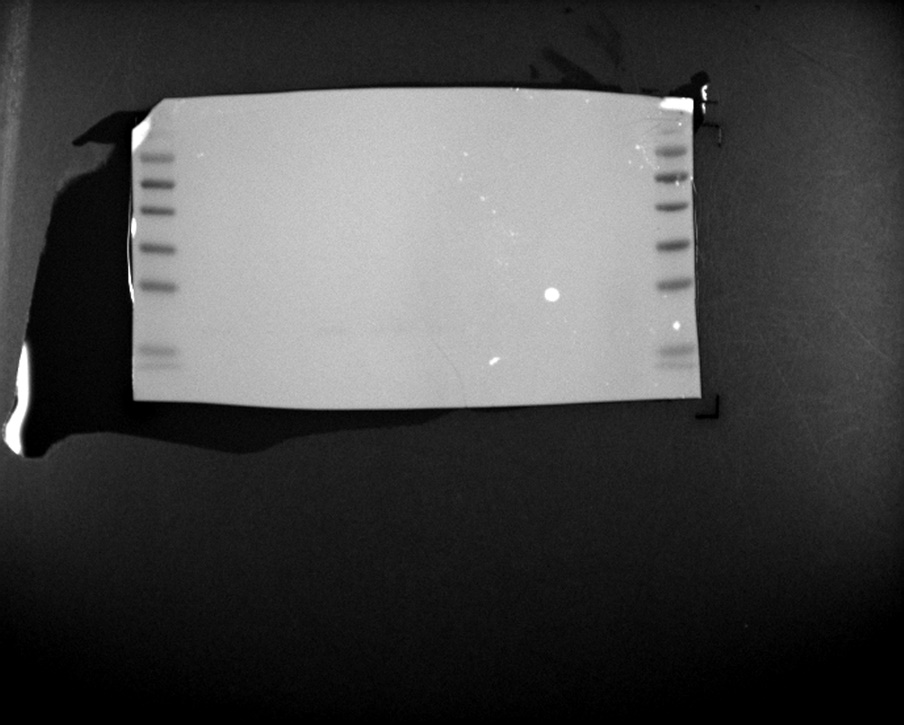


**Figure 4C.**

PCNA 34kD


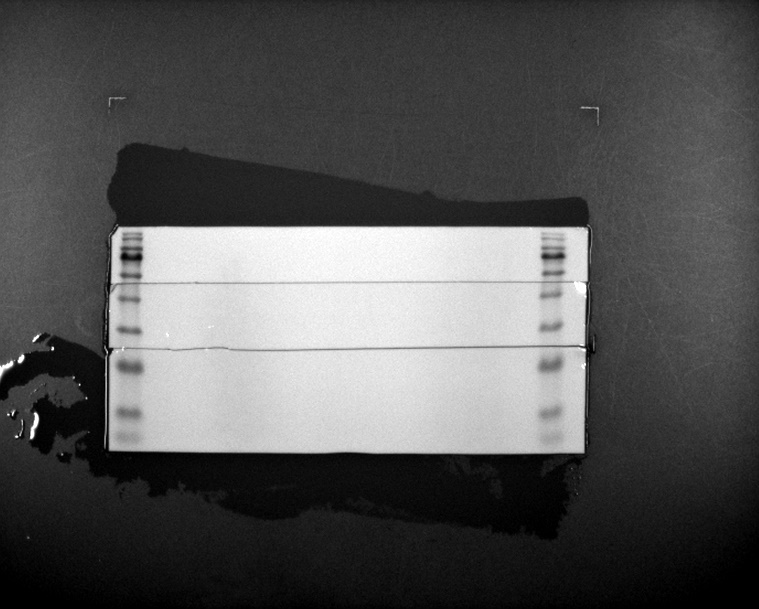




β-actin 42kD


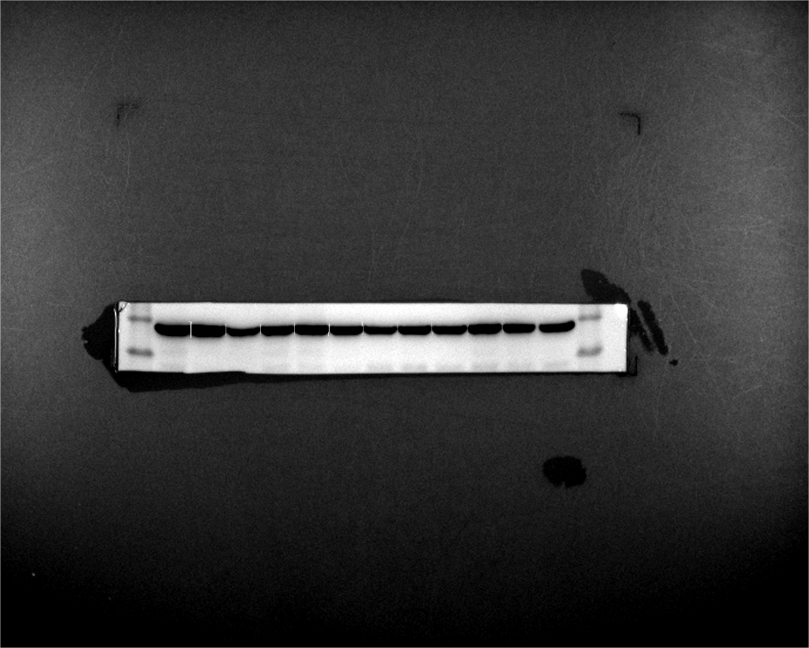


Caspase9 46kD


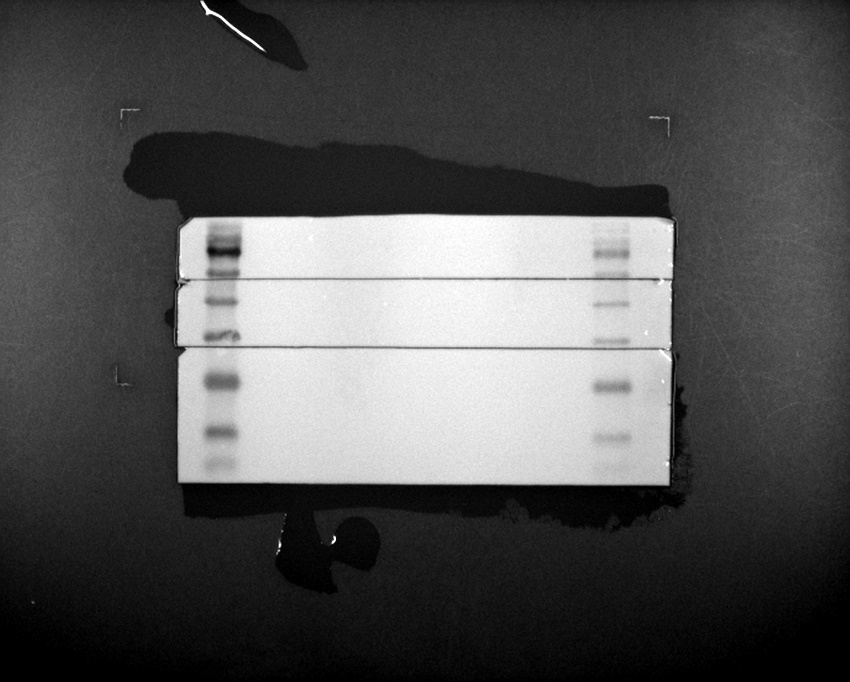

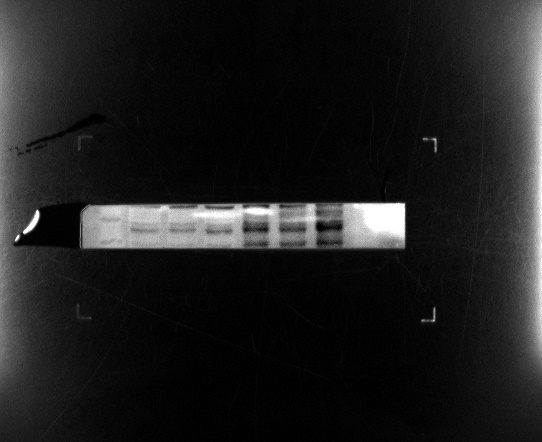


β-actin 42kD


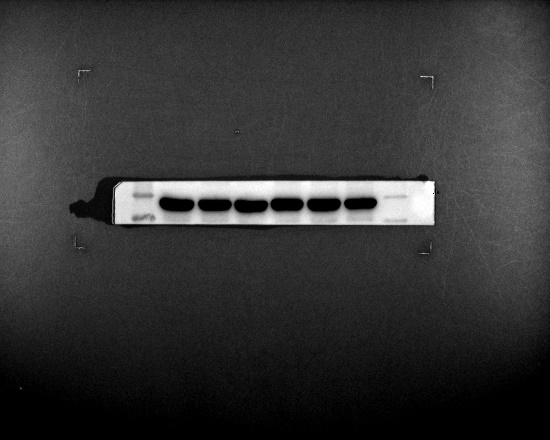


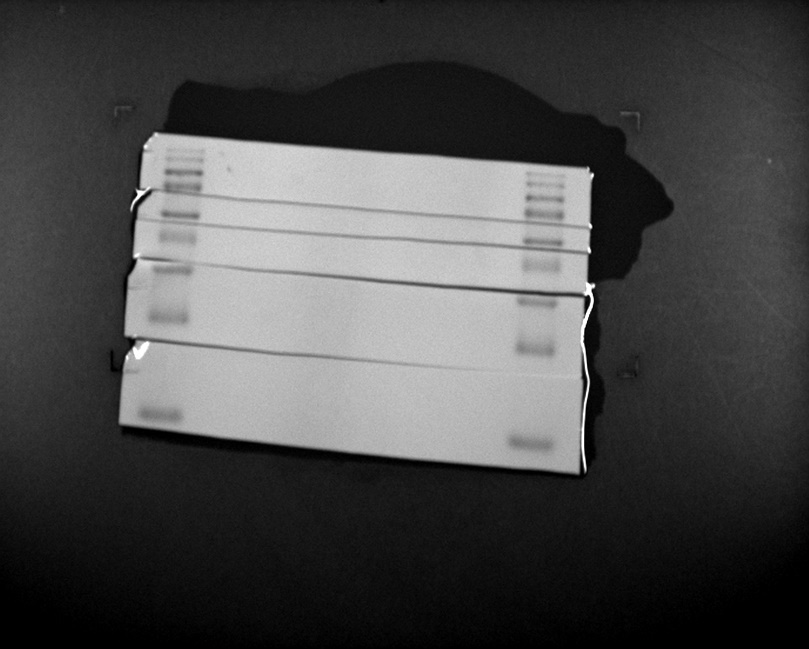
Bax 21kD


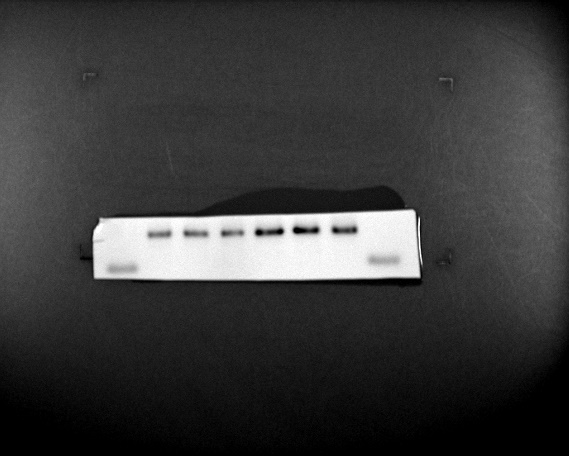


β-actin 42kD


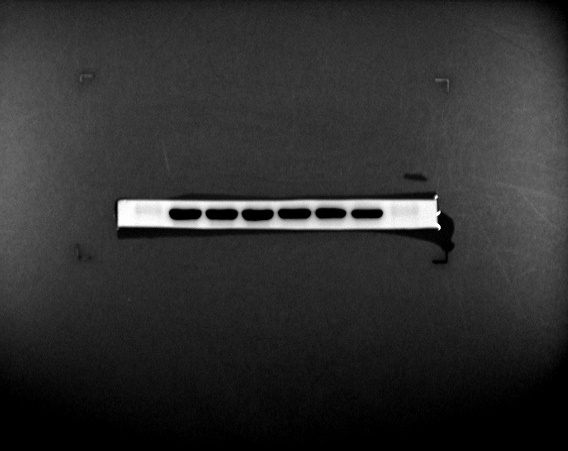


Bcl2 26kD


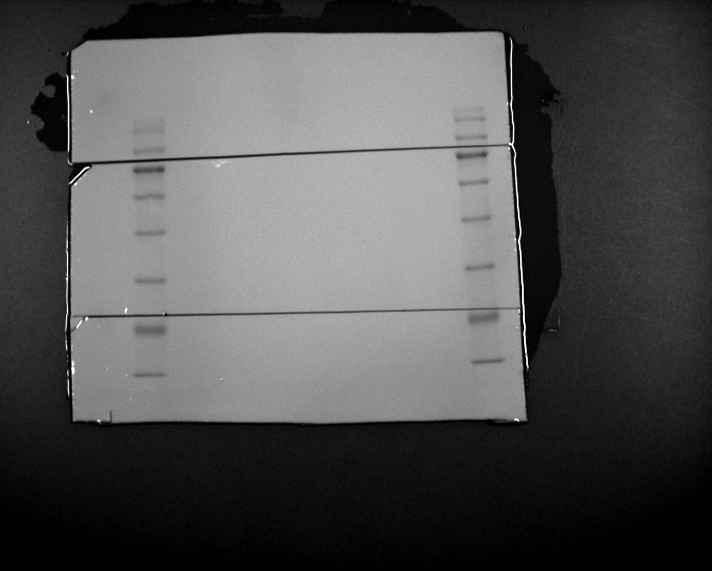

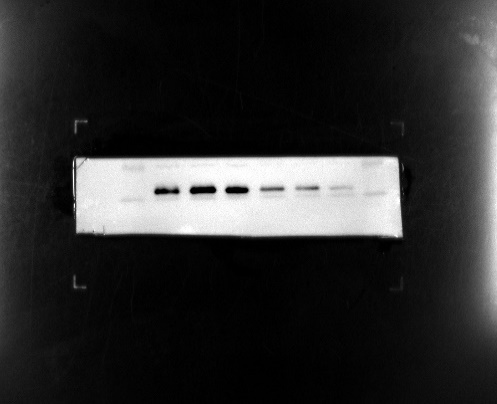


β-actin 42kD


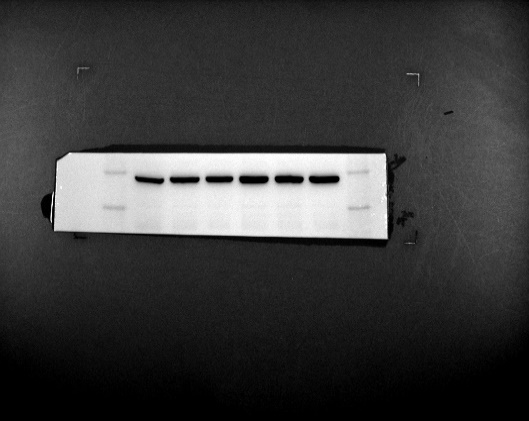


**Figure 5C.**

PCNA 34kD


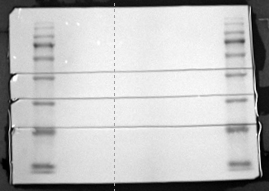

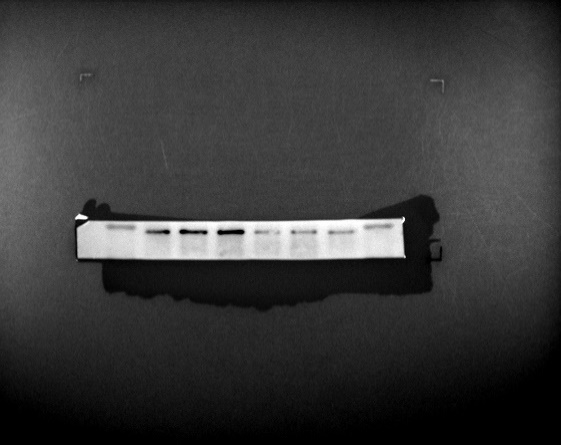


β-actin 42kD


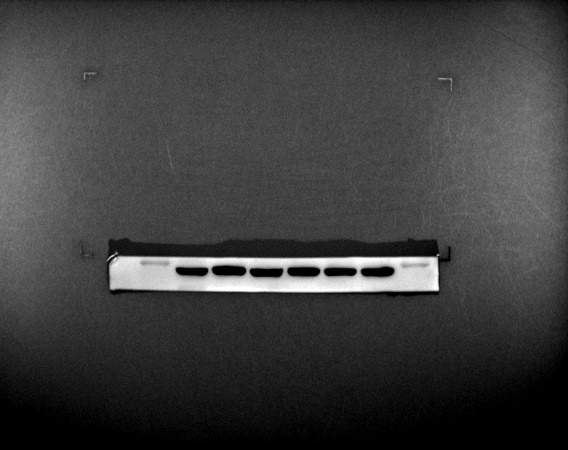


Bax 21kD


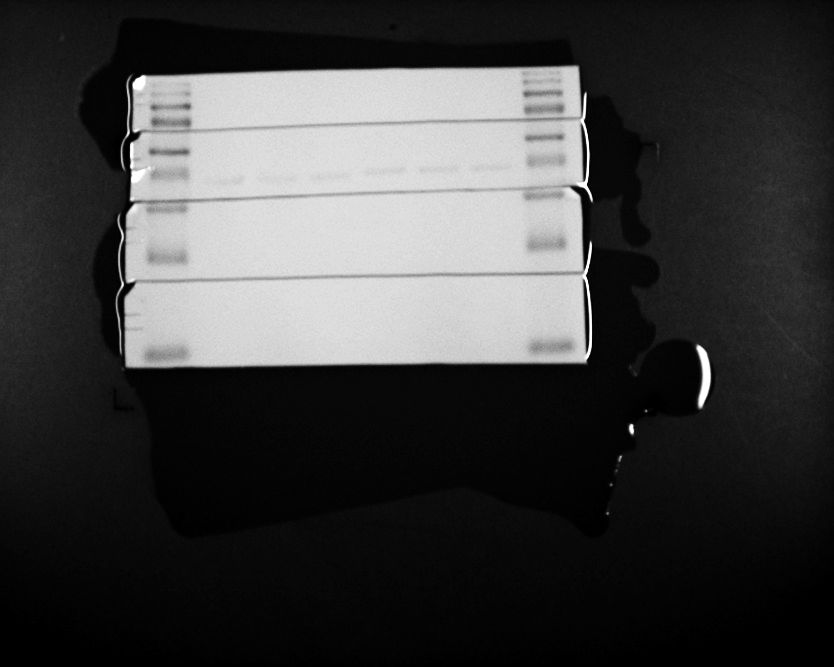

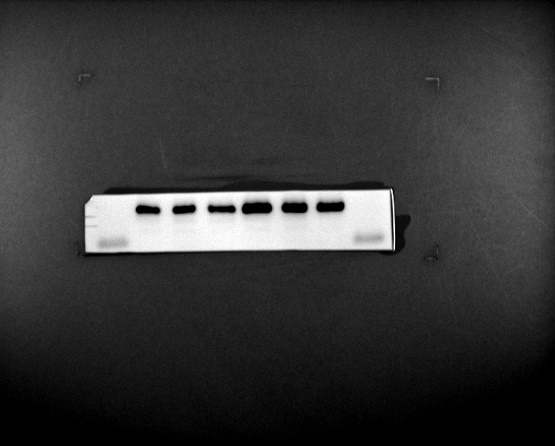


β-actin 42kD


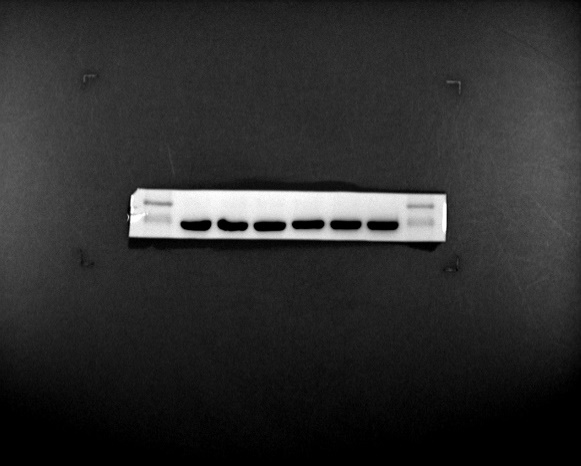


Bcl2 26kD


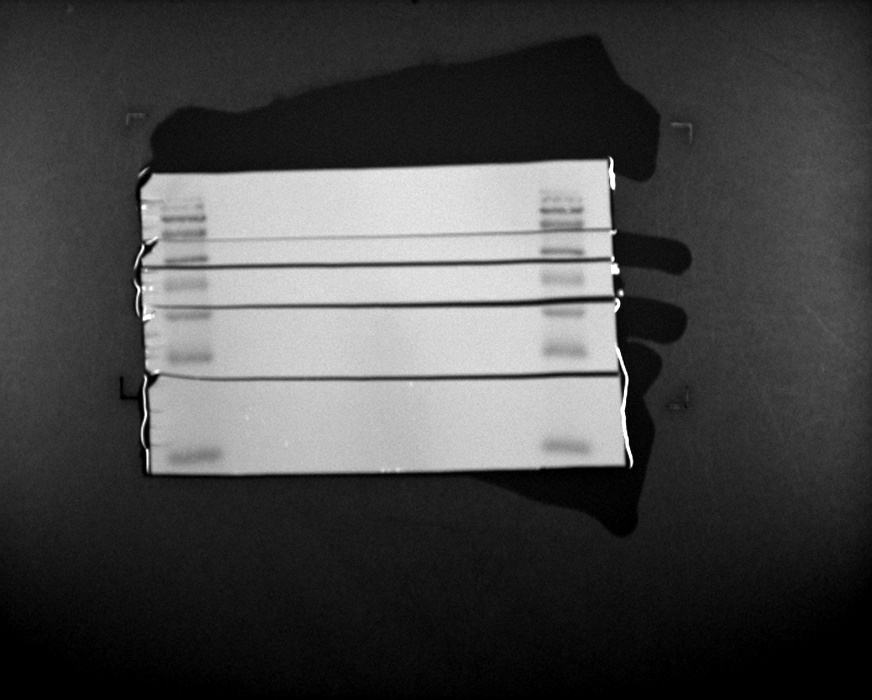

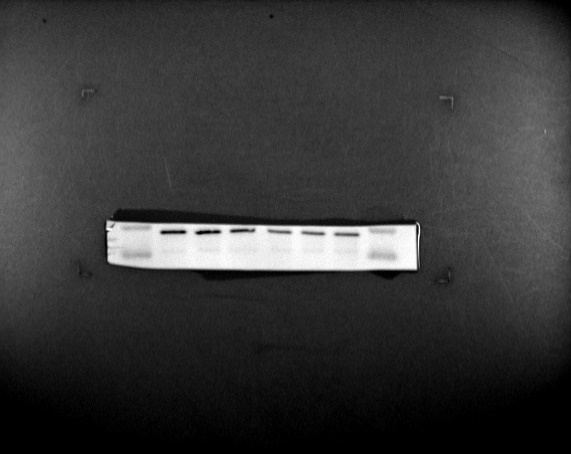


β-actin 42kD


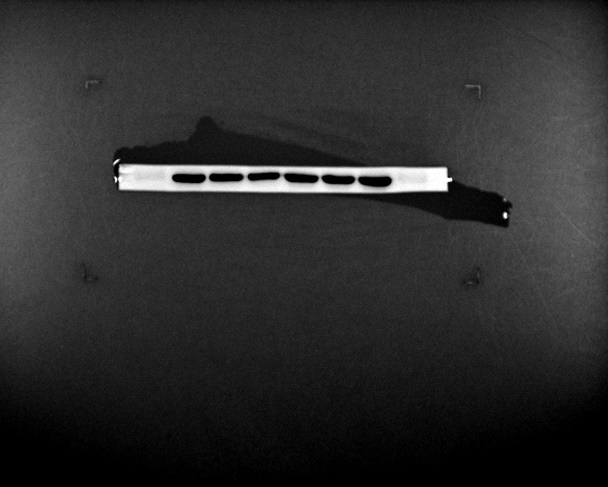


**Figure 7B.**

Nrf2 65kD


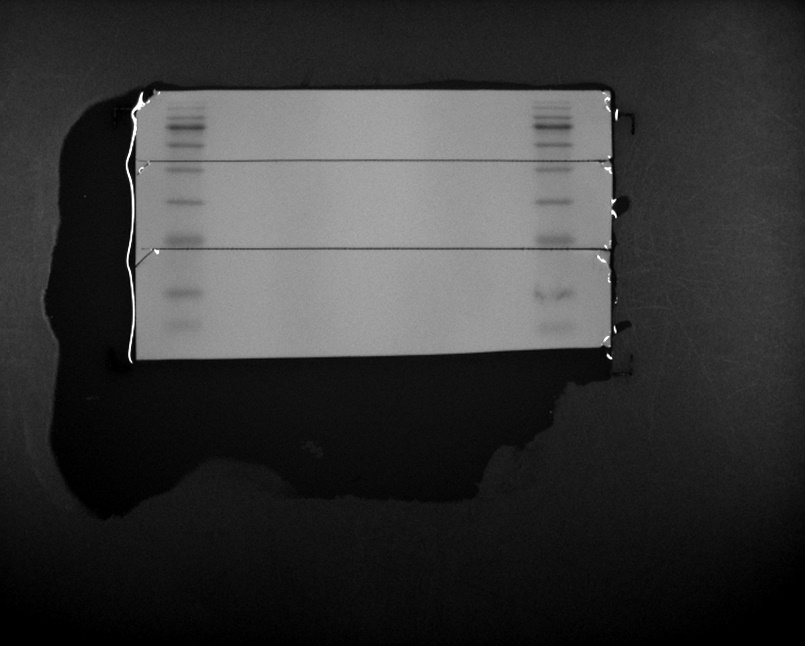

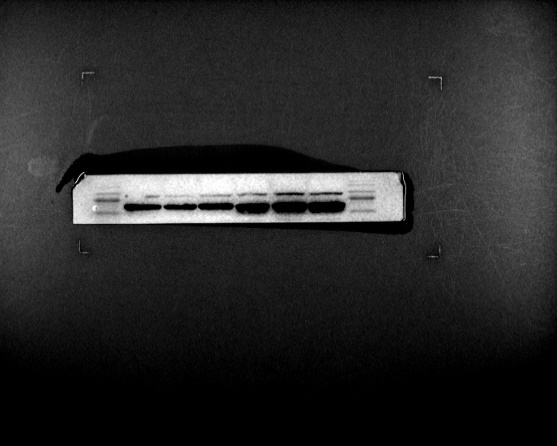


β-actin 42kD


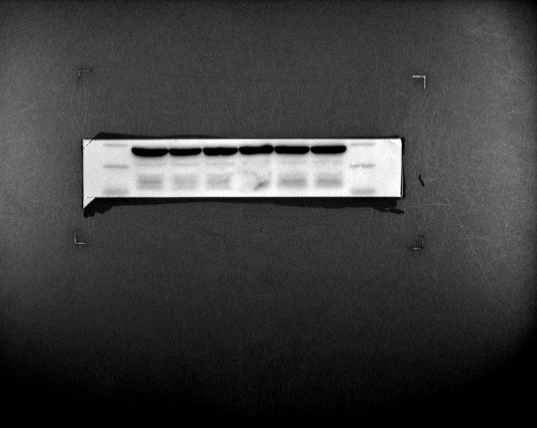


NQO1 31kD


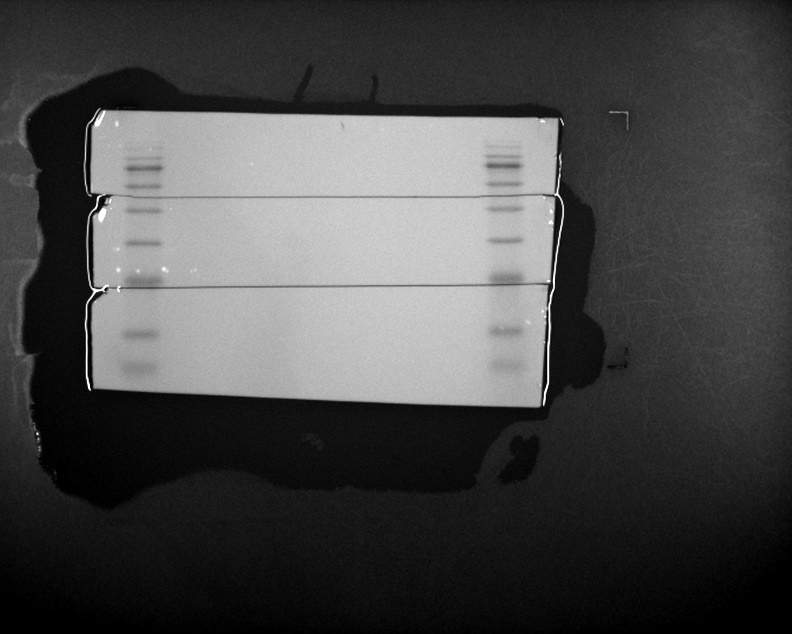

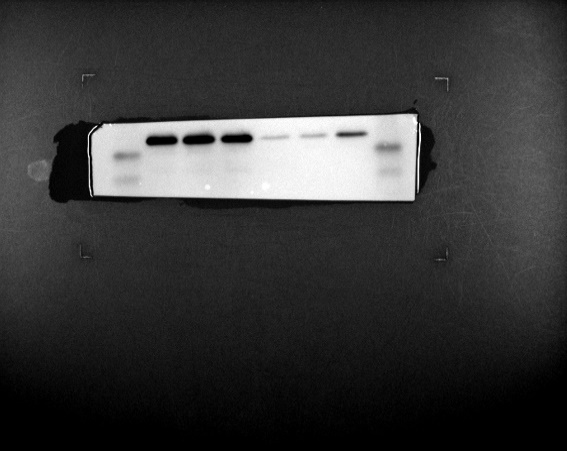


β-actin 42kD





SOD1 18kD


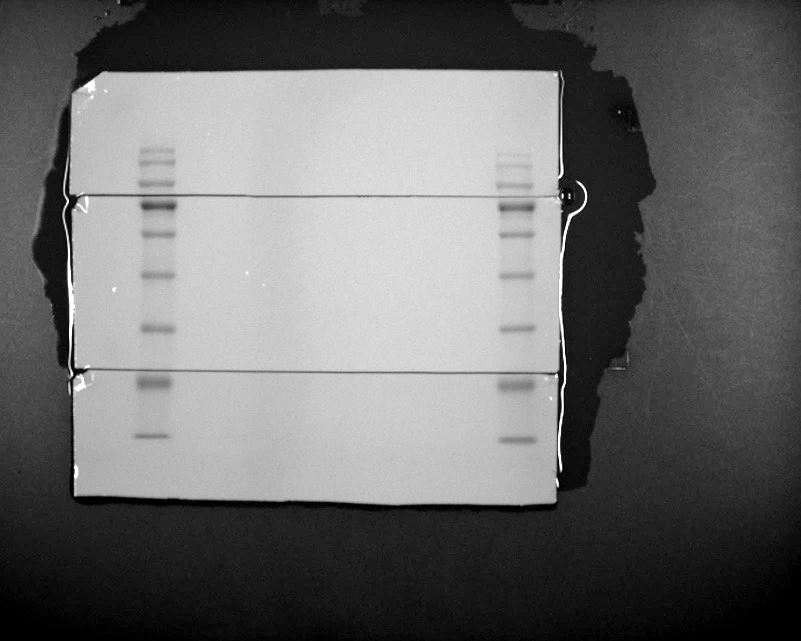

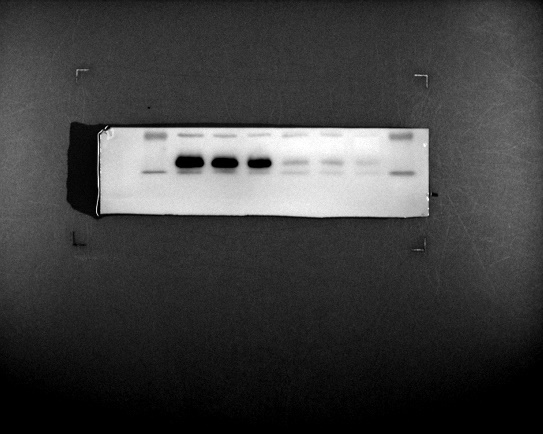


β-actin 42kD


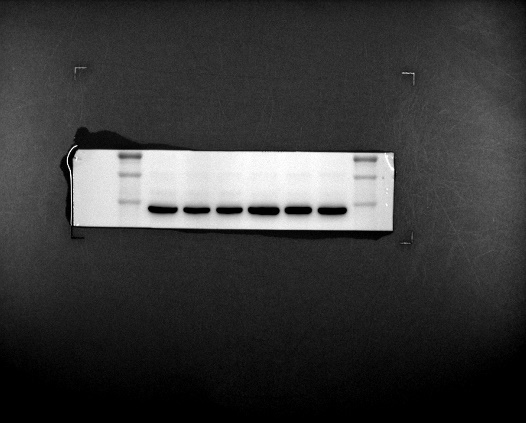


**Figure 7F.**

FTL 17kD


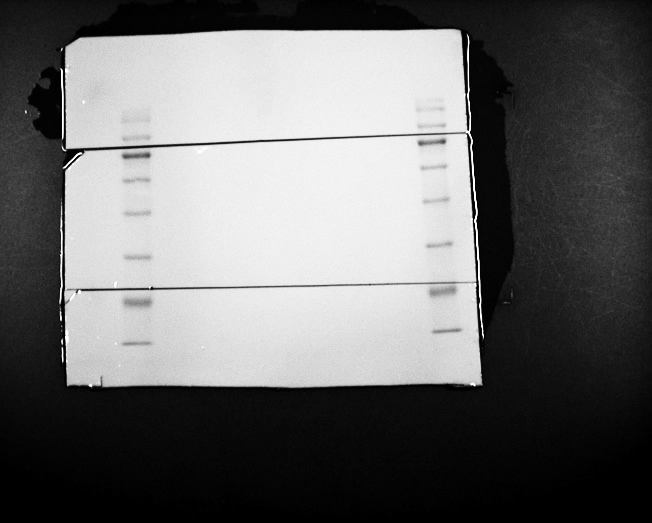

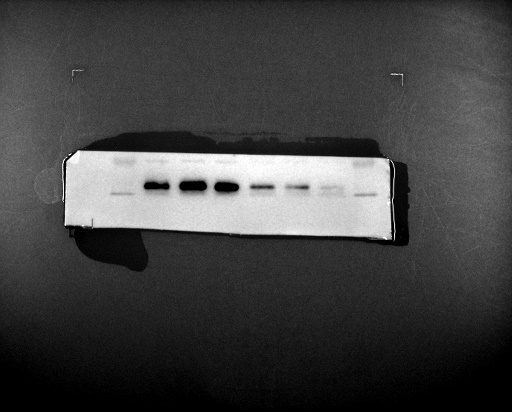


β-actin 42kD


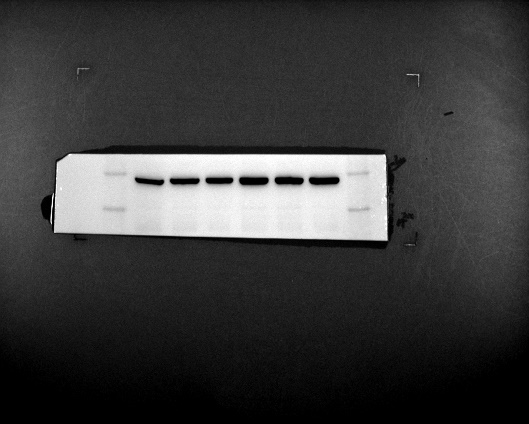


FRT 20kD


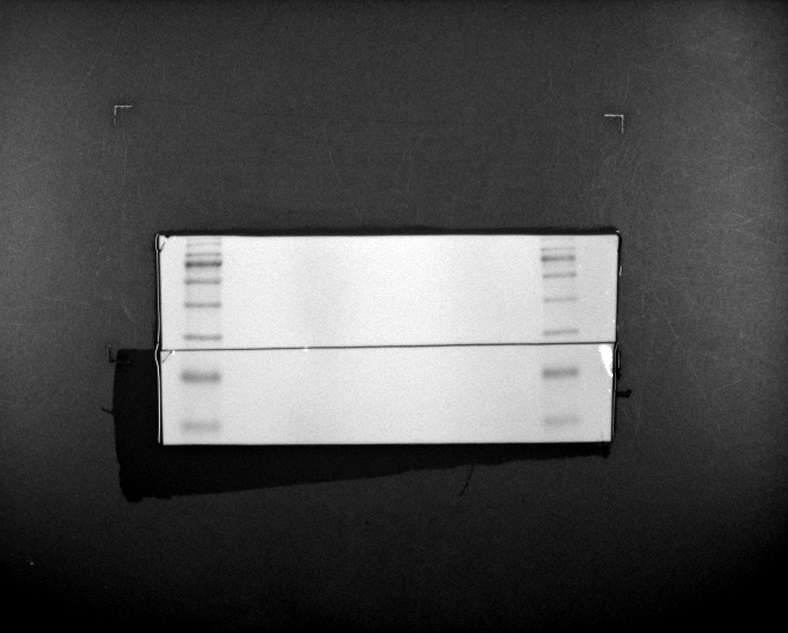




β-actin 42kD


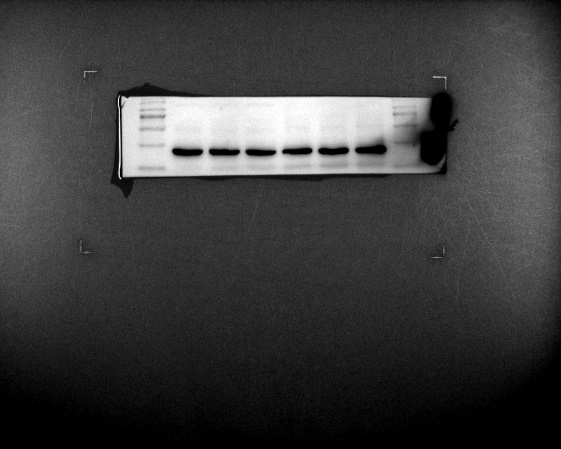


xCT 55kD


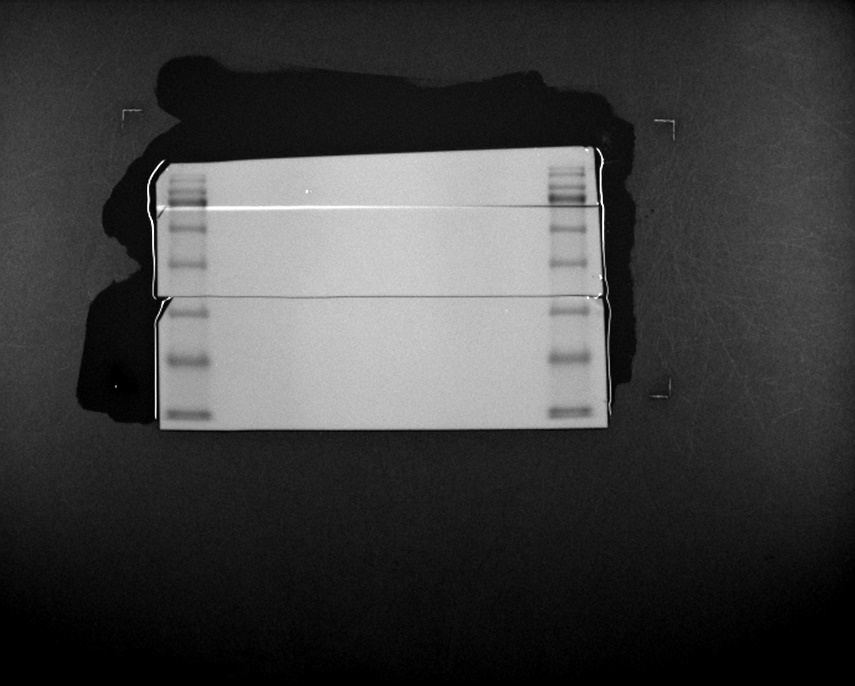

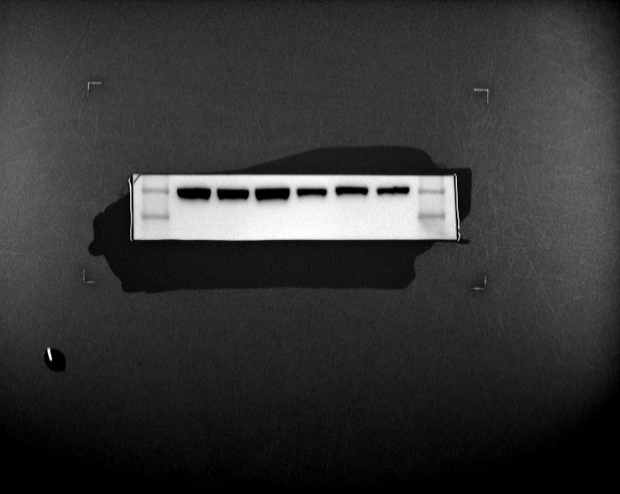


β-actin 42kD


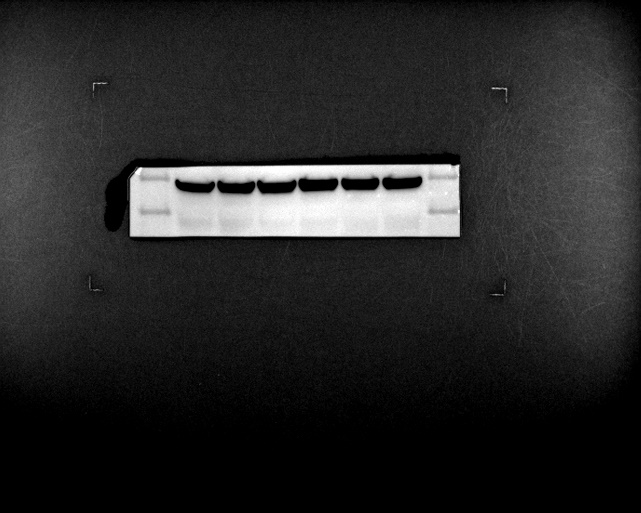


GPX4 17kD


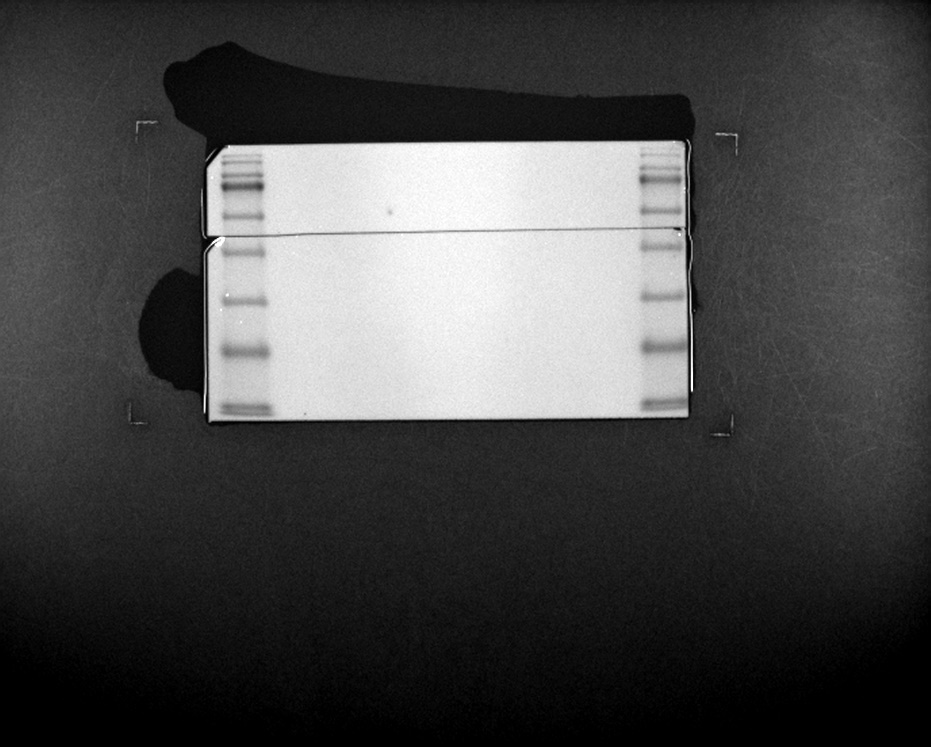




β-actin 42kD


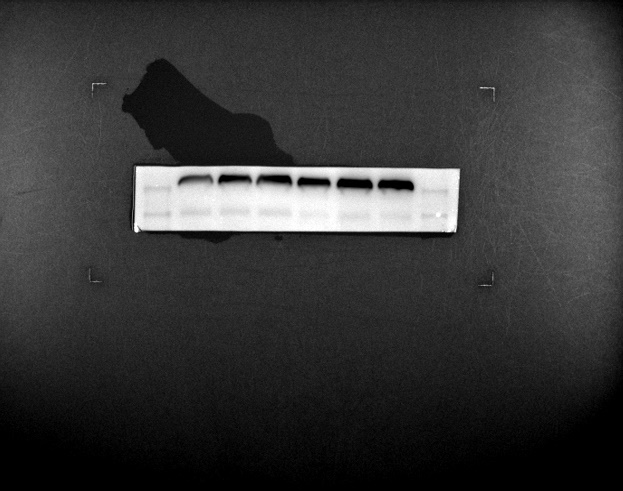


**Figure 8B.**

NQO1 31kD


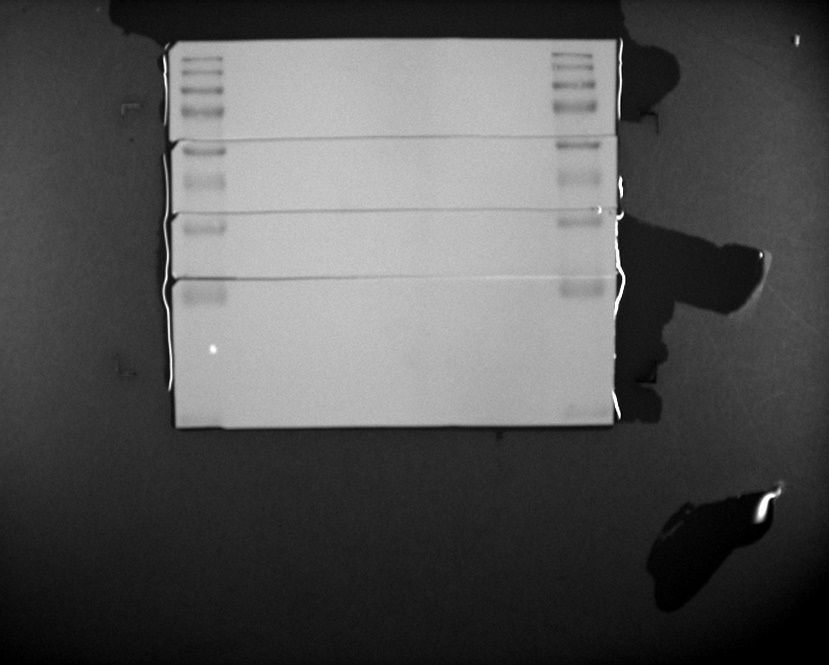

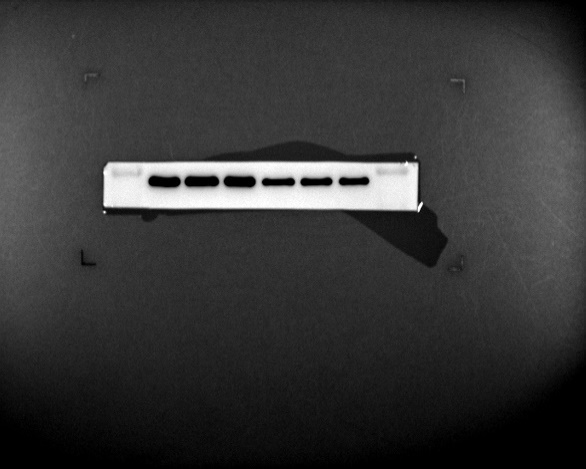


SOD1 18kD


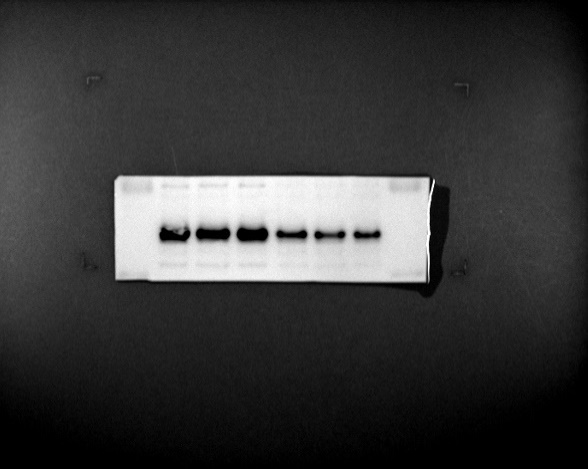


β-actin 42kD


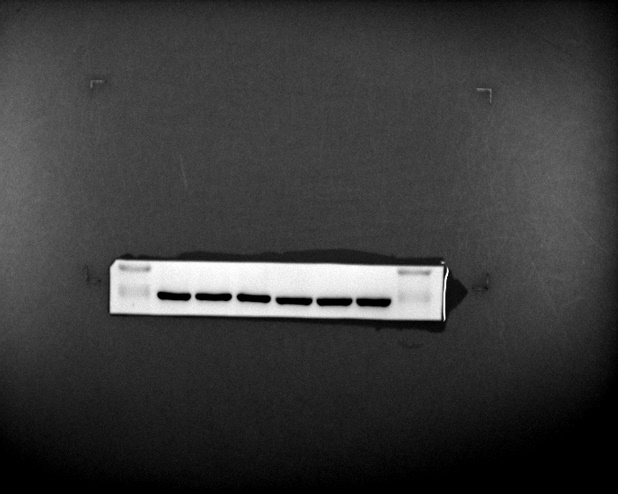


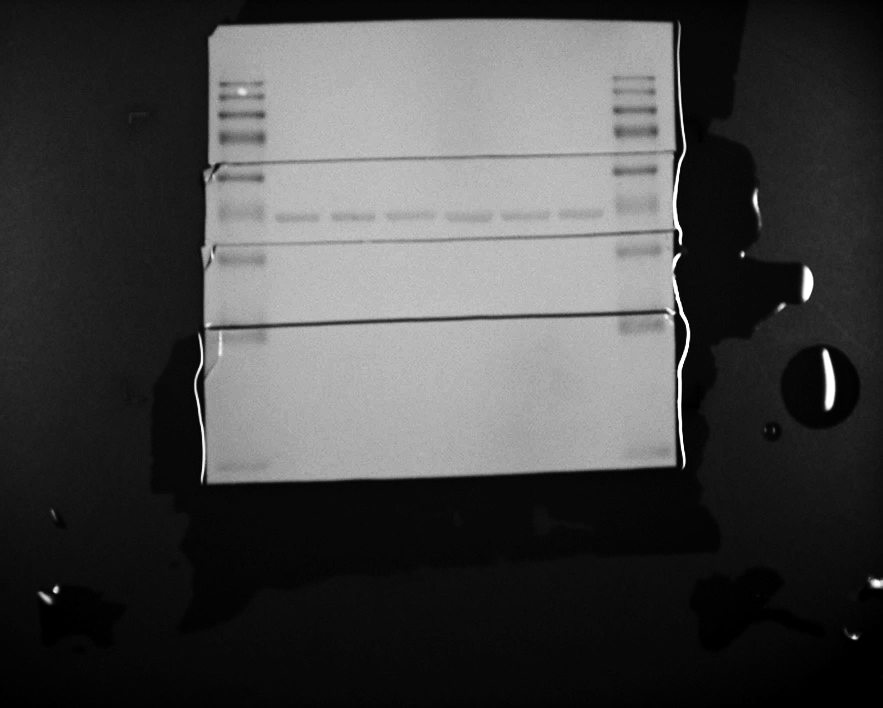
HO1 30kD


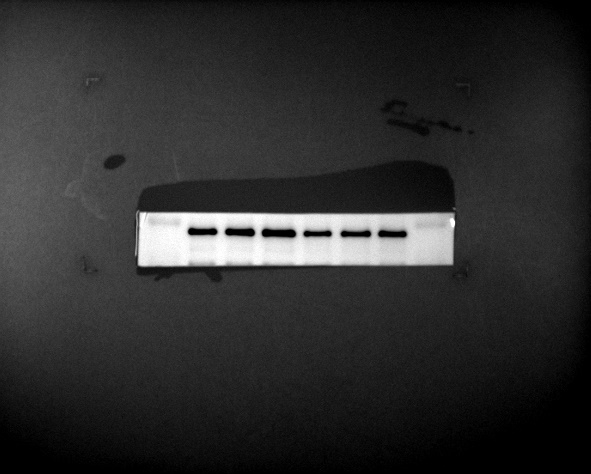


β-actin 42kD


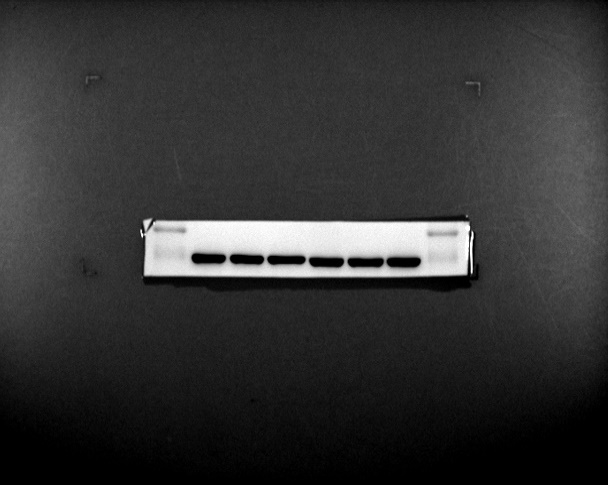


**Figure 8F.**

FRT 20kD


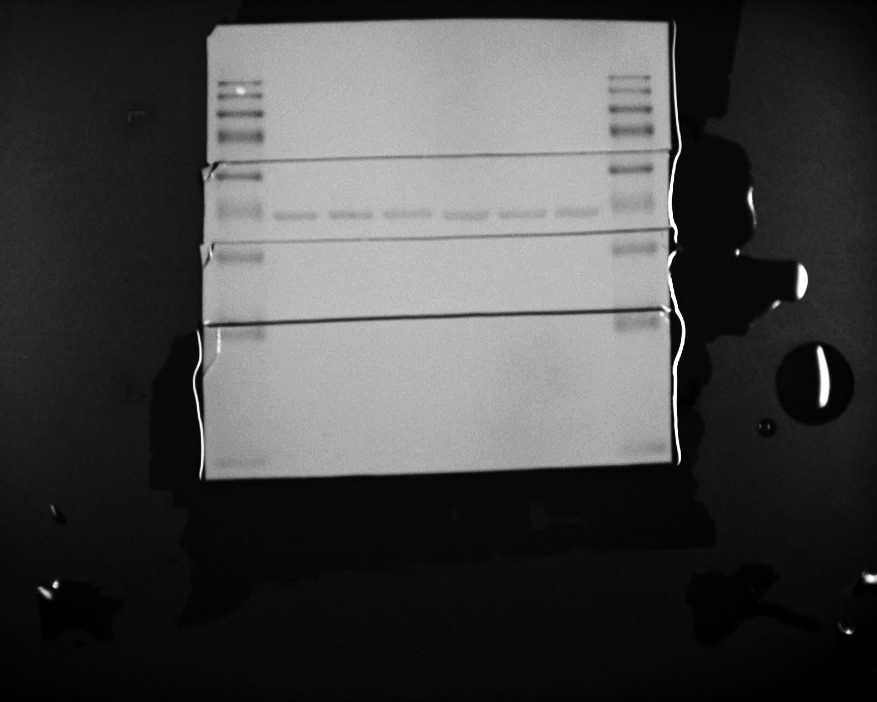

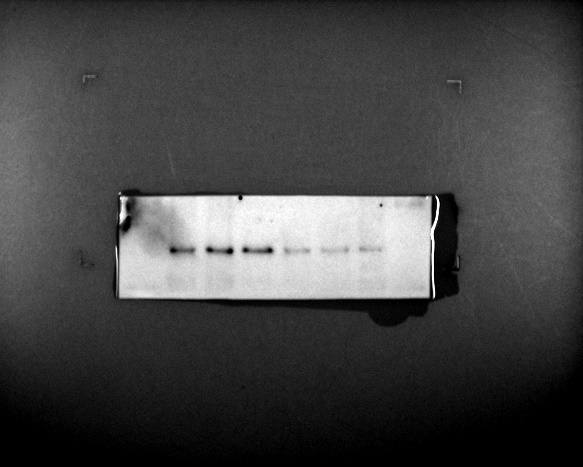


β-actin 42kD


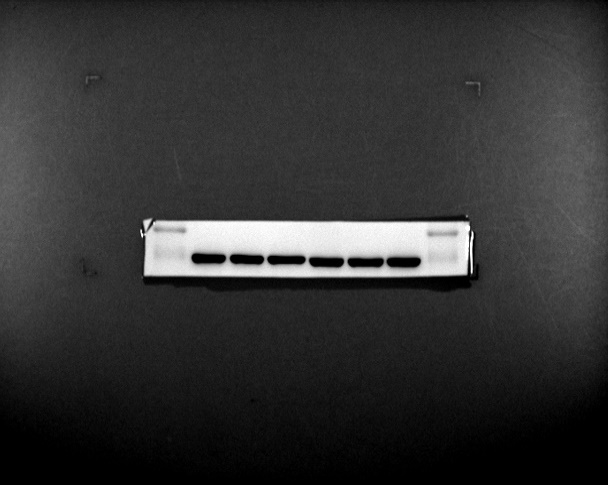


xCT 55kD


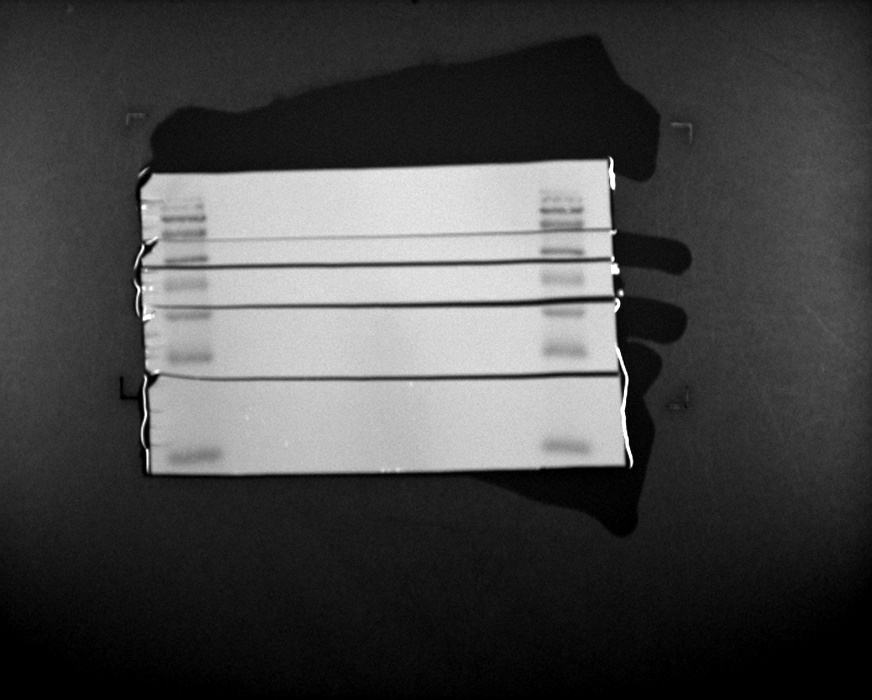

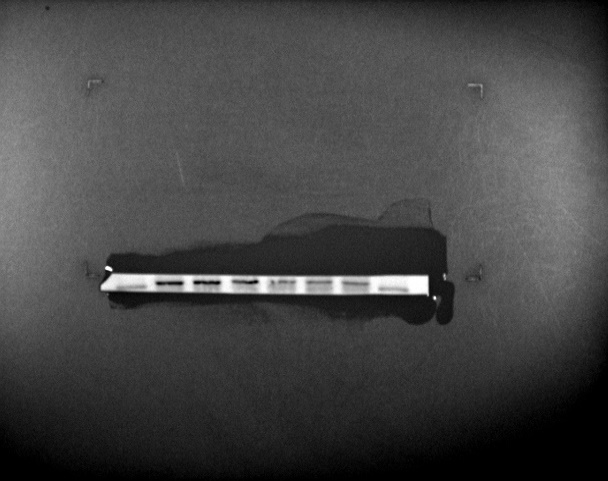


FTL 17kD


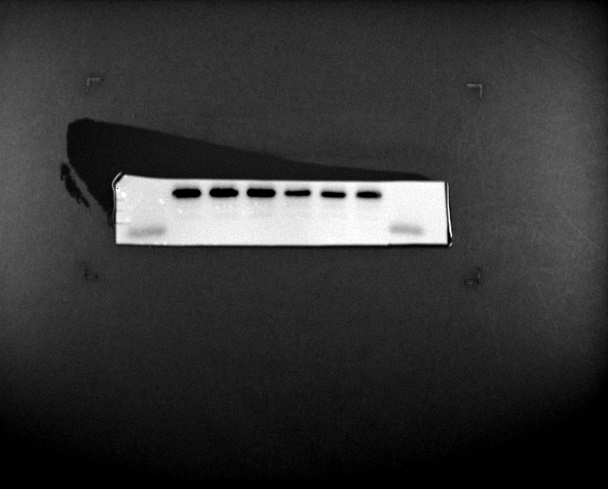


β-actin 42kD


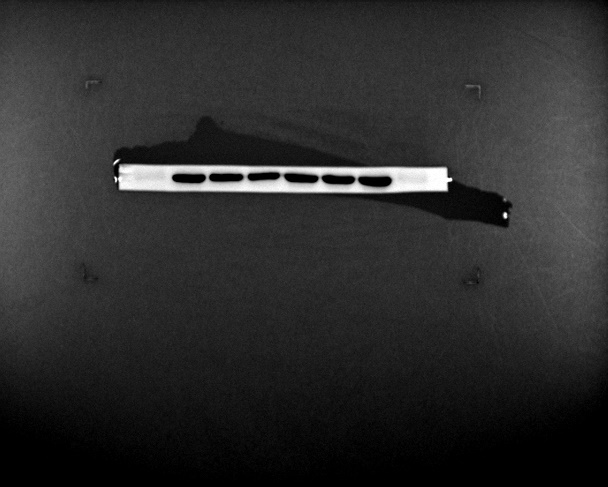


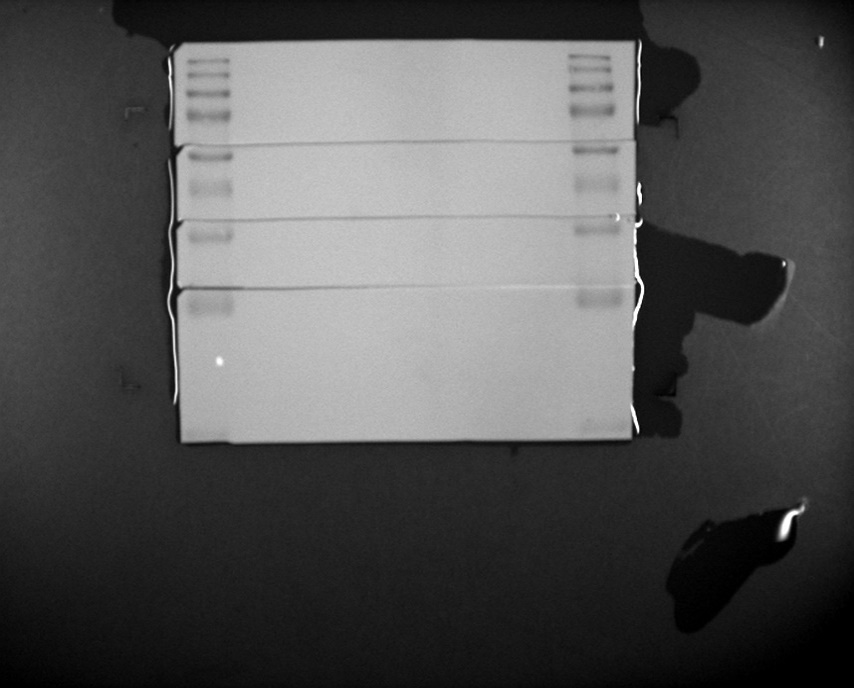
GPX4 17kD


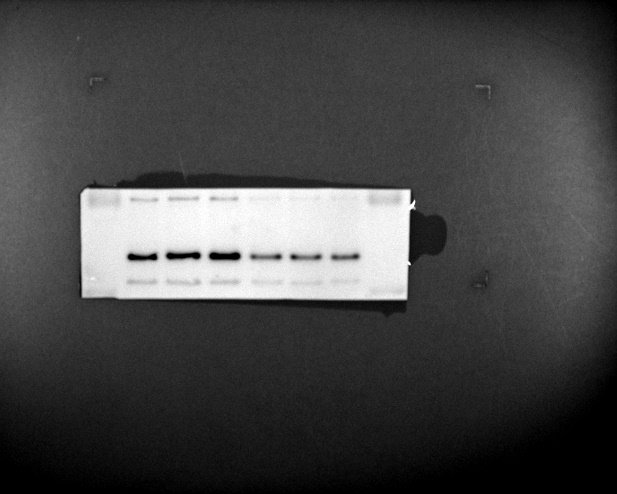


β-actin 42kD


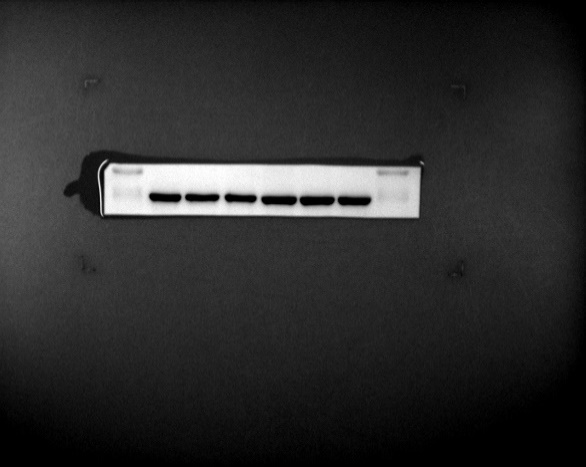


**Figure S7A.** **pulmonary cell nucleus**

Nrf2 65kD


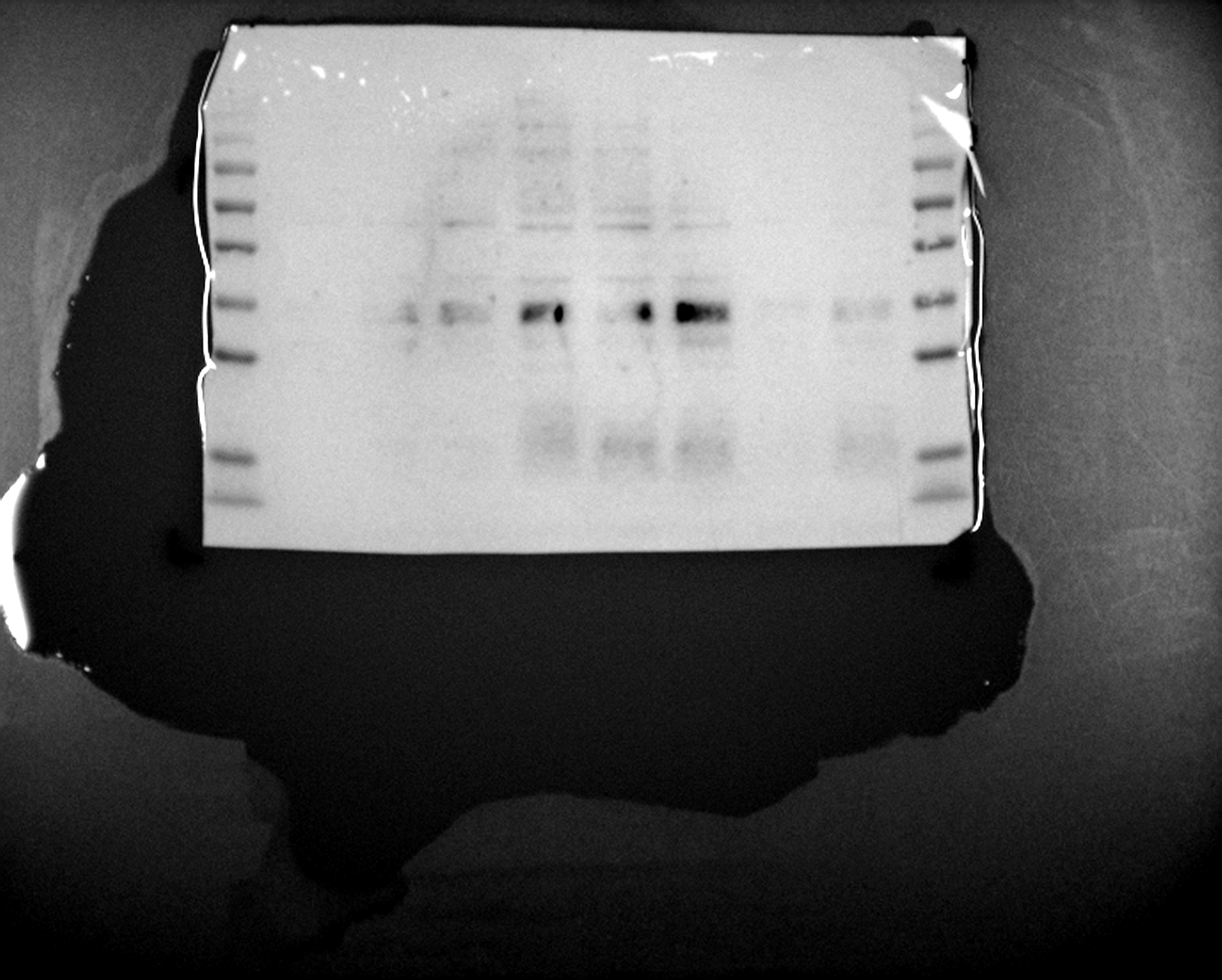


H2B 18KD


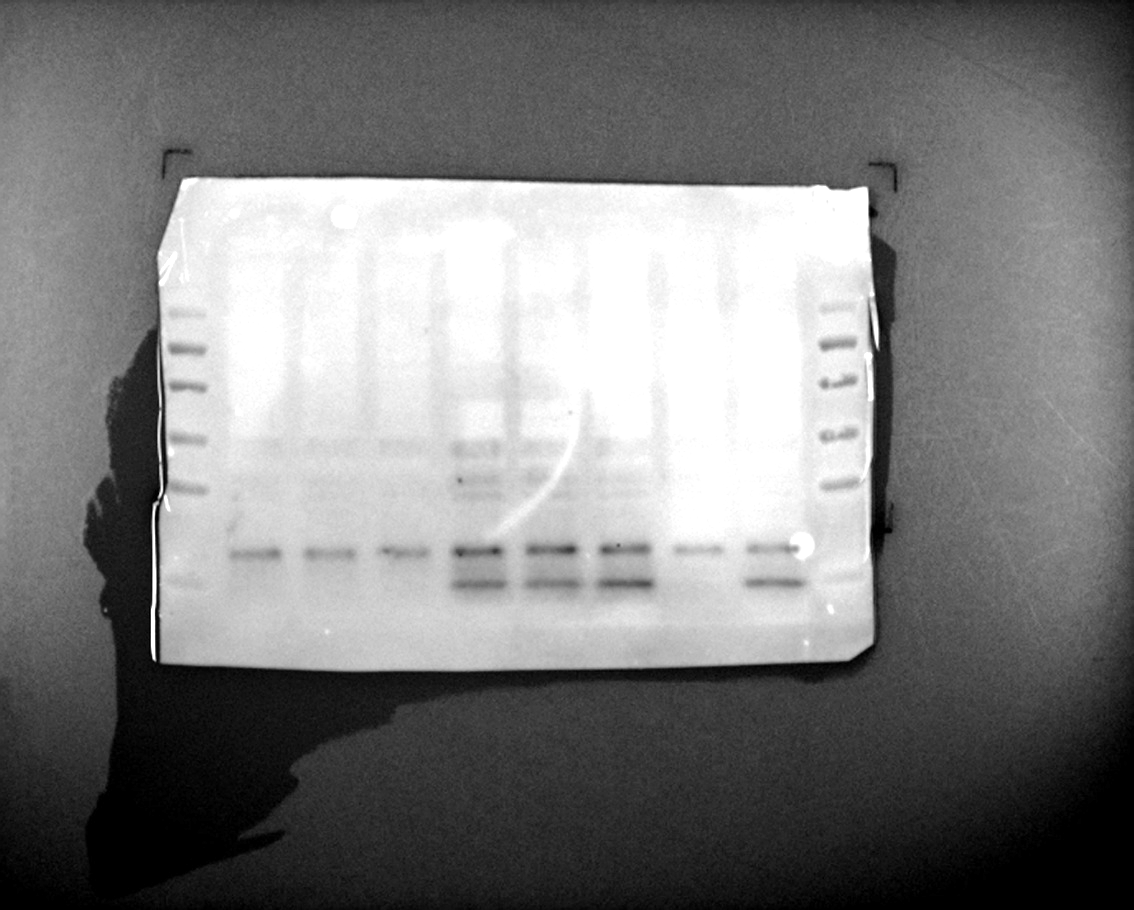


β-actin 42KD


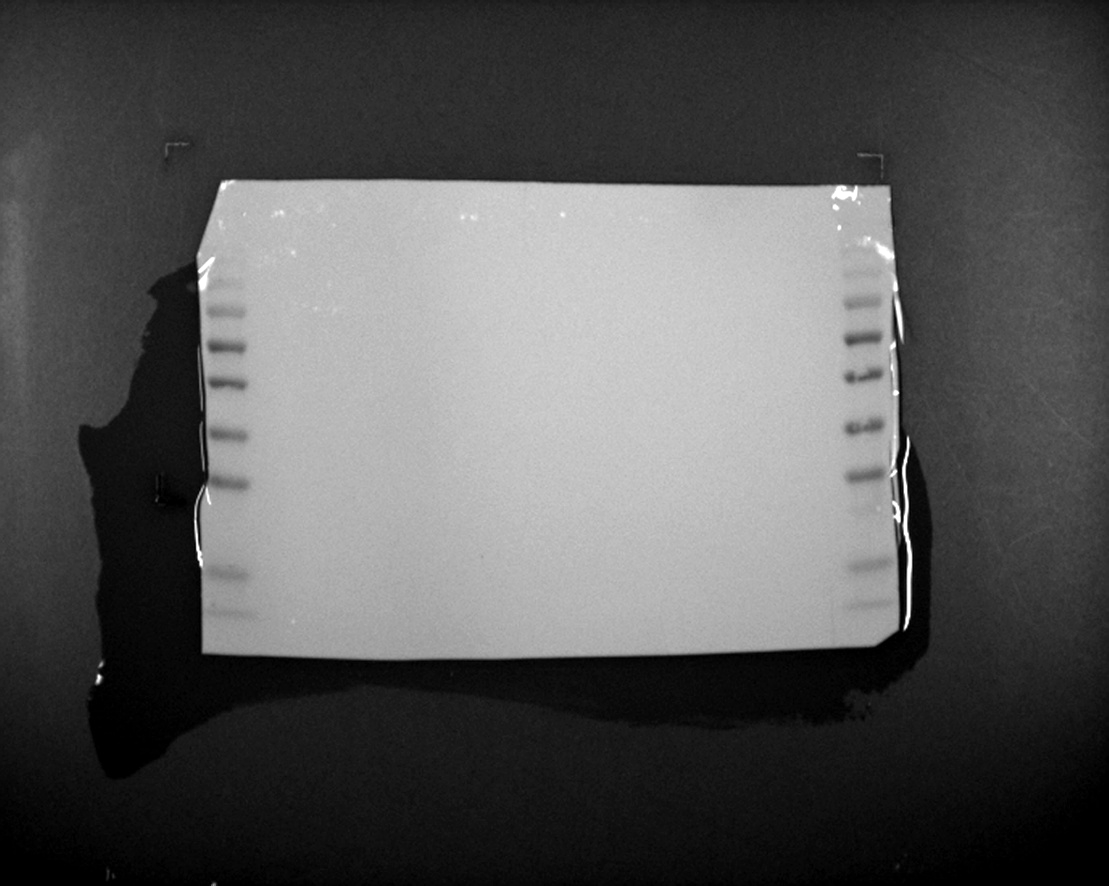


**Figure S7A.** **pulmonary cell cytoplasm**

Nrf2 65kD


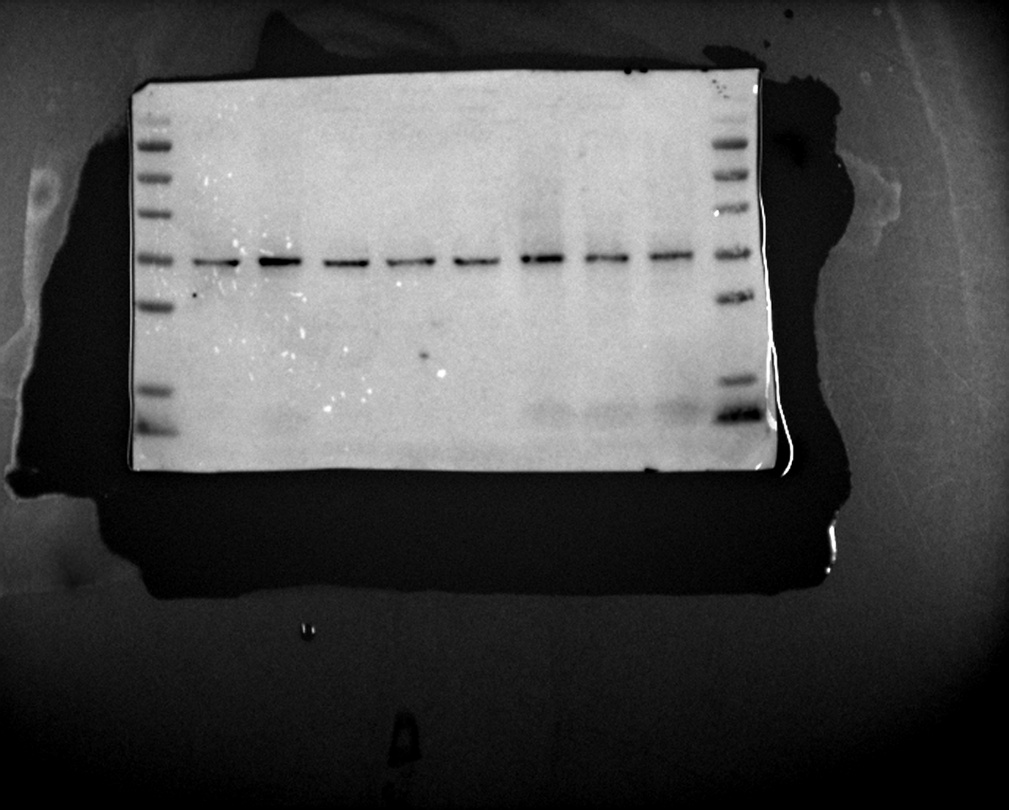


H2B 18KD


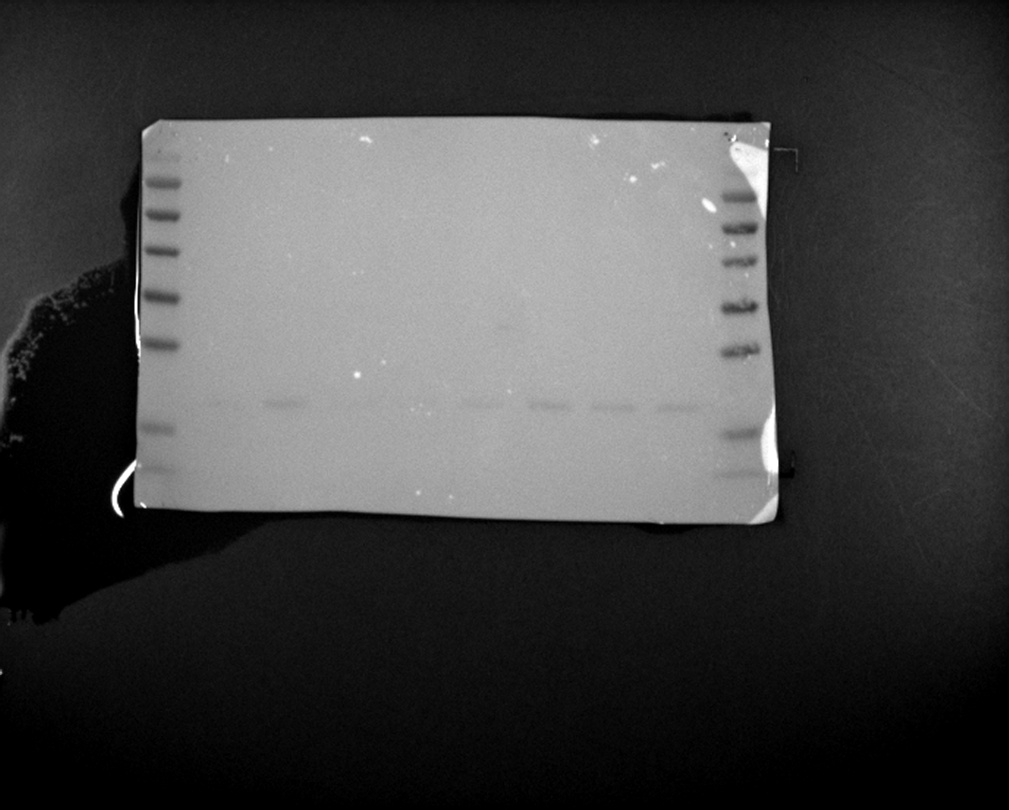


β-actin 42KD

**
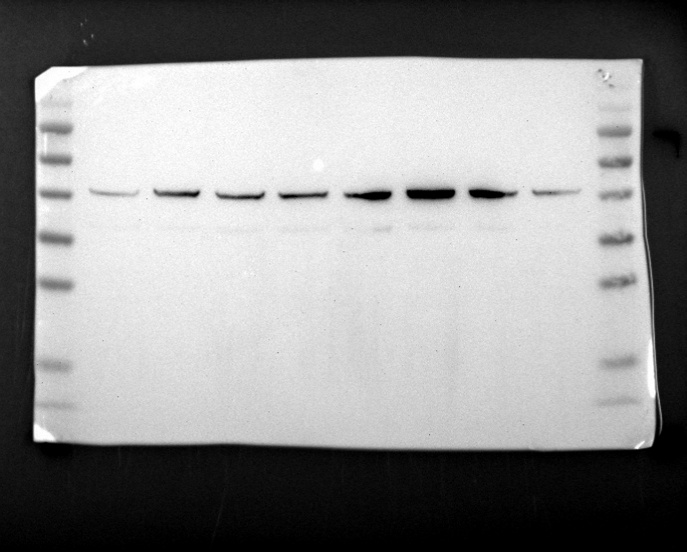
**

**Figure S7B.** **hepatocyte nucleus**

Nrf2 65KD


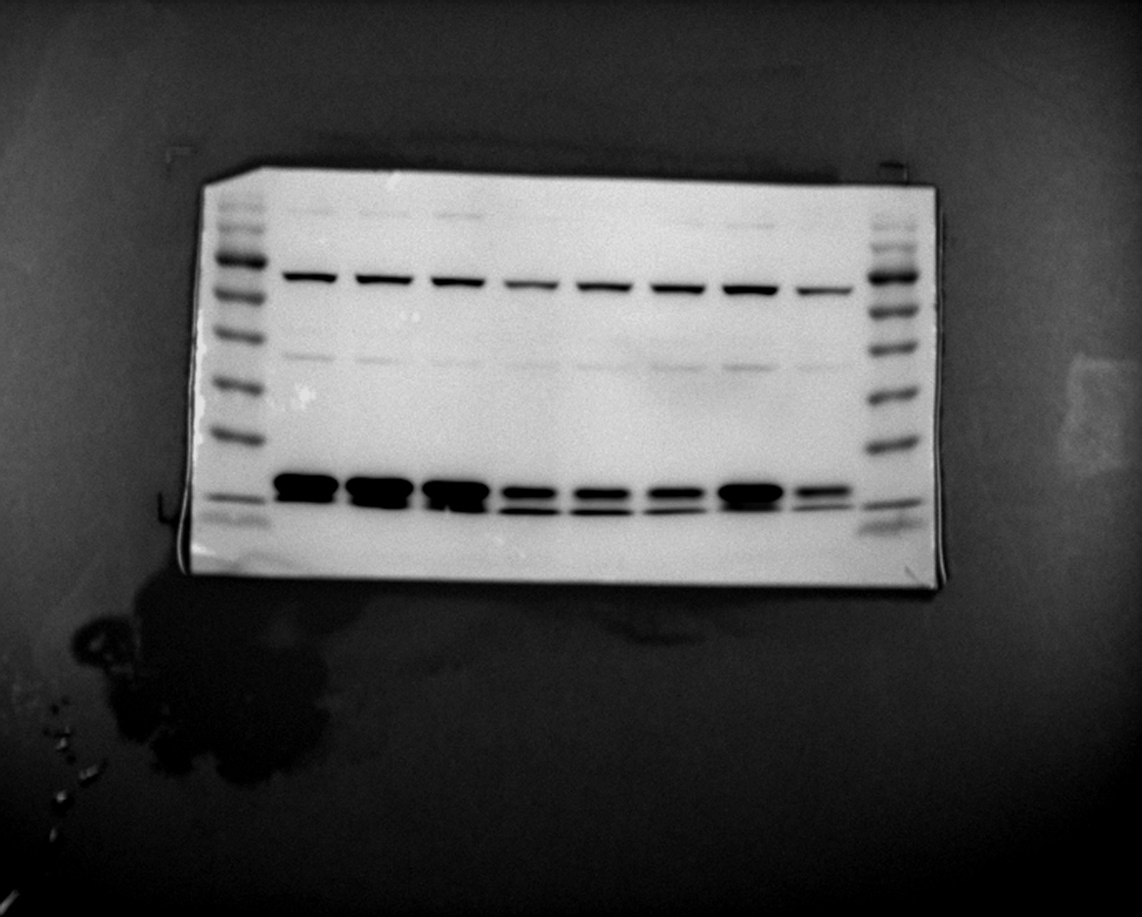


H2B 18KD


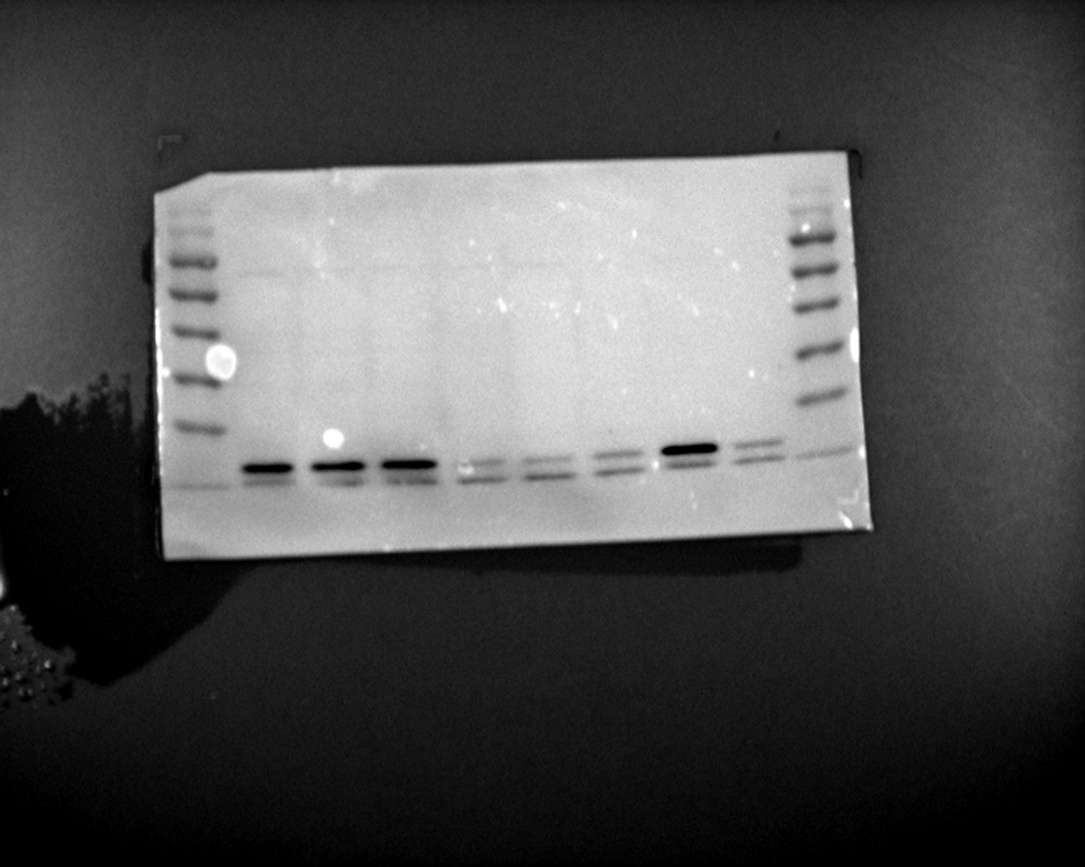


β-actin 42KD

**
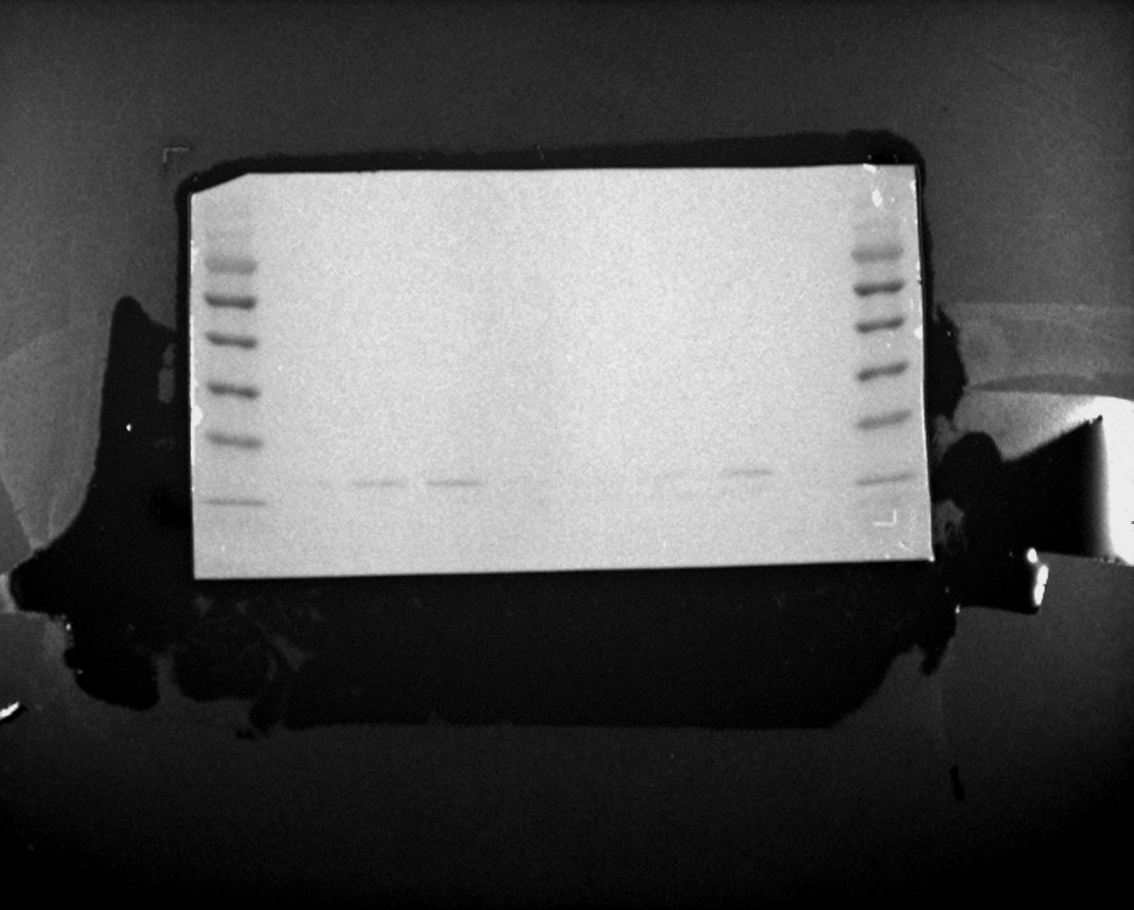
**

**Figure S7B.** **hepatocyte cytoplasm**

Nrf2 65KD


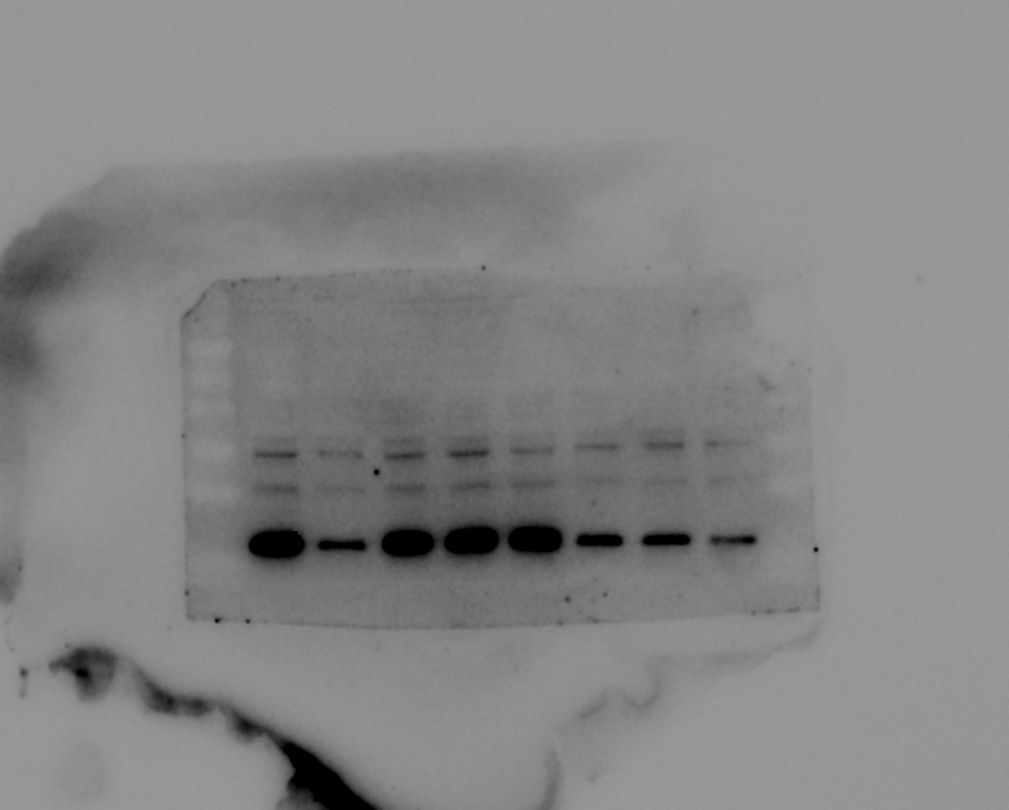


H2B 18KD


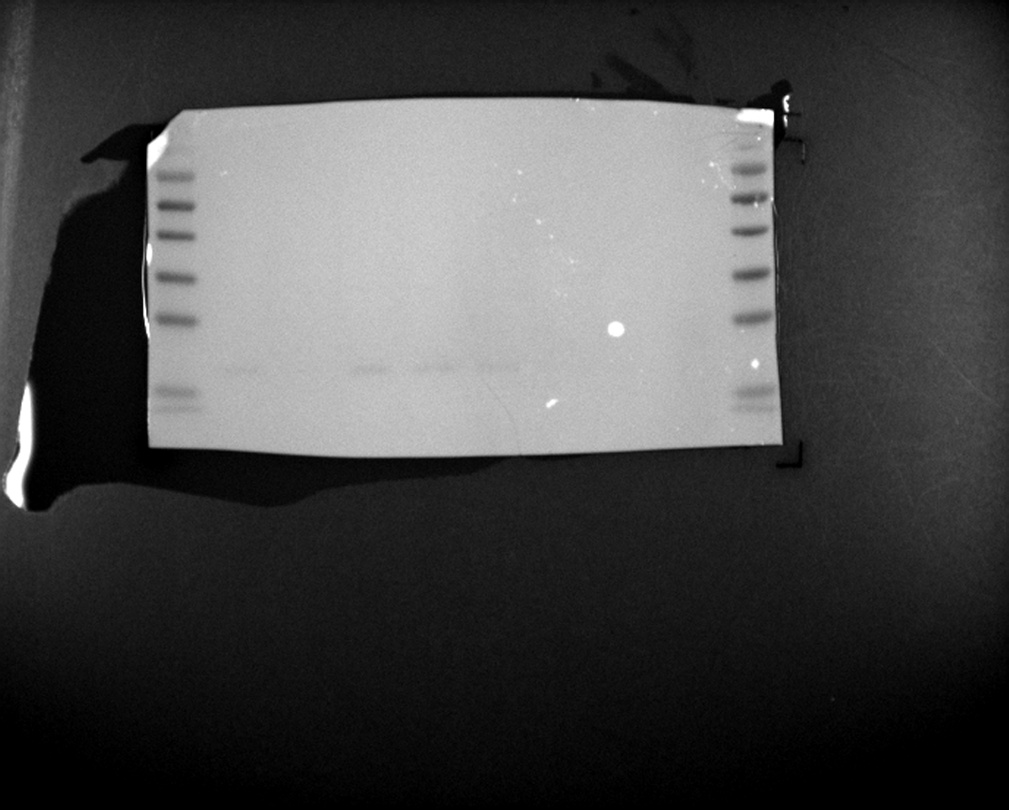


**Figure S8C**

FRT 20kD


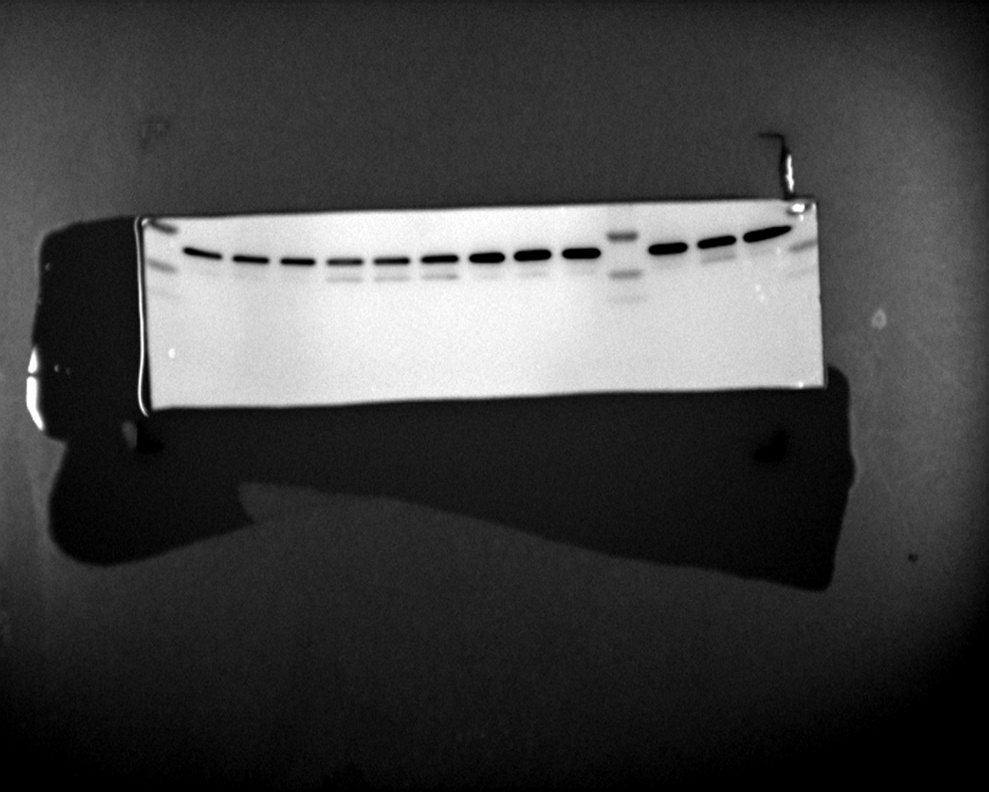


xCT 55kD


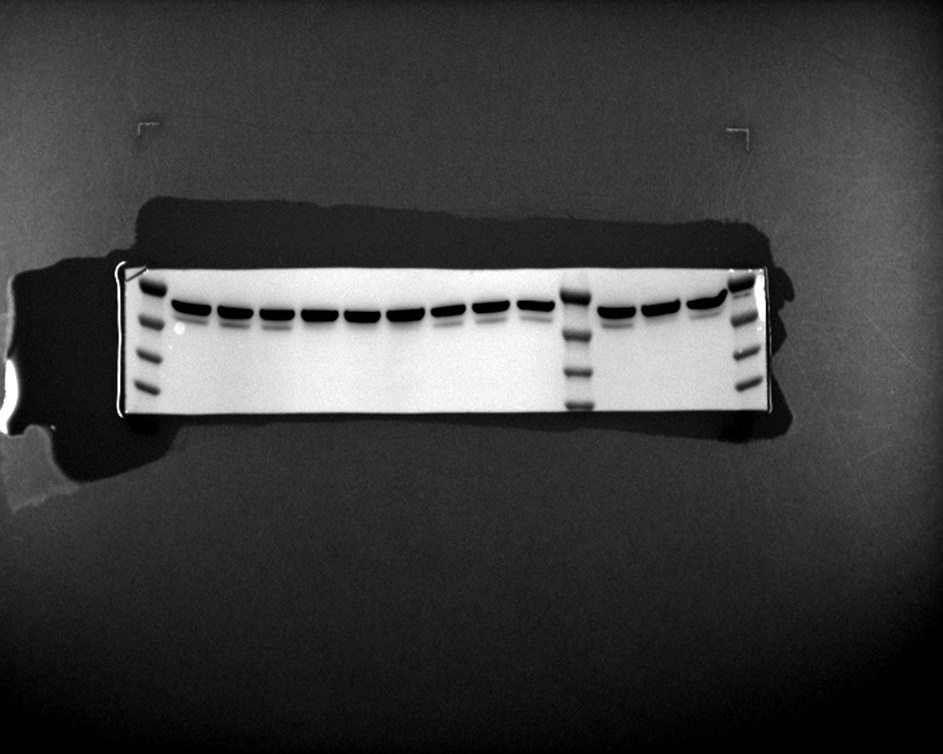


GPX4 17kD


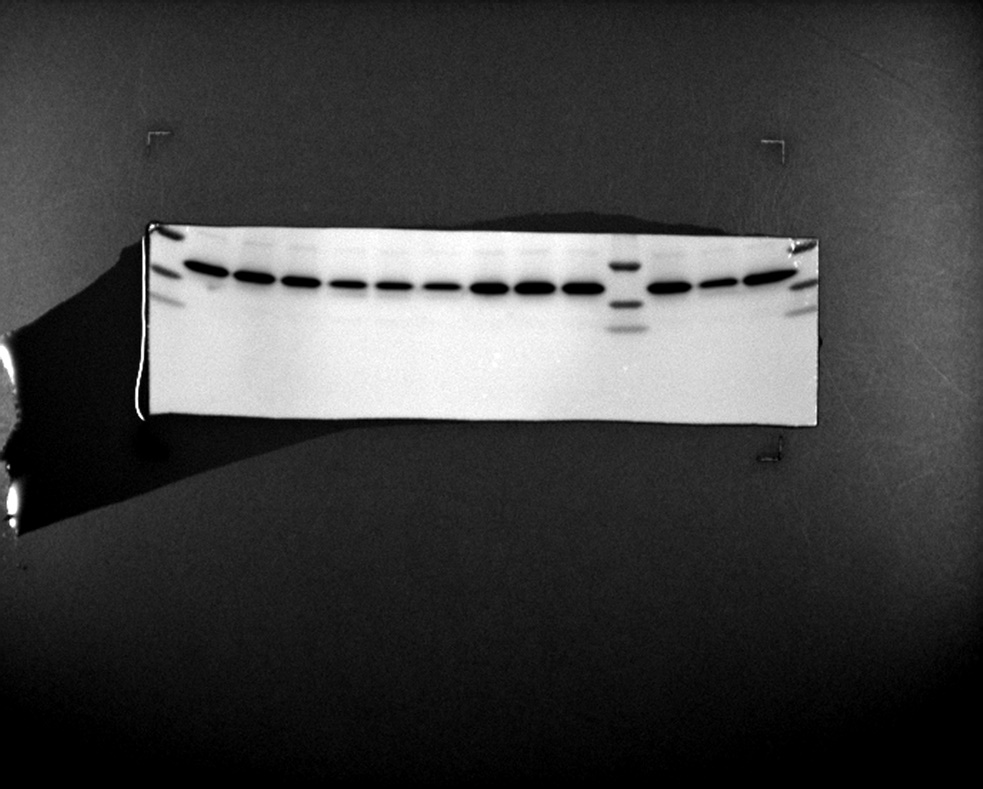


β-actin 42kD


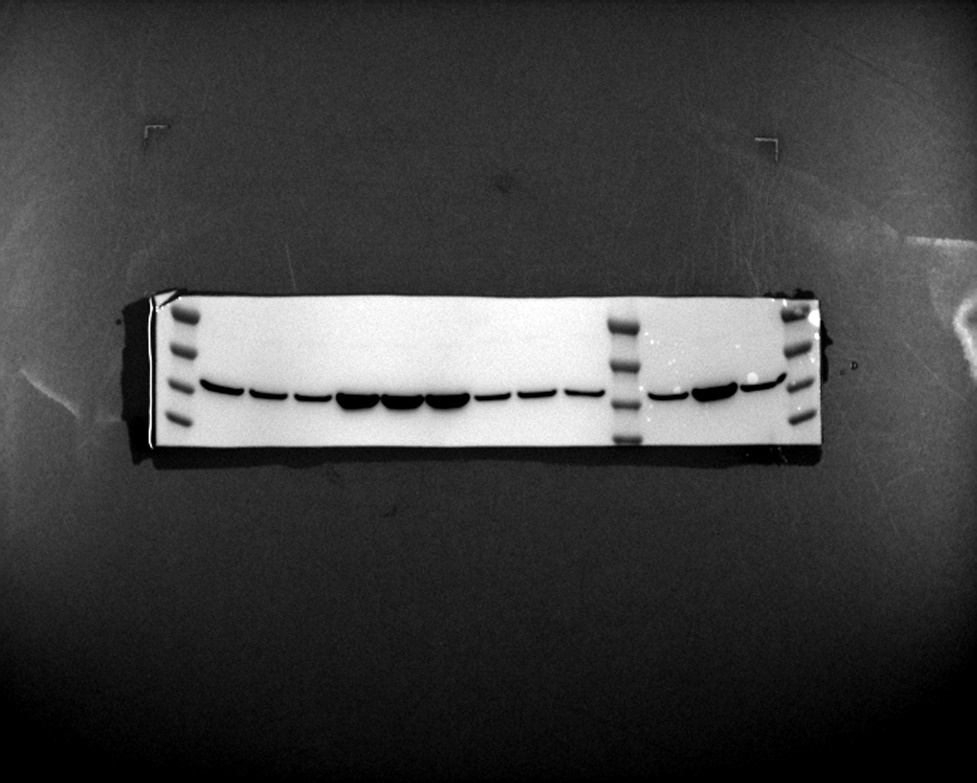


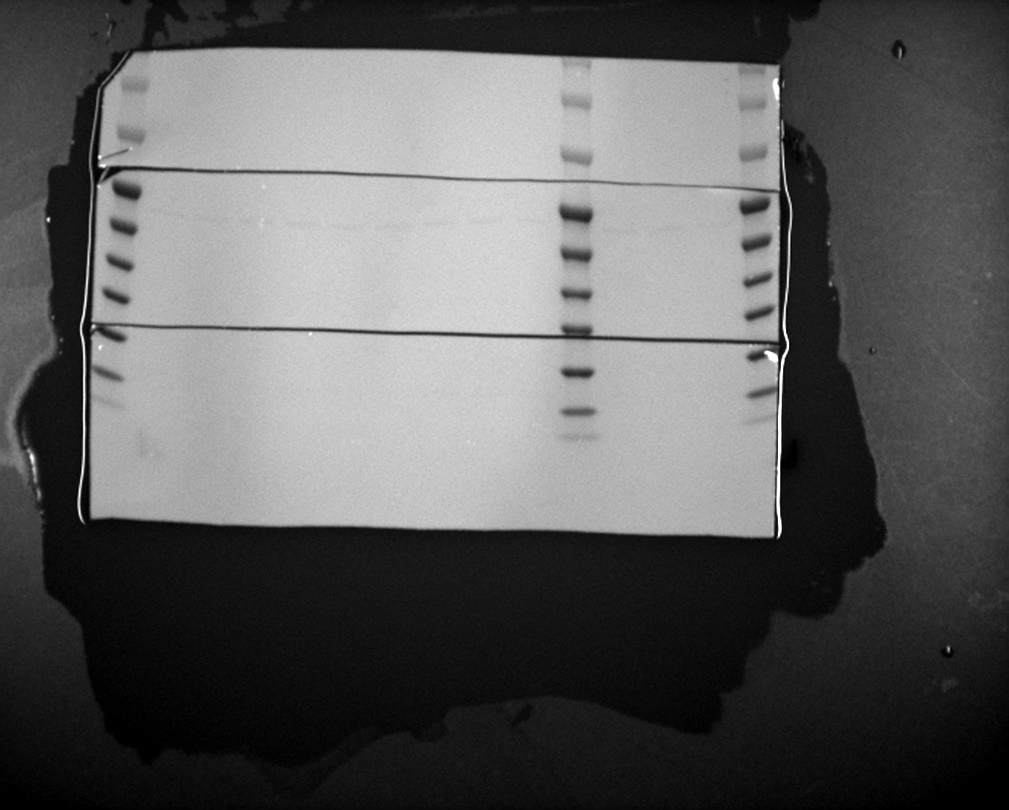


**Figure S9B**

FRT 20kD

**
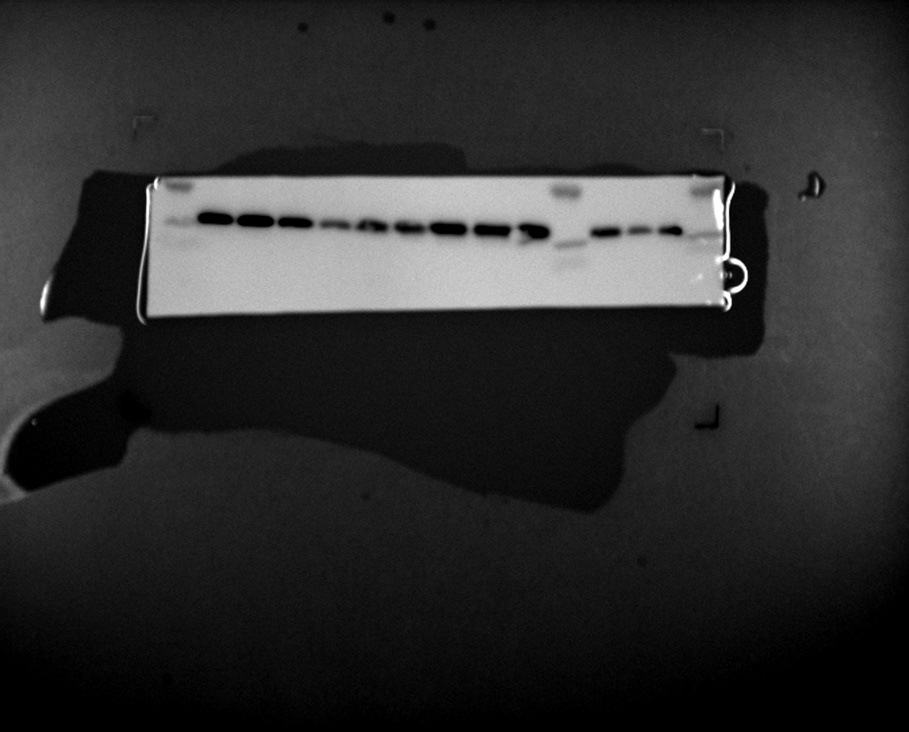
**

xCT 55kD


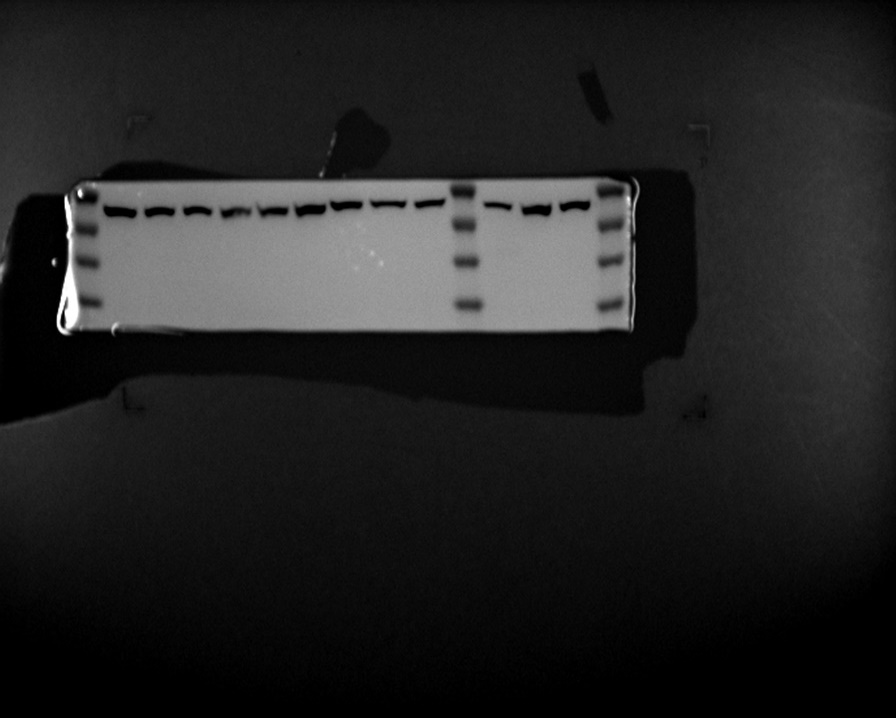


GPX4 17kD


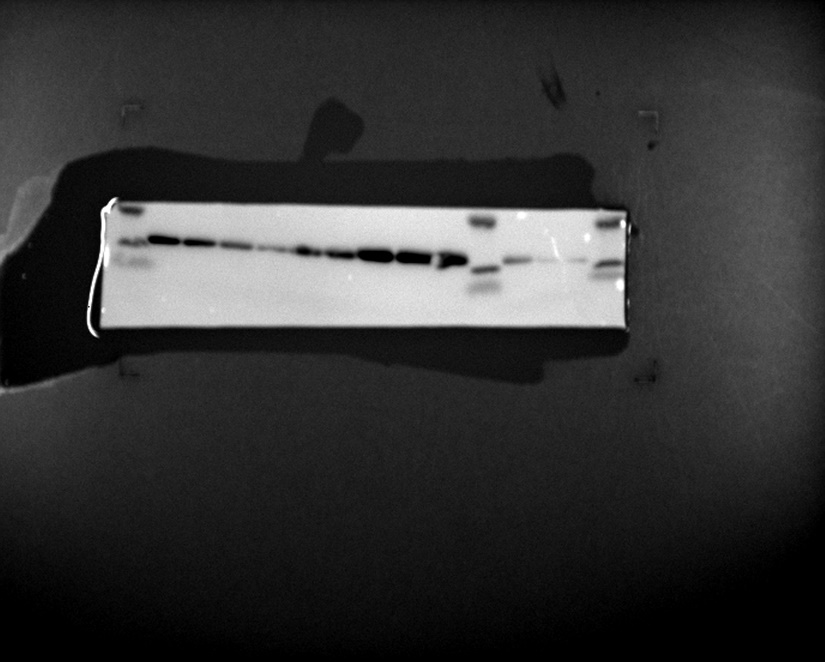


β-actin 42kD


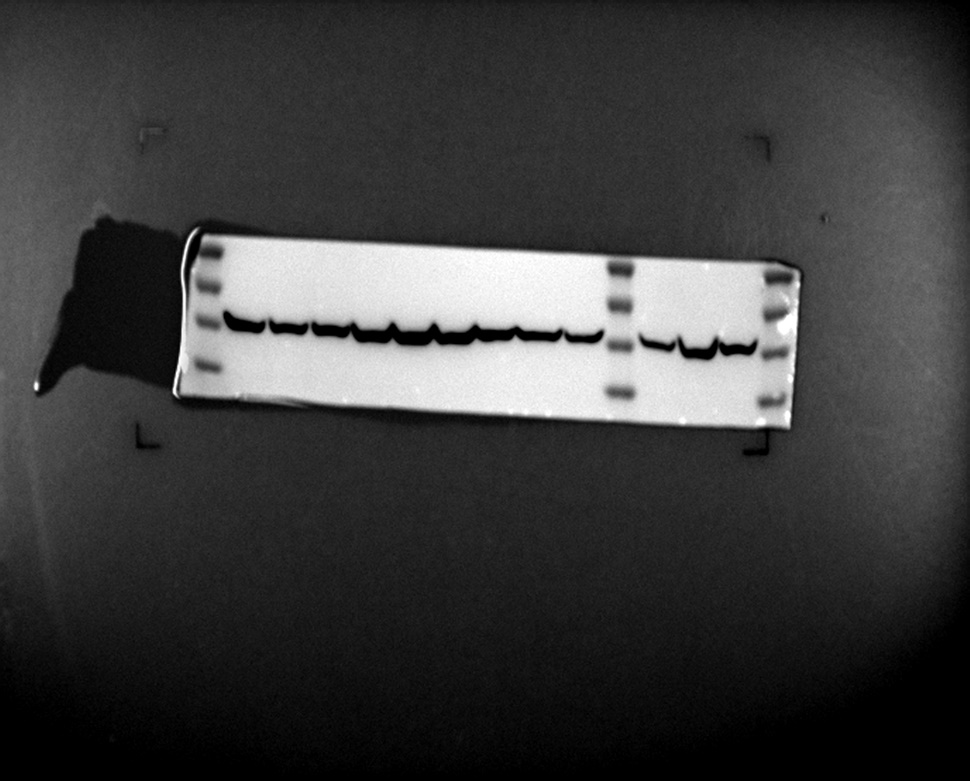


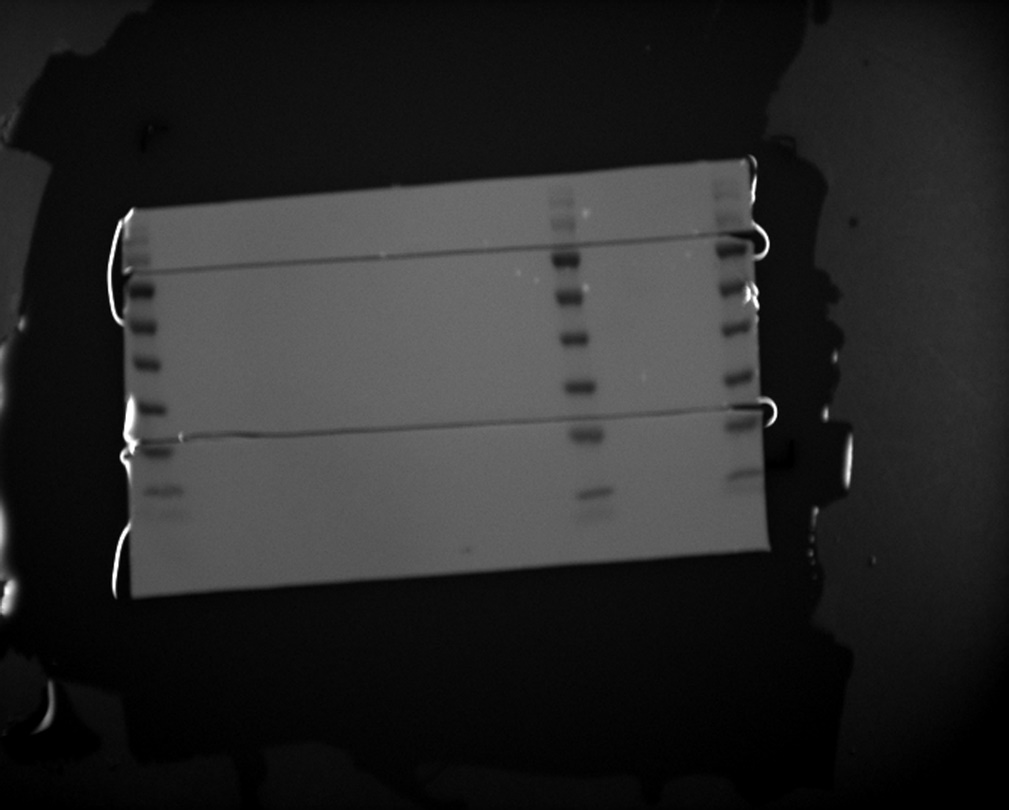

Supplement: Supplementary file 2 — Data S2: Supporting Information. [file CPR-59-e70117-s001.docx]
